# Hidden Markov Model*ing* with HMMTeacher

**DOI:** 10.1371/journal.pcbi.1009703

**Published:** 2022-02-10

**Authors:** Camilo Fuentes-Beals, Alejandro Valdés-Jiménez, Gonzalo Riadi

**Affiliations:** 1 PhD Program in Sciences Mention Modeling of Chemical and Biological Systems, School of Bioinformatics Engineering, Center for Bioinformatics, Simulation and Modeling, CBSM, Department of Bioinformatics, Faculty of Engineering, University of Talca, Campus Talca, Talca, Chile; 2 Center for Bioinformatics, Simulation and Modeling, CBSM, Department of Bioinformatics, Faculty of Engineering, University of Talca, Campus Talca, Talca, Chile; 3 ANID–Millennium Science Initiative Program Millennium Nucleus of Ion Channels-Associated Diseases (MiNICAD), Center for Bioinformatics, Simulation and Modeling, CBSM, Department of Bioinformatics, Faculty of Engineering, University of Talca, Talca, Chile; SIB Swiss Institute of Bioinformatics, SWITZERLAND

## Abstract

Is it possible to learn and create a first Hidden Markov Model (HMM) without programming skills or understanding the algorithms in detail? In this concise tutorial, we present the HMM through the 2 general questions it was initially developed to answer and describe its elements. The HMM elements include variables, hidden and observed parameters, the vector of initial probabilities, and the transition and emission probability matrices. Then, we suggest a set of ordered steps, for modeling the variables and illustrate them with a simple exercise of modeling and predicting transmembrane segments in a protein sequence. Finally, we show how to interpret the results of the algorithms for this particular problem. To guide the process of information input and explicit solution of the basic HMM algorithms that answer the HMM questions posed, we developed an educational webserver called HMMTeacher. Additional solved HMM modeling exercises can be found in the user’s manual and answers to frequently asked questions. HMMTeacher is available at https://hmmteacher.mobilomics.org, mirrored at https://hmmteacher1.mobilomics.org. A repository with the code of the tool and the webpage is available at https://gitlab.com/kmilo.f/hmmteacher.

## Introduction

Hidden Markov Model (HMM) is a general modeling technique suited to represent a sequence of hidden features in time or space, in which each hidden feature causes or emits an observation [1]. In practice, given a model, an observed sequence is the input, for which the most basic HMM either calculates its probability or outputs a prediction of the most probable sequence of hidden features in it. Two known application examples are as follows: (1) the discovery of the most probable sequence of hidden functional motifs, in a sequence of observed nucleotides, or a gene; and (2) the most probable sequence of hidden protein structural features, like α-helices or β-sheets, in an observed sequence of amino acids, or a protein [2]. The examples, however, are not restricted to computational biology. To find the most probable sequence of letters or words in a sequence of sounds, or speech recognition, was the first problem that inspired HMM development in the 1960s and 1970s [3].

Given a question suitable for HMMs, in terms of observed sequence of symbols and the hidden features we want to discover, the next step is to build the model. However, as the meaning of modeling is broad, we will arbitrarily divide it into “variables modeling” and “parameters estimation.” In an HMM, the variables modeling consists in abstracting the situation to be modeled in terms of observed and hidden variables, and their relationships called parameters. Broadly, this concept refers to a model of computation called (finite) state machine or finite state automaton and is applicable not only to HMMs, but to any graph model [4]. The parameters estimation can be divided into training and validation. Training is the calculation of parameters. It is achieved, usually, through the data processing of an available set of observed sequences with a known mapping of hidden features. This set is called training set. Baum–Welch and Viterbi training are common algorithms for model training [5]. Validation is a quality control process of assessing how general the predictions are. If the predictions are correct only for the training set, the model is said to be overtrained. Cross-validation and simulation are current methods for model validation [6]. Whenever the training set is large enough, part of it is used for training the model, and the rest is used to validate it. Sensitivity and Selectivity, Receiver Operating Characteristics (ROC) charts, and other measures are performance indicators for a model [7].

The focus of this tutorial is on the variables modeling of an HMM. In what follows, we will show the elements of an HMM and a set of practical rules to model in an orderly manner the variables of a situation using HMMs, and answer, through the most basic HMM algorithms Forward and Viterbi, respectively, 2 questions: (1) What is the probability of an observed sequence given a model? and (2) What is the most probable sequence of hidden features in it? Finally, we will interpret the results in an analysis of the parameters under the light of the modeled problem. For this, we will illustrate the rules with a simplified modeling of a known bioinformatic puzzle, the prediction of transmembrane segments in a protein sequence, using a publicly available web server, HMMTeacher, which we developed for educational purposes. Our target audience are beginners in HMM modeling and trainers. Therefore, variations and extensions of HMMs were not included here.

### Elements of an HMM

As previously mentioned, a suitable question to be modeled with an HMM is one in which the inputs and outputs are sequences of observations and hidden features, respectively. The term “sequence” is key here. It is an ordered list of symbols. This order is represented by conditional probabilities between consecutive positions or moments of the sequence, called for generality “states.” These dependency probabilities between consecutive hidden states are called transition probabilities or Markovian dependencies, after the mathematician Andrey Markov (1856–1922). Thus, the name Hidden Markov Model. As a sequence starts with a first symbol, there is no dependency of this symbol with a symbol before it. For this reason, one additional probability vector must be set, the one with the initial or prior probabilities for each hidden symbol of the HMM. As the discussion of Begin and End states is beyond the scope of this review, more information can be found in [1,2].

**Fig 1:**
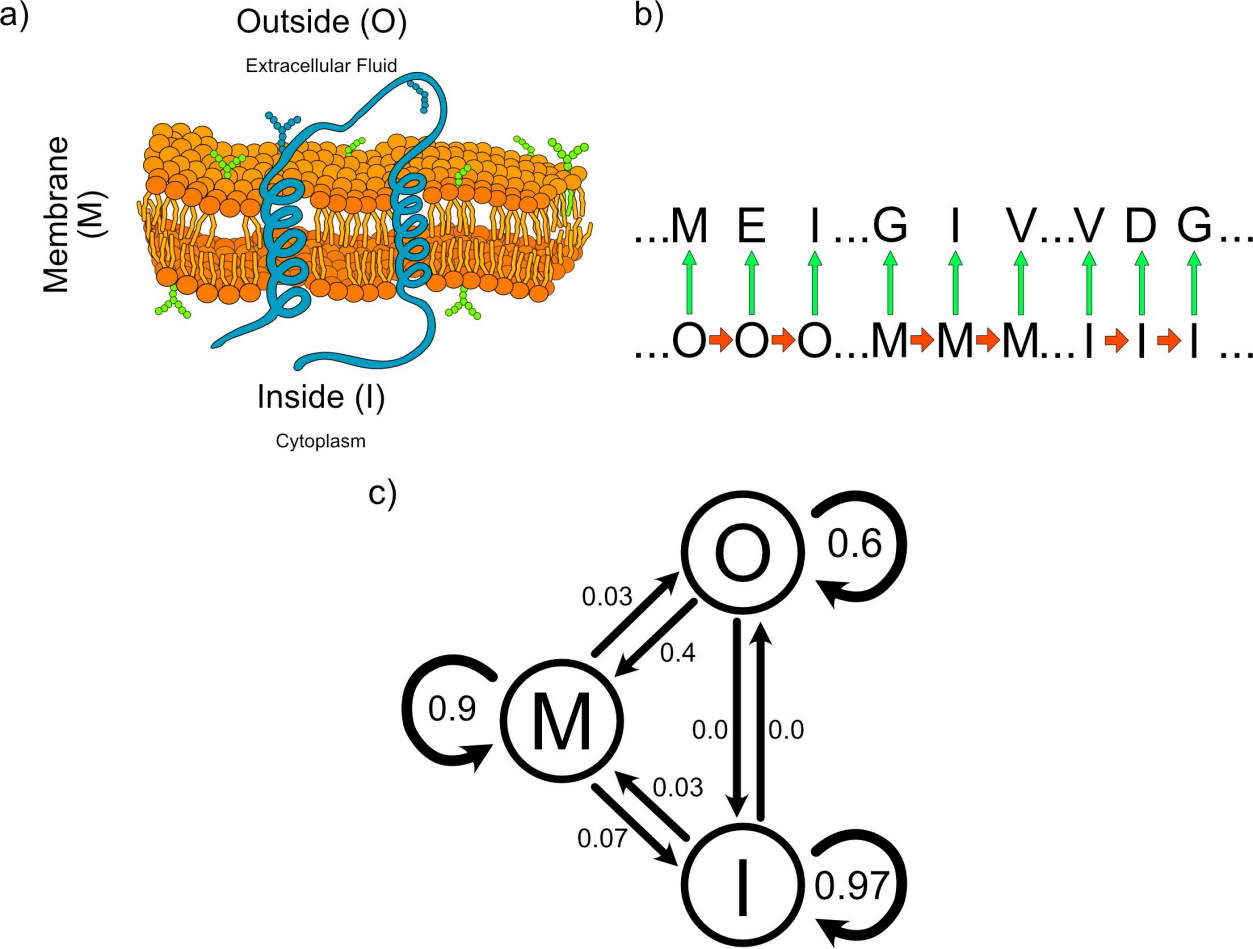
(A) Structural representation of a protein inserted into a membrane with several segments traversing the bilipid layer. (B) Example of an HMM process. The process starts with a hidden state, in this case I (Inside), M (Membrane), or O (Outside), which emits (green arrow) an observed state (an amino acid in 1 letter code). Then, it transitions to the next hidden state (orange arrow), which emits the next observed state, and so on. (C) Graph representation of the transition probability matrix of the HMM. The circle nodes represent the hidden states; the arrows between hidden states are transition probabilities. The emission probabilities were omitted from the graph for simplicity (S5 File). HMM, Hidden Markov Model.

An HMM, then, can be described as a set of hidden symbols connected by Markovian dependencies, which “emit” observed symbols (Fig 1B). The emissions are also conditional probabilities. They represent the relationship between the hidden states and the observations. A directed graph is a structure of nodes and edges that represent an HMM (Fig 1C). The nodes represent the hidden symbols, and the arrows represent the transition probabilities that relate them.

Therefore, the elements of an HMM are a set of observed symbols, a sequence of observed symbols, a set of hidden symbols, a vector of prior probabilities of hidden symbols, a matrix of transition probabilities between hidden symbols, and a matrix of emission probabilities between hidden and observed symbols.

### The modeling steps

Assuming there is already a question suitable for HMM modeling, the basic ordered steps we suggest to designate the elements are as follows:

1. Define the sequence of observed symbols; the query, or input of the HMM.
2. Define the alphabet of observed symbols. These are derived from the observed input just defined.
3. Define the alphabet of hidden symbols, or hidden states. These define attribute(s), which their most probable sequence we seek to know in the query. These symbols define the format of the output of the HMM.
4. Determine the vector of prior probabilities of the hidden states. This is the probability distribution of the first hidden state.
5. Establish the transition probabilities between hidden states.
6. Establish the emission probabilities from a hidden state to an observed state.

### A working example of Hidden Markov Model*ing*

To illustrate these steps, we will develop in a simplistic form a well-known, many times addressed since 1998 [8], bioinformatic problem of the prediction of transmembrane segments in a protein sequence [9,10]. Transmembrane proteins are one of the oldest types of proteins known [11]. After translation, they are embedded into the membrane of the cell (Fig 1A) [12], to serve as channels or transporters, for exchange of molecules with the medium [13], or to serve structural purposes, like cell walls for mechanic rigidity and defense [14], or anchors for organelles [15], or to serve the immune system as presenters of potentially antigenic peptides [16], among other functions [17,18]. To understand their function, it is useful to describe in the protein sequence which subsegments are faced inward, outward, and the segments that will attach the protein molecule to the bilipid layer of the cell.

The novelty in this exercise is doing so in a manner such that anyone without programming skills or background in HMM algorithms can reproduce explicitly the modeling, the development of the algorithms toward the results, and their back translation to the context of the problem, i.e., their interpretation.

In the following, we will apply each proposed abstract rule above. HMMTeacher, an educational tool to develop and solve HMMs, will help the process of modeling by taking the inputs in an orderly manner. There are alternative tools (S4 File). However, HMMTeacher is, in our experience, the most complete, user friendly, and general enough to edit a simple model, solve it explicitly, and have the results ready for interpretation, without needing any familiarity with the terminal or statistical analysis programs like R or Matlab, nor practice in programming.

1. The observed sequence will be a sequence of the protein of which we want to make the transmembrane prediction. In this case, it will be a 100 aa sequence, obtained from the 5-hydroxytryptamine receptor 2A with UniProtKB—P18599:
MEILCEDNTSLSSIPNSLMQVDGDSGLYRNDFNSRDANSSDASNWTIDGENRTNLSFEGYLPPTCLSILHLQEKNWSALLTAVVIILTIAGNILVIMAVS
2. This also defines the set of observed states alphabet, which is the amino acid alphabet or a subset of it. In HMMTeacher, choose the Protein alphabet option. Then, copy and paste the observed sequence into the corresponding box.
3. The alphabet of hidden states. The original problem modeled by Krogh and colleagues, TMHMM [19], had over 150 hidden states. Subsets of hidden states represented different sequence signals, namely, the globular protein that sometimes hangs from the membrane facing the cytosol, the cap signal that represents the amino acids at the extremes of the α-helix at the core of the membrane, the α-helix in the core of the membrane, the loops at the outside of the membrane (non-cytosol), and the final globular protein that is sometimes attached to the outer side of the cell membrane. The more hidden states there are, the more precise is the answer of the HMM. However, as we are looking for developing basic modeling skills, we will naively represent all of this only with 3 states: Inside (I), Membrane (M), and Outside (O). The final output of TMHMM (S2 File) is the most probable sequence of these 3 hidden states, which results from the postprocessing of the Viterbi prediction using the large set of hidden states. In HMMTeacher, we just add the letters in the boxes of “Hidden states” in the Protein central section of the webpage.

The next step is to determine the parameters of the HMM: (4) the vector of prior probabilities of the hidden states; (5) matrix of transition probabilities, representing the frequencies by which the hidden states change in a training set of membrane protein sequences; and (6) the matrix of emission probabilities, representing the frequency composition of the amino acids for each hidden state. For this particular example, we copied the emission probabilities from the TMHMM webpage (S5 File) and invented the transition probabilities so to have a membrane prediction similar to the one from a recently available improved membrane predictor, DeepTMHMM [20]. The input parameters are available at the top of S1 File. The transition probabilities matrix of our HMM can also be represented as a graph (Fig 1C).

In the case the user does not know what numbers to use for filling the matrices, HMMTeacher counts with a “Random” button, which assign them automatically. The idea is to explicitly show that the parameters are the heart of the model and that they can be changed to see how they affect the final prediction. To decide which parameters to change, the interpretation of the probabilities of the matrices is the key. In our case, the vector of prior probabilities means how frequently, after being inserted into the cell bilipid layer, the N-terminal of the protein ends up in the cytosol, in the membrane, and in the outside of the cell. As these are all the possibilities, the probabilities must sum to 1. The transition probability matrix and the emission probability matrix can be interpreted as part of the membrane properties that affect the topology of the protein when it is inserted. Different membrane lipids composition [21] could render different transition and emission matrix probabilities, resulting different protein topologies [22]. The emission probability matrix could model the properties of direct interaction between the membrane with the protein amino acids [23].

**Fig 2:**
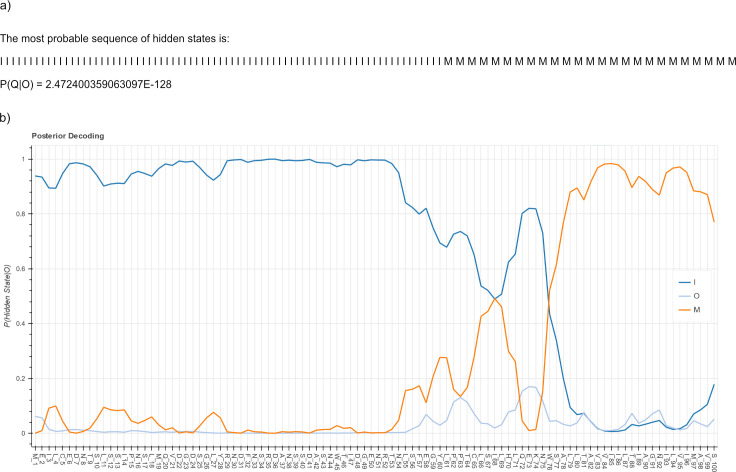
Results of Viterbi and Backward algorithms for the prediction of membrane segments in our example protein sequence. (A) Viterbi prediction of the most probable sequence of hidden states: Inside (I), Membrane (M), and Outside (O), per input protein sequence position. In the HMMTeacher Viterbi output, Q is the sequence of hidden states. The letter O inside the probability is the sequence of observed states. P(Q|O) is the conditional probability of the hidden states given the sequence of observed states. (B) Posterior decoding chart derived from running the Backward algorithm in all observed protein sequence positions for all hidden states.

After defining the parameters of the HMM, the model is ready to be interrogated. In the next step, the user defines which questions to answer. The question of predicting which segments are inside, which are within the membrane, and which are outside can be expressed generally as “The most probable sequence of hidden states that emit the query protein sequence.” The HMM algorithm called Viterbi is the one to answer this question. However, HMMTeacher can answer 2 additional questions for which there are 2 additional algorithms, Forward and Backward. The first algorithm answers the question “What is the probability of the observed sequence, under the model?” The second one, not previously mentioned, answers the question “What is the probability that a particular hidden state, “*i*,” emitted the observed state at a particular sequence position, “*j*”?” If we answer the Backward question for every observed position, and each hidden state, we end up with a chart of probabilities of emission per hidden state, in Y axis, and the observed sequence positions, in X axis. This chart is called “Posterior decoding” (Fig 2B) [5].

In the final step, HMMTeacher provides the explicit solution for all chosen algorithms. The results page shows the input parameters of the HMM followed by 1 tab per algorithm. Each tab shows the recursion formulas adapted from [5,24], and the calculations step-by-step. In the end of each tab, the result. The Forward algorithm tab shows the probability of the observed sequence; Viterbi tab shows the most probable sequence of hidden states; and Backward tab shows the Posterior decoding chart. Most of the time, the sequence of hidden states with the highest probabilities in the Posterior decoding chart agrees with Viterbi prediction (S1 File).

### How to interpret the results?

The probability of an observed sequence given a model is a measure of how well the query information fits the HMM of the problem. However, probability values become meaningful only when compared with other probabilities. Therefore, without running the HMM for different queries, or the same observed sequence with different parameters, the result from Forward algorithm has no initial meaning. For long observed sequences, the Forward will render very low values (in the order of 10^−127^, in this case). The results from Viterbi and Posterior decoding are almost self-explanatory. In our case (Fig 2B), we have a clear prediction of a membrane segment from protein position 76 onwards. Viterbi result agrees with this prediction. What about before this position? The chart depicts an inside (I) prediction with high probability, which tends to fall coming closer to the 53rd position, in favor to the membrane (M) probability. TMHMM and DeepTMHMM smoothed the results averaging the probabilities over a sliding window of positions, rendering a slightly lower probability than we have in our example, but apparently more stable for the protein to be in the cytosol than the outside of the cell (S2 and S3 Files).

This made-up, arguably oversimplified, and overtrained example imitates the membrane prediction of TMHMM and DeepTMHMM. Notice that the transition probability parameters, showed in Fig 1C, are plausible: The transitions between I and O hidden states are both null in probability, as these transitions are not physically possible in the cell. On the other hand, the remaining transition probabilities reflect the expected frequency of amino acids between hidden states. In particular, the transition probabilities between each state and itself are close to 1, for I and M, which is the situation we are modeling.

## Conclusions

When modeling an HMM, the hidden variables are the ones that represent the elements of the answer we are looking for. The observed sequence is the query, and the parameters are the information of how the hidden states are related between each other, and with the observed states. Finally, the understanding of the parameters of an HMM and the consequences they convey in the prediction of (hidden) attributes is a powerful tool for understanding the biology of proteins. HMMTeacher is a calculator that facilitates this process.

## Afterthought

After more than 15 years teaching Markov Chains and HMMs as the last of 3 units in a semester course of “Mathematical Models in Biological Systems” for Bioinformatics Engineer students, we have accepted that teaching the math needed, the modeling, the algorithms in detail, the programming of the algorithms, their solution, and the interpretation of the results within the biological context of the initial problem takes more than 3 or 4 weeks in an undergraduate classroom. HMMTeacher saves teaching time by apportioning the process of modeling and solving the model, between the user and the machine. Each one does what each does best. We humans do the thinking and apply ingenuity: problem modeling and results interpretation. The server performs all the calculations in between. HMMTeacher allows to teach HMM basics and practice the modeling in classes with the help of the HMMTeacher manual’s exercises, within 2 weeks.

We have been using HMMTeacher for at least 3 years. Before that, a couple of surveys showed us that 1 year after the course in which only the HMM theory and algorithms were presented, the students were just familiar with the HMM terminology. Today, the students become enabled at least mechanically if not conceptually to the process of HMModel*ing*. They can actually invent new problems and solve them with HMMTeacher. This is not ideal, since learning the algorithms and their programming is also very important. However, for the ones who really want to learn the algorithms, HMMTeacher shows their solution step-by-step. This is an improvement in bioinformatics education.

## Supporting information

S1 File HMMTeacher report - 5H2A_CRIGR. (PDF)

S2 File TMHMM report - 5H2A_CRIGR. (PDF)

S3 File DeepTMHMM report– 5H2A_CRIGR. (PDF)

S4 File List of software and packages for Hidden Markov Modeling. (XLSX)

S5 File Matrix of emission probabilities of the example. (XLSX)
