# Supplementary material for: Hidden Markov Modeling with HMMTeacher
Source: PLoS Comput Biol. 2022 Feb 10;18(2):e1009703. doi: 10.1371/journal.pcbi.1009703 (PMC8830650; doi:10.1371/journal.pcbi.1009703)

TMHMM result

```
# 5H2A_CRIGR Length: 100
# 5H2A_CRIGR Number of predicted TMHs:  1
# 5H2A_CRIGR Exp number of AAs in TMHs: 22.45732
# 5H2A_CRIGR Exp number, first 60 AAs:  0.01081
# 5H2A_CRIGR Total prob of N-in:        0.70216
5H2A_CRIGR      TMHMM2.0        inside       1    75
5H2A_CRIGR      TMHMM2.0        TMhelix     76    98
5H2A_CRIGR      TMHMM2.0        outside     99   100
```

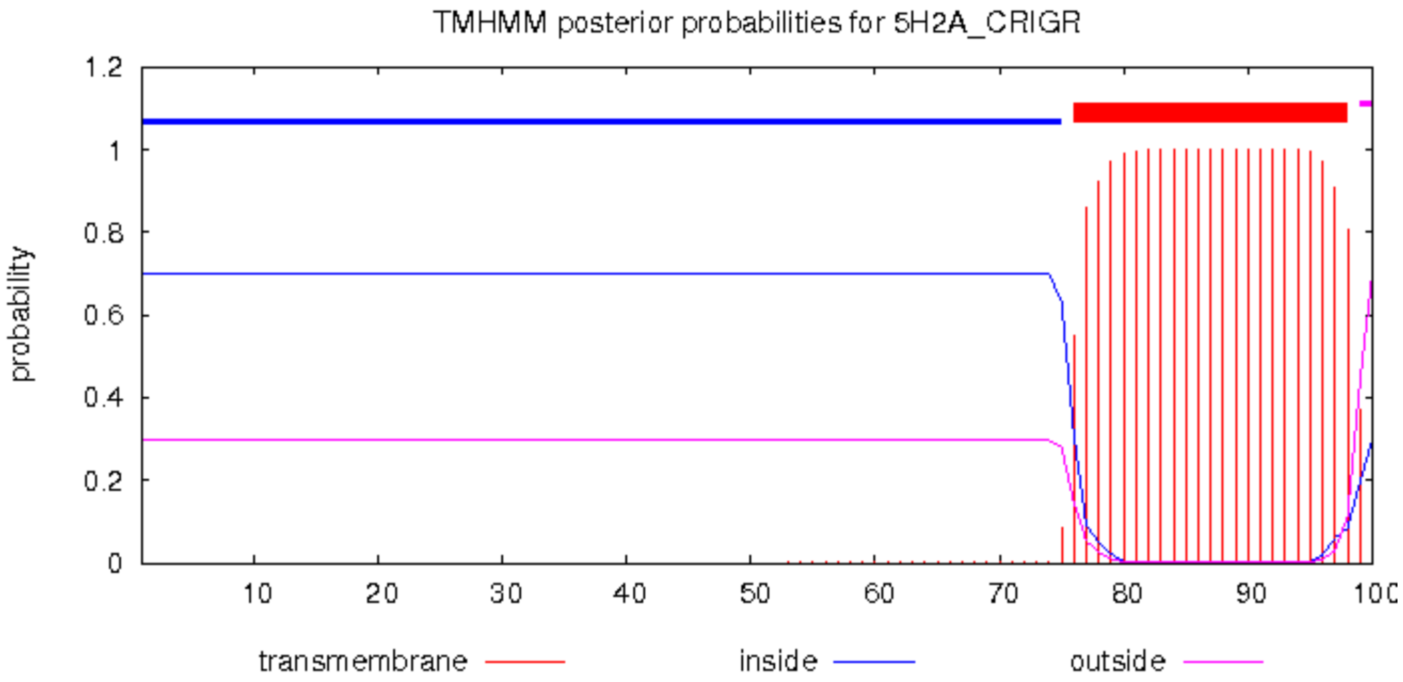

Supplement: S2 File — (PDF) [file pcbi.1009703.s002.pdf]
