# Supplementary material for: Hidden Markov Modeling with HMMTeacher
Source: PLoS Comput Biol. 2022 Feb 10;18(2):e1009703. doi: 10.1371/journal.pcbi.1009703 (PMC8830650; doi:10.1371/journal.pcbi.1009703)

DeepTMHMM - Result for 5H2A_CRIGR

5H2A_CRIGR Length: 100
5H2A_CRIGR Number of predicted TMRs: 1

5H2A_CRIGR	DeepTMHMM	outside	1	78
5H2A_CRIGR	DeepTMHMM	TMhelix	78	100
5H2A_CRIGR	DeepTMHMM	inside 100	100	

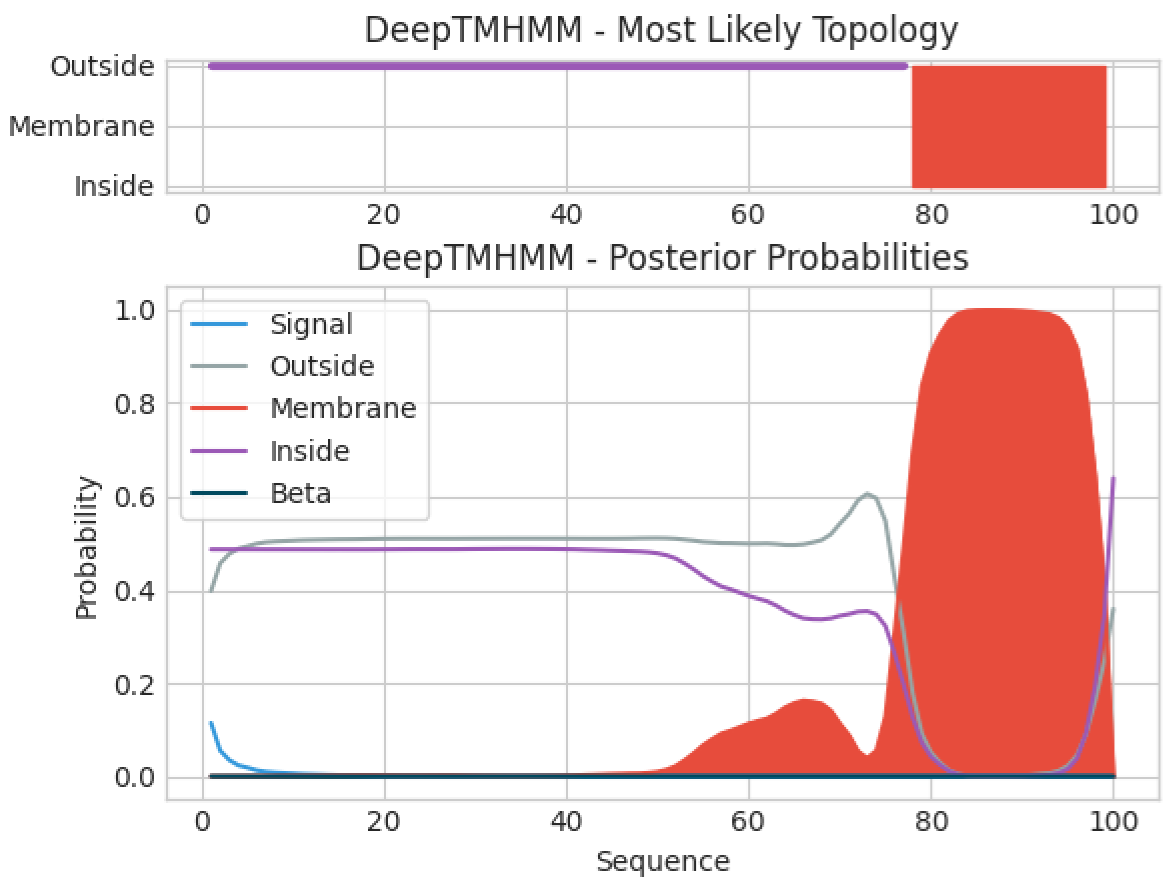

Supplement: S3 File — (PDF) [file pcbi.1009703.s003.pdf]
