# Supplementary material for: Hidden Markov Modeling with HMMTeacher
Source: PLoS Comput Biol. 2022 Feb 10;18(2):e1009703. doi: 10.1371/journal.pcbi.1009703 (PMC8830650; doi:10.1371/journal.pcbi.1009703)
Supplement: S1 File — (PDF) [file pcbi.1009703.s001.pdf]

HMM Teacher results

Supplementary File 1 - Hidden Markov Modeling with HMMTeacher.
Modified from web version for publication purposes.

Observed sequence

MEILCEDNTSLSSIPNSLMQVDGDS
GLYRNDFNSRDANSSDASNWTIDGE
NRTNLSFEGYLPPTCLSILHLQEKNW
SALLTAVVIILTIAGNILVIMAVS

Alphabet

- ARNDCEQGHILKMFPSTWYV

Hidden States

- I
- O
- M

Selected algorithms

1. Forward
2. Viterbi
3. Backward

Transition matrix

	I	O	M
**I**	0.970000	0.000000	0.030000
**O**	0.000000	0.600000	0.400000
**M**	0.070000	0.030000	0.900000

Emission matrix

	A	R	N	D	C	E	Q	G	H	I	L	K	M	F	P	S	T
**I**	0.069010	0.115180	0.058770	0.041430	0.020190	0.046210	0.050920	0.055910	0.022530	0.042940	0.051710	0.090420	0.042100	0.030040	0.039530	0.064090	0.065[illegible]
**O**	0.070520	0.072320	0.042710	0.039900	0.015970	0.054090	0.039620	0.072390	0.021290	0.042570	0.103300	0.056380	0.040800	0.055770	0.058680	0.069270	0.055[illegible]
**M**	0.095360	0.008520	0.018790	0.000660	0.013280	0.004570	0.006870	0.068980	0.007820	0.119350	0.194290	0.003980	0.025680	0.091480	0.015570	0.049950	0.022[illegible]

Prior Probabilities

- **I** : 0.9
- **O** : 0.1
- **M** : 0.0

Algorithm Information

Forward

Initialization

$$\alpha(t=1,i) = \pi_i \bullet e_{S_i}(O_{t=1})$$

T: 1

i: 1
α(1,1) = Π_1 * emi(I,M)
α(1,1) = 0.9 * 0.0421 = 0.03789

i: 2
α(1,2) = Π_2 * emi(O,M)
α(1,2) = 0.1 * 0.0408 = 0.00408

i: 3
α(1,3) = Π_3 * emi(M,M)
α(1,3) = 0.0 * 0.02568 = 0.0

Recursion

$$N$$

$$\alpha(t,i) = e_{q_t=S_i}(O_t) \cdot \sum_{j=1} \alpha(t-1,j) \cdot a_{ji} \quad \forall i$$

T: 2

i: 1
α(2,1) = emi(1,2) * Σ[[α((2-1),1) * trans(1,1)] + [α((2-1),2) * trans(2,1)] + [α((2-1),3) * trans(3,1)]]
α(2,1) = 0.04621 * [0.03789 * 0.97] + [0.00408 * 0.0] + [0.0 * 0.07] = 0.0016983699929999999

i: 2
α(2,2) = emi(2,2) * Σ[[α((2-1),1) * trans(1,2)] + [α((2-1),2) * trans(2,2)] + [α((2-1),3) * trans(3,2)]]
α(2,2) = 0.05409 * [0.03789 * 0.0] + [0.00408 * 0.6] + [0.0 * 0.03] = 0.00013241232000000001

i: 3
α(2,3) = emi(3,2) * Σ[[α((2-1),1) * trans(1,3)] + [α((2-1),2) * trans(2,3)] + [α((2-1),3) * trans(3,3)]]
α(2,3) = 0.00457 * [0.03789 * 0.03] + [0.00408 * 0.4] + [0.0 * 0.9] = 1.2652959000000002e-05

T: 3

i: 1
α(3,1) = emi(1,3) * Σ[[α((3-1),1) * trans(1,1)] + [α((3-1),2) * trans(2,1)] + [α((3-1),3) * trans(3,1)]]
α(3,1) = 0.04294 * [0.0016983699929999999 * 0.97] + [0.00013241232000000001 * 0.0] + [1.2652959000000002e-05 * 0.07] = 7.077819953859958e-05

i: 2
α(3,2) = emi(2,3) * Σ[[α((3-1),1) * trans(1,2)] + [α((3-1),2) * trans(2,2)] + [α((3-1),3) * trans(3,2)]]
α(3,2) = 0.04257 * [0.0016983699929999999 * 0.0] + [0.00013241232000000001 * 0.6] + [1.2652959000000002e-05 * 0.03] = 3.3982345713789e-06

i: 3
α(3,3) = emi(3,3) * Σ[[α((3-1),1) * trans(1,3)] + [α((3-1),2) * trans(2,3)] + [α((3-1),3) * trans(3,3)]]
α(3,3) = 0.11935 * [0.0016983699929999999 * 0.03] + [0.00013241232000000001 * 0.4] + [1.2652959000000002e-05 * 0.9] = 1.37614955077215e-05

T: 4

i: 1
α(4,1) = emi(1,4) * Σ[[α((4-1),1) * trans(1,1)] + [α((4-1),2) * trans(2,1)] + [α((4-1),3) * trans(3,1)]]
α(4,1) = 0.05171 * [7.077819953859958e-05 * 0.97] + [3.3982345713789e-06 * 0.0] + [1.37614955077215e-05 * 0.07] = 3.5999549624860548e-06

i: 2
α(4,2) = emi(2,4) * Σ[[α((4-1),1) * trans(1,2)] + [α((4-1),2) * trans(2,2)] + [α((4-1),3) * trans(3,2)]]
α(4,2) = 0.1033 * [7.077819953859958e-05 * 0.0] + [3.3982345713789e-06 * 0.6] + [1.37614955077215e-05 * 0.03] = 2.5326945331249313e-07

i: 3
α(4,3) = emi(3,4) * Σ[[α((4-1),1) * trans(1,3)] + [α((4-1),2) * trans(2,3)] + [α((4-1),3) * trans(3,3)]]
α(4,3) = 0.19429 * [7.077819953859958e-05 * 0.03] + [3.3982345713789e-06 * 0.4] + [1.37614955077215e-05 * 0.9] = 3.082990955575607e-06

T: 5

i: 1
α(5,1) = emi(1,5) * Σ[[α((5-1),1) * trans(1,1)] + [α((5-1),2) * trans(2,1)] + [α((5-1),3) * trans(3,1)]]
α(5,1) = 0.02019 * [3.5999549624860548e-06 * 0.97] + [2.5326945331249313e-07 * 0.0] + [3.082990955575607e-06 * 0.07] = 7.485978908933065e-08

i: 2
α(5,2) = emi(2,5) * Σ[[α((5-1),1) * trans(1,2)] + [α((5-1),2) * trans(2,2)] + [α((5-1),3) * trans(3,2)]]
α(5,2) = 0.01597 * [3.5999549624860548e-06 * 0.0] + [2.5326945331249313e-07 * 0.6] + [3.082990955575607e-06 * 0.03] = 3.903888868456583e-09

i: 3
α(5,3) = emi(3,5) * Σ[[α((5-1),1) * trans(1,3)] + [α((5-1),2) * trans(2,3)] + [α((5-1),3) * trans(3,3)]]
α(5,3) = 0.01328 * [3.5999549624860548e-06 * 0.03] + [2.5326945331249313e-07 * 0.4] + [3.082990955575607e-06 * 0.9] = 3.962749729409006e-08

T: 6

i: 1
α(6,1) = emi(1,6) * Σ[[α((6-1),1) * trans(1,1)] + [α((6-1),2) * trans(2,1)] + [α((6-1),3) * trans(3,1)]]
α(6,1) = 0.04621 * [7.485978908933065e-08 * 0.97] + [3.903888868456583e-09 * 0.0] + [3.962749729409006e-08 * 0.07] = 3.4836757937006234e-09

i: 2
α(6,2) = emi(2,6) * Σ[[α((6-1),1) * trans(1,2)] + [α((6-1),2) * trans(2,2)] + [α((6-1),3) * trans(3,2)]]
α(6,2) = 0.05409 * [7.485978908933065e-08 * 0.0] + [3.903888868456583e-09 * 0.6] + [3.962749729409006e-08 * 0.03] = 1.9100034919600988e-10

i: 3
α(6,3) = emi(3,6) * Σ[[α((6-1),1) * trans(1,3)] + [α((6-1),2) * trans(2,3)] + [α((6-1),3) * trans(3,3)]]
α(6,3) = 0.00457 * [7.485978908933065e-08 * 0.03] + [3.903888868456583e-09 * 0.4] + [3.962749729409006e-08 * 0.9] = 1.803874823062783e-10

T: 7

i: 1
α(7,1) = emi(1,7) * Σ[[α((7-1),1) * trans(1,1)] + [α((7-1),2) * trans(2,1)] + [α((7-1),3) * trans(3,1)]]
α(7,1) = 0.04143 * [3.4836757937006234e-09 * 0.97] + [1.9100034919600988e-10 * 0.0] + [1.803874823062783e-10 * 0.07] = 1.4052196922646276e-10

i: 2

α(7,2) = emi(2,7) * Σ[[α((7-1),1) * trans(1,2)] + [α((7-1),2) * trans(2,2)] + [α((7-1),3) * trans(3,2)]]
α(7,2) = 0.0399 * [3.4836757937006234e-09 * 0.0] + [1.9100034919600988e-10 * 0.6] + [1.803874823062783e-10 * 0.03] = 4.7884721760730905e-12

i: 3
α(7,3) = emi(3,7) * Σ[[α((7-1),1) * trans(1,3)] + [α((7-1),2) * trans(2,3)] + [α((7-1),3) * trans(3,3)]]
α(7,3) = 0.00066 * [3.4836757937006234e-09 * 0.03] + [1.9100034919600988e-10 * 0.4] + [1.803874823062783e-10 * 0.9] = 2.2655103739294827e-13

T: 8

i: 1
α(8,1) = emi(1,8) * Σ[[α((8-1),1) * trans(1,1)] + [α((8-1),2) * trans(2,1)] + [α((8-1),3) * trans(3,1)]]
α(8,1) = 0.05877 * [1.4052196922646276e-10 * 0.97] + [4.7884721760730905e-12 * 0.0] + [2.2655103739294827e-13 * 0.07] = 8.01165385580877e-12

i: 2
α(8,2) = emi(2,8) * Σ[[α((8-1),1) * trans(1,2)] + [α((8-1),2) * trans(2,2)] + [α((8-1),3) * trans(3,2)]]
α(8,2) = 0.04271 * [1.4052196922646276e-10 * 0.0] + [4.7884721760730905e-12 * 0.6] + [2.2655103739294827e-13 * 0.03] = 1.2299966782826058e-13

i: 3
α(8,3) = emi(3,8) * Σ[[α((8-1),1) * trans(1,3)] + [α((8-1),2) * trans(2,3)] + [α((8-1),3) * trans(3,3)]]
α(8,3) = 0.01879 * [1.4052196922646276e-10 * 0.03] + [4.7884721760730905e-12 * 0.4] + [2.2655103739294827e-13 * 0.9] = 1.1903359552167456e-13

T: 9

i: 1
α(9,1) = emi(1,9) * Σ[[α((9-1),1) * trans(1,1)] + [α((9-1),2) * trans(2,1)] + [α((9-1),3) * trans(3,1)]]
α(9,1) = 0.06521 * [8.01165385580877e-12 * 0.97] + [1.2299966782826058e-13 * 0.0] + [1.1903359552167456e-13 * 0.07] = 5.07310102152649e-13

i: 2
α(9,2) = emi(2,9) * Σ[[α((9-1),1) * trans(1,2)] + [α((9-1),2) * trans(2,2)] + [α((9-1),3) * trans(3,2)]]
α(9,2) = 0.05586 * [8.01165385580877e-12 * 0.0] + [1.2299966782826058e-13 * 0.6] + [1.1903359552167456e-13 * 0.03] = 4.321933366307204e-15

i: 3
α(9,3) = emi(3,9) * Σ[[α((9-1),1) * trans(1,3)] + [α((9-1),2) * trans(2,3)] + [α((9-1),3) * trans(3,3)]]
α(9,3) = 0.02246 * [8.01165385580877e-12 * 0.03] + [1.2299966782826058e-13 * 0.4] + [1.1903359552167456e-13 * 0.9] = 8.909426483688172e-15

T: 10

i: 1
α(10,1) = emi(1,10) * Σ[[α((10-1),1) * trans(1,1)] + [α((10-1),2) * trans(2,1)] + [α((10-1),3) * trans(3,1)]]
α(10,1) = 0.06409 * [5.07310102152649e-13 * 0.97] + [4.321933366307204e-15 * 0.0] + [8.909426483688172e-15 * 0.07] = 3.157806967358814e-14

i: 2
α(10,2) = emi(2,10) * Σ[[α((10-1),1) * trans(1,2)] + [α((10-1),2) * trans(2,2)] + [α((10-1),3) * trans(3,2)]]
α(10,2) = 0.06927 * [5.07310102152649e-13 * 0.0] + [4.321933366307204e-15 * 0.6] + [8.909426483688172e-15 * 0.03] = 1.981428737462124e-16

i: 3
α(10,3) = emi(3,10) * Σ[[α((10-1),1) * trans(1,3)] + [α((10-1),2) * trans(2,3)] + [α((10-1),3) * trans(3,3)]]
α(10,3) = 0.04995 * [5.07310102152649e-13 * 0.03] + [4.321933366307204e-15 * 0.4] + [8.909426483688172e-15 * 0.9] = 1.2470796843087644e-15

T: 11

i: 1
α(11,1) = emi(1,11) * Σ[[α((11-1),1) * trans(1,1)] + [α((11-1),2) * trans(2,1)] + [α((11-1),3) * trans(3,1)]]
α(11,1) = 0.05171 * [3.157806967358814e-14 * 0.97] + [1.981428737462124e-16 * 0.0] + [1.2470796843087644e-15 * 0.07] = 1.5884289776698978e-15

i: 2
α(11,2) = emi(2,11) * Σ[[α((11-1),1) * trans(1,2)] + [α((11-1),2) * trans(2,2)] + [α((11-1),3) * trans(3,2)]]
α(11,2) = 0.1033 * [3.157806967358814e-14 * 0.0] + [1.981428737462124e-16 * 0.6] + [1.2470796843087644e-15 * 0.03] = 1.6145595256463108e-17

i: 3
α(11,3) = emi(3,11) * Σ[[α((11-1),1) * trans(1,3)] + [α((11-1),2) * trans(2,3)] + [α((11-1),3) * trans(3,3)]]
α(11,3) = 0.19429 * [3.157806967358814e-14 * 0.03] + [1.981428737462124e-16 * 0.4] + [1.2470796843087644e-15 * 0.9] = 4.1752356696041867e-16

T: 12

i: 1
α(12,1) = emi(1,12) * Σ[[α((12-1),1) * trans(1,1)] + [α((12-1),2) * trans(2,1)] + [α((12-1),3) * trans(3,1)]]
α(12,1) = 0.06409 * [1.5884289776698978e-15 * 0.97] + [1.6145595256463108e-17 * 0.0] + [4.1752356696041867e-16 * 0.07] = 1.0062147676195236e-16

i: 2
α(12,2) = emi(2,12) * Σ[[α((12-1),1) * trans(1,2)] + [α((12-1),2) * trans(2,2)] + [α((12-1),3) * trans(3,2)]]
α(12,2) = 0.06927 * [1.5884289776698978e-15 * 0.0] + [1.6145595256463108e-17 * 0.6] + [4.1752356696041867e-16 * 0.03] = 1.5386989545495656e-18

i: 3
α(12,3) = emi(3,12) * Σ[[α((12-1),1) * trans(1,3)] + [α((12-1),2) * trans(2,3)] + [α((12-1),3) * trans(3,3)]]
α(12,3) = 0.04995 * [1.5884289776698978e-15 * 0.03] + [1.6145595256463108e-17 * 0.4] + [4.1752356696041867e-16 * 0.9] = 2.1472621768968096e-17

T: 13

i: 1
α(13,1) = emi(1,13) * Σ[[α((13-1),1) * trans(1,1)] + [α((13-1),2) * trans(2,1)] + [α((13-1),3) * trans(3,1)]]
α(13,1) = 0.06409 * [1.0062147676195236e-16 * 0.97] + [1.5386989545495656e-18 * 0.0] + [2.1472621768968096e-17 * 0.07] = 6.3516981553454416e-18

i: 2
α(13,2) = emi(2,13) * Σ[[α((13-1),1) * trans(1,2)] + [α((13-1),2) * trans(2,2)] + [α((13-1),3) * trans(3,2)]]
α(13,2) = 0.06927 * [1.0062147676195236e-16 * 0.0] + [1.5386989545495656e-18 * 0.6] + [2.1472621768968096e-17 * 0.03] = 1.0857366124708165e-19

i: 3
α(13,3) = emi(3,13) * Σ[[α((13-1),1) * trans(1,3)] + [α((13-1),2) * trans(2,3)] + [α((13-1),3) * trans(3,3)]]
α(13,3) = 0.04995 * [1.0062147676195236e-16 * 0.03] + [1.5386989545495656e-18 * 0.4] + [2.1472621768968096e-17 * 0.9] = 1.1468261996636467e-18

T: 14

i: 1
α(14,1) = emi(1,14) * Σ[[α((14-1),1) * trans(1,1)] + [α((14-1),2) * trans(2,1)] + [α((14-1),3) * trans(3,1)]]
α(14,1) = 0.04294 * [6.3516981553454416e-18 * 0.97] + [1.0857366124708165e-19 * 0.0] + [1.1468261996636467e-18 * 0.07] = 2.6800679141776625e-19

i: 2
α(14,2) = emi(2,14) * Σ[[α((14-1),1) * trans(1,2)] + [α((14-1),2) * trans(2,2)] + [α((14-1),3) * trans(3,2)]]
α(14,2) = 0.04257 * [6.3516981553454416e-18 * 0.0] + [1.0857366124708165e-19 * 0.6] + [1.1468261996636467e-18 * 0.03] = 4.237800195163402e-21

i: 3
α(14,3) = emi(3,14) * Σ[[α((14-1),1) * trans(1,3)] + [α((14-1),2) * trans(2,3)] + [α((14-1),3) * trans(3,3)]]
α(14,3) = 0.11935 * [6.3516981553454416e-18 * 0.03] + [1.0857366124708165e-19 * 0.4] + [1.1468261996636467e-18 * 0.9] = 1.5111189807002064e-19

T: 15

i: 1
α(15,1) = emi(1,15) * Σ[[α((15-1),1) * trans(1,1)] + [α((15-1),2) * trans(2,1)] + [α((15-1),3) * trans(3,1)]]
α(15,1) = 0.03953 * [2.6800679141776625e-19 * 0.97] + [4.237800195163402e-21 * 0.0] + [1.5111189807002064e-19 * 0.07] = 1.0694620943951526e-20

i: 2
α(15,2) = emi(2,15) * Σ[[α((15-1),1) * trans(1,2)] + [α((15-1),2) * trans(2,2)] + [α((15-1),3) * trans(3,2)]]
α(15,2) = 0.05868 * [2.6800679141776625e-19 * 0.0] + [4.237800195163402e-21 * 0.6] + [1.5111189807002064e-19 * 0.03] = 4.1522185463377745e-22

i: 3
α(15,3) = emi(3,15) * Σ[[α((15-1),1) * trans(1,3)] + [α((15-1),2) * trans(2,3)] + [α((15-1),3) * trans(3,3)]]
α(15,3) = 0.01557 * [2.6800679141776625e-19 * 0.03] + [4.237800195163402e-21 * 0.4] + [1.5111189807002064e-19 * 0.9] = 2.2691100195419158e-21

T: 16

i: 1
α(16,1) = emi(1,16) * Σ[[α((16-1),1) * trans(1,1)] + [α((16-1),2) * trans(2,1)] + [α((16-1),3) * trans(3,1)]]
α(16,1) = 0.05877 * [1.0694620943951526e-20 * 0.97] + [4.1522185463377745e-22 * 0.0] + [2.2691100195419158e-21 * 0.07] = 6.190020783991438e-22

i: 2
α(16,2) = emi(2,16) * Σ[[α((16-1),1) * trans(1,2)] + [α((16-1),2) * trans(2,2)] + [α((16-1),3) * trans(3,2)]]
α(16,2) = 0.04271 * [1.0694620943951526e-20 * 0.0] + [4.1522185463377745e-22 * 0.6] + [2.2691100195419158e-21 * 0.03] = 1.3547885914884237e-23

i: 3
α(16,3) = emi(3,16) * Σ[[α((16-1),1) * trans(1,3)] + [α((16-1),2) * trans(2,3)] + [α((16-1),3) * trans(3,3)]]
α(16,3) = 0.01879 * [1.0694620943951526e-20 * 0.03] + [4.1522185463377745e-22 * 0.4] + [2.2691100195419158e-21 * 0.9] = 4.7522284826006294e-23

T: 17

i: 1
α(17,1) = emi(1,17) * Σ[[α((17-1),1) * trans(1,1)] + [α((17-1),2) * trans(2,1)] + [α((17-1),3) * trans(3,1)]]
α(17,1) = 0.06409 * [6.190020783991438e-22 * 0.97] + [1.3547885914884237e-23 * 0.0] + [4.7522284826006294e-23 * 0.07] = 3.8694887134878003e-23

i: 2
α(17,2) = emi(2,17) * Σ[[α((17-1),1) * trans(1,2)] + [α((17-1),2) * trans(2,2)] + [α((17-1),3) * trans(3,2)]]
α(17,2) = 0.06927 * [6.190020783991438e-22 * 0.0] + [1.3547885914884237e-23 * 0.6] + [4.7522284826006294e-23 * 0.03] = 6.618332944913422e-25

i: 3
α(17,3) = emi(3,17) * Σ[[α((17-1),1) * trans(1,3)] + [α((17-1),2) * trans(2,3)] + [α((17-1),3) * trans(3,3)]]
α(17,3) = 0.04995 * [6.190020783991438e-22 * 0.03] + [1.3547885914884237e-23 * 0.4] + [4.7522284826006294e-23 * 0.9] = 3.334625689413617e-24

T: 18

i: 1
α(18,1) = emi(1,18) * Σ[[α((18-1),1) * trans(1,1)] + [α((18-1),2) * trans(2,1)] + [α((18-1),3) * trans(3,1)]]
α(18,1) = 0.05171 * [3.8694887134878003e-23 * 0.97] + [6.618332944913422e-25 * 0.0] + [3.334625689413617e-24 * 0.07] = 1.9529555799401756e-24

i: 2
α(18,2) = emi(2,18) * Σ[[α((18-1),1) * trans(1,2)] + [α((18-1),2) * trans(2,2)] + [α((18-1),3) * trans(3,2)]]
α(18,2) = 0.1033 * [3.8694887134878003e-23 * 0.0] + [6.618332944913422e-25 * 0.6] + [3.334625689413617e-24 * 0.03] = 5.135443260406619e-26

i: 3
α(18,3) = emi(3,18) * Σ[[α((18-1),1) * trans(1,3)] + [α((18-1),2) * trans(2,3)] + [α((18-1),3) * trans(3,3)]]
α(18,3) = 0.19429 * [3.8694887134878003e-23 * 0.03] + [6.618332944913422e-25 * 0.4] + [3.334625689413617e-24 * 0.9] = 8.60071907634307e-25

T: 19

i: 1
α(19,1) = emi(1,19) * Σ[[α((19-1),1) * trans(1,1)] + [α((19-1),2) * trans(2,1)] + [α((19-1),3) * trans(3,1)]]
α(19,1) = 0.0421 * [1.9529555799401756e-24 * 0.97] + [5.135443260406619e-26 * 0.0] + [8.60071907634307e-25 * 0.07] = 8.228747892981525e-26

i: 2
α(19,2) = emi(2,19) * Σ[[α((19-1),1) * trans(1,2)] + [α((19-1),2) * trans(2,2)] + [α((19-1),3) * trans(3,2)]]
α(19,2) = 0.0408 * [1.9529555799401756e-24 * 0.0] + [5.135443260406619e-26 * 0.6] + [8.60071907634307e-25 * 0.03] = 2.3098845250919322e-27

i: 3
α(19,3) = emi(3,19) * Σ[[α((19-1),1) * trans(1,3)] + [α((19-1),2) * trans(2,3)] + [α((19-1),3) * trans(3,3)]]
α(19,3) = 0.02568 * [1.9529555799401756e-24 * 0.03] + [5.135443260406619e-26 * 0.4] + [8.60071907634307e-25 * 0.9] = 2.1910051639738986e-26

T: 20

i: 1
α(20,1) = emi(1,20) * Σ[[α((20-1),1) * trans(1,1)] + [α((20-1),2) * trans(2,1)] + [α((20-1),3) * trans(3,1)]]
α(20,1) = 0.05092 * [8.228747892981525e-26 * 0.97] + [2.3098845250919322e-27 * 0.0] + [2.1910051639738986e-26 * 0.07] = 4.1424722623576926e-27

i: 2
α(20,2) = emi(2,20) * Σ[[α((20-1),1) * trans(1,2)] + [α((20-1),2) * trans(2,2)] + [α((20-1),3) * trans(3,2)]]
α(20,2) = 0.03962 * [8.228747892981525e-26 * 0.0] + [2.3098845250919322e-27 * 0.6] + [2.1910051639738986e-26 * 0.03] = 8.095286230947917e-29

i: 3
α(20,3) = emi(3,20) * Σ[[α((20-1),1) * trans(1,3)] + [α((20-1),2) * trans(2,3)] + [α((20-1),3) * trans(3,3)]]
α(20,3) = 0.00687 * [8.228747892981525e-26 * 0.03] + [2.3098845250919322e-27 * 0.4] + [2.1910051639738986e-26 * 0.9] = 1.587768613708937e-28

T: 21

i: 1
α(21,1) = emi(1,21) * Σ[[α((21-1),1) * trans(1,1)] + [α((21-1),2) * trans(2,1)] + [α((21-1),3) * trans(3,1)]]
α(21,1) = 0.04595 * [4.1424722623576926e-27 * 0.97] + [8.095286230947917e-29 * 0.0] + [1.587768613708937e-28 * 0.07] = 1.8514690821627535e-28

i: 2
α(21,2) = emi(2,21) * Σ[[α((21-1),1) * trans(1,2)] + [α((21-1),2) * trans(2,2)] + [α((21-1),3) * trans(3,2)]]
α(21,2) = 0.04801 * [4.1424722623576926e-27 * 0.0] + [8.095286230947917e-29 * 0.6] + [1.587768613708937e-28 * 0.03] = 2.560614465119355e-30

i: 3
α(21,3) = emi(3,21) * Σ[[α((21-1),1) * trans(1,3)] + [α((21-1),2) * trans(2,3)] + [α((21-1),3) * trans(3,3)]]
α(21,3) = 0.14222 * [4.1424722623576926e-27 * 0.03] + [8.095286230947917e-29 * 0.4] + [1.587768613708937e-28 * 0.9] = 4.2602639287388643e-29

T: 22

i: 1
α(22,1) = emi(1,22) * Σ[[α((22-1),1) * trans(1,1)] + [α((22-1),2) * trans(2,1)] + [α((22-1),3) * trans(3,1)]]
α(22,1) = 0.04143 * [1.8514690821627535e-28 * 0.97] + [2.560614465119355e-30 * 0.0] + [4.2602639287388643e-29 * 0.07] = 7.564069229375634e-30

i: 2
α(22,2) = emi(2,22) * Σ[[α((22-1),1) * trans(1,2)] + [α((22-1),2) * trans(2,2)] + [α((22-1),3) * trans(3,2)]]
α(22,2) = 0.0399 * [1.8514690821627535e-28 * 0.0] + [2.560614465119355e-30 * 0.6] + [4.2602639287388643e-29 * 0.03] = 1.1229646952196156e-31

i: 3
α(22,3) = emi(3,22) * Σ[[α((22-1),1) * trans(1,3)] + [α((22-1),2) * trans(2,3)] + [α((22-1),3) * trans(3,3)]]
α(22,3) = 0.00066 * [1.8514690821627535e-28 * 0.03] + [2.560614465119355e-30 * 0.4] + [4.2602639287388643e-29 * 0.9] = 2.964787873818262e-32

T: 23

i: 1
α(23,1) = emi(1,23) * Σ[[α((23-1),1) * trans(1,1)] + [α((23-1),2) * trans(2,1)] + [α((23-1),3) * trans(3,1)]]
α(23,1) = 0.05591 * [7.564069229375634e-30 * 0.97] + [1.1229646952196156e-31 * 0.0] + [2.964787873818262e-32 * 0.07] = 4.103359301989775e-31

i: 2
α(23,2) = emi(2,23) * Σ[[α((23-1),1) * trans(1,2)] + [α((23-1),2) * trans(2,2)] + [α((23-1),3) * trans(3,2)]]
α(23,2) = 0.07239 * [7.564069229375634e-30 * 0.0] + [1.1229646952196156e-31 * 0.6] + [2.964787873818262e-32 * 0.03] = 4.941871155472589e-33

i: 3
α(23,3) = emi(3,23) * Σ[[α((23-1),1) * trans(1,3)] + [α((23-1),2) * trans(2,3)] + [α((23-1),3) * trans(3,3)]]
α(23,3) = 0.06898 * [7.564069229375634e-30 * 0.03] + [1.1229646952196156e-31 * 0.4] + [2.964787873818262e-32 * 0.9] = 2.0592168658143752e-32

T: 24

i: 1
α(24,1) = emi(1,24) * Σ[[α((24-1),1) * trans(1,1)] + [α((24-1),2) * trans(2,1)] + [α((24-1),3) * trans(3,1)]]
α(24,1) = 0.04143 * [4.103359301989775e-31 * 0.97] + [4.941871155472589e-33 * 0.0] + [2.0592168658143752e-32 * 0.07] = 1.6549930408824813e-32

i: 2
α(24,2) = emi(2,24) * Σ[[α((24-1),1) * trans(1,2)] + [α((24-1),2) * trans(2,2)] + [α((24-1),3) * trans(3,2)]]
α(24,2) = 0.0399 * [4.103359301989775e-31 * 0.0] + [4.941871155472589e-33 * 0.6] + [2.0592168658143752e-32 * 0.03] = 1.4295722134581184e-34

i: 3
α(24,3) = emi(3,24) * Σ[[α((24-1),1) * trans(1,3)] + [α((24-1),2) * trans(2,3)] + [α((24-1),3) * trans(3,3)]]
α(24,3) = 0.00066 * [4.103359301989775e-31 * 0.03] + [4.941871155472589e-33 * 0.4] + [2.0592168658143752e-32 * 0.9] = 2.1661053585921907e-35

T: 25

i: 1
α(25,1) = emi(1,25) * Σ[[α((25-1),1) * trans(1,1)] + [α((25-1),2) * trans(2,1)] + [α((25-1),3) * trans(3,1)]]

α(25,1) = 0.06409 * [1.6549930408824813e-32 * 0.97] + [1.4295722134581184e-34 * 0.0] + [2.1661053585921907e-35 * 0.07] = 1.028961666689237e-33

i: 2
α(25,2) = emi(2,25) * Σ[[α((25-1),1) * trans(1,2)] + [α((25-1),2) * trans(2,2)] + [α((25-1),3) * trans(3,2)]]
α(25,2) = 0.06927 * [1.6549930408824813e-32 * 0.0] + [1.4295722134581184e-34 * 0.6] + [2.1661053585921907e-35 * 0.03] = 5.986601869031536e-36

i: 3
α(25,3) = emi(3,25) * Σ[[α((25-1),1) * trans(1,3)] + [α((25-1),2) * trans(2,3)] + [α((25-1),3) * trans(3,3)]]
α(25,3) = 0.04995 * [1.6549930408824813e-32 * 0.03] + [1.4295722134581184e-34 * 0.4] + [2.1661053585921907e-35 * 0.9] = 2.863012866406842e-35

T: 26

i: 1
α(26,1) = emi(1,26) * Σ[[α((26-1),1) * trans(1,1)] + [α((26-1),2) * trans(2,1)] + [α((26-1),3) * trans(3,1)]]
α(26,1) = 0.05591 * [1.028961666689237e-33 * 0.97] + [5.986601869031536e-36 * 0.0] + [2.863012866406842e-35 * 0.07] = 5.591541911560995e-35

i: 2
α(26,2) = emi(2,26) * Σ[[α((26-1),1) * trans(1,2)] + [α((26-1),2) * trans(2,2)] + [α((26-1),3) * trans(3,2)]]
α(26,2) = 0.07239 * [1.028961666689237e-33 * 0.0] + [5.986601869031536e-36 * 0.6] + [2.863012866406842e-35 * 0.03] = 3.221981159992731e-37

i: 3
α(26,3) = emi(3,26) * Σ[[α((26-1),1) * trans(1,3)] + [α((26-1),2) * trans(2,3)] + [α((26-1),3) * trans(3,3)]]
α(26,3) = 0.06898 * [1.028961666689237e-33 * 0.03] + [5.986601869031536e-36 * 0.4] + [2.863012866406842e-35 * 0.9] = 4.0719312395397217e-36

T: 27

i: 1
α(27,1) = emi(1,27) * Σ[[α((27-1),1) * trans(1,1)] + [α((27-1),2) * trans(2,1)] + [α((27-1),3) * trans(3,1)]]
α(27,1) = 0.05171 * [5.591541911560995e-35 * 0.97] + [3.221981159992731e-37 * 0.0] + [4.0719312395397217e-36 * 0.07] = 2.8193839023019066e-36

i: 2
α(27,2) = emi(2,27) * Σ[[α((27-1),1) * trans(1,2)] + [α((27-1),2) * trans(2,2)] + [α((27-1),3) * trans(3,2)]]
α(27,2) = 0.1033 * [5.591541911560995e-35 * 0.0] + [3.221981159992731e-37 * 0.6] + [4.0719312395397217e-36 * 0.03] = 3.258875414096854e-38

i: 3
α(27,3) = emi(3,27) * Σ[[α((27-1),1) * trans(1,3)] + [α((27-1),2) * trans(2,3)] + [α((27-1),3) * trans(3,3)]]
α(27,3) = 0.19429 * [5.591541911560995e-35 * 0.03] + [3.221981159992731e-37 * 0.4] + [4.0719312395397217e-36 * 0.9] = 1.0629761206593105e-36

T: 28

i: 1
α(28,1) = emi(1,28) * Σ[[α((28-1),1) * trans(1,1)] + [α((28-1),2) * trans(2,1)] + [α((28-1),3) * trans(3,1)]]
α(28,1) = 0.03296 * [2.8193839023019066e-36 * 0.97] + [3.258875414096854e-38 * 0.0] + [1.0629761206593105e-36 * 0.07] = 9.259158512285989e-38

i: 2
α(28,2) = emi(2,28) * Σ[[α((28-1),1) * trans(1,2)] + [α((28-1),2) * trans(2,2)] + [α((28-1),3) * trans(3,2)]]
α(28,2) = 0.0261 * [2.8193839023019066e-36 * 0.0] + [3.258875414096854e-38 * 0.6] + [1.0629761206593105e-36 * 0.03] = 1.3426501923238075e-39

i: 3
α(28,3) = emi(3,28) * Σ[[α((28-1),1) * trans(1,3)] + [α((28-1),2) * trans(2,3)] + [α((28-1),3) * trans(3,3)]]
α(28,3) = 0.06541 * [2.8193839023019066e-36 * 0.03] + [3.258875414096854e-38 * 0.4] + [1.0629761206593105e-36 * 0.9] = 6.896147044192427e-38

T: 29

i: 1
α(29,1) = emi(1,29) * Σ[[α((29-1),1) * trans(1,1)] + [α((29-1),2) * trans(2,1)] + [α((29-1),3) * trans(3,1)]]
α(29,1) = 0.11518 * [9.259158512285989e-38 * 0.97] + [1.3426501923238075e-39 * 0.0] + [6.896147044192427e-38 * 0.07] = 1.090076656280253e-38

i: 2
α(29,2) = emi(2,29) * Σ[[α((29-1),1) * trans(1,2)] + [α((29-1),2) * trans(2,2)] + [α((29-1),3) * trans(3,2)]]
α(29,2) = 0.07232 * [9.259158512285989e-38 * 0.0] + [1.3426501923238075e-39 * 0.6] + [6.896147044192427e-38 * 0.03] = 2.0787908341611353e-40

i: 3
α(29,3) = emi(3,29) * Σ[[α((29-1),1) * trans(1,3)] + [α((29-1),2) * trans(2,3)] + [α((29-1),3) * trans(3,3)]]
α(29,3) = 0.00852 * [9.259158512285989e-38 * 0.03] + [1.3426501923238075e-39 * 0.4] + [6.896147044192427e-38 * 0.9] = 5.5703871636151785e-40

T: 30

i: 1
α(30,1) = emi(1,30) * Σ[[α((30-1),1) * trans(1,1)] + [α((30-1),2) * trans(2,1)] + [α((30-1),3) * trans(3,1)]]
α(30,1) = 0.05877 * [1.090076656280253e-38 * 0.97] + [2.0787908341611353e-40 * 0.0] + [5.5703871636151785e-40 * 0.07] = 6.237105109442673e-40

i: 2
α(30,2) = emi(2,30) * Σ[[α((30-1),1) * trans(1,2)] + [α((30-1),2) * trans(2,2)] + [α((30-1),3) * trans(3,2)]]
α(30,2) = 0.04271 * [1.090076656280253e-38 * 0.0] + [2.0787908341611353e-40 * 0.6] + [5.5703871636151785e-40 * 0.03] = 6.040843098895337e-42

i: 3
α(30,3) = emi(3,30) * Σ[[α((30-1),1) * trans(1,3)] + [α((30-1),2) * trans(2,3)] + [α((30-1),3) * trans(3,3)]]
α(30,3) = 0.01879 * [1.090076656280253e-38 * 0.03] + [2.0787908341611353e-40 * 0.4] + [5.5703871636151785e-40 * 0.9] = 1.7127263034796927e-41

T: 31

i: 1

α(31,1) = emi(1,31) * Σ[[α((31-1),1) * trans(1,1)] + [α((31-1),2) * trans(2,1)] + [α((31-1),3) * trans(3,1)]]
α(31,1) = 0.04143 * [6.237105109442673e-40 * 0.97] + [6.040843098895337e-42 * 0.0] + [1.7127263034796927e-41 * 0.07] = 2.511478744989558e-41

i: 2
α(31,2) = emi(2,31) * Σ[[α((31-1),1) * trans(1,2)] + [α((31-1),2) * trans(2,2)] + [α((31-1),3) * trans(3,2)]]
α(31,2) = 0.0399 * [6.237105109442673e-40 * 0.0] + [6.040843098895337e-42 * 0.6] + [1.7127263034796927e-41 * 0.03] = 1.6511911764020629e-43

i: 3
α(31,3) = emi(3,31) * Σ[[α((31-1),1) * trans(1,3)] + [α((31-1),2) * trans(2,3)] + [α((31-1),3) * trans(3,3)]]
α(31,3) = 0.00066 * [6.237105109442673e-40 * 0.03] + [6.040843098895337e-42 * 0.4] + [1.7127263034796927e-41 * 0.9] = 2.4117844937474237e-44

T: 32

i: 1
α(32,1) = emi(1,32) * Σ[[α((32-1),1) * trans(1,1)] + [α((32-1),2) * trans(2,1)] + [α((32-1),3) * trans(3,1)]]
α(32,1) = 0.03004 * [2.511478744989558e-41 * 0.97] + [1.6511911764020629e-43 * 0.0] + [2.4117844937474237e-44 * 0.07] = 7.318654835493517e-43

i: 2
α(32,2) = emi(2,32) * Σ[[α((32-1),1) * trans(1,2)] + [α((32-1),2) * trans(2,2)] + [α((32-1),3) * trans(3,2)]]
α(32,2) = 0.05577 * [2.511478744989558e-41 * 0.0] + [1.6511911764020629e-43 * 0.6] + [2.4117844937474237e-44 * 0.03] = 5.5655674808414705e-45

i: 3
α(32,3) = emi(3,32) * Σ[[α((32-1),1) * trans(1,3)] + [α((32-1),2) * trans(2,3)] + [α((32-1),3) * trans(3,3)]]
α(32,3) = 0.09148 * [2.511478744989558e-41 * 0.03] + [1.6511911764020629e-43 * 0.4] + [2.4117844937474237e-44 * 0.9] = 7.6952731839576e-44

T: 33

i: 1
α(33,1) = emi(1,33) * Σ[[α((33-1),1) * trans(1,1)] + [α((33-1),2) * trans(2,1)] + [α((33-1),3) * trans(3,1)]]
α(33,1) = 0.05877 * [7.318654835493517e-43 * 0.97] + [5.5655674808414705e-45 * 0.0] + [7.6952731839576e-44 * 0.07] = 4.203795827766437e-44

i: 2
α(33,2) = emi(2,33) * Σ[[α((33-1),1) * trans(1,2)] + [α((33-1),2) * trans(2,2)] + [α((33-1),3) * trans(3,2)]]
α(33,2) = 0.04271 * [7.318654835493517e-43 * 0.0] + [5.5655674808414705e-45 * 0.6] + [7.6952731839576e-44 * 0.03] = 2.4122276757009223e-46

i: 3
α(33,3) = emi(3,33) * Σ[[α((33-1),1) * trans(1,3)] + [α((33-1),2) * trans(2,3)] + [α((33-1),3) * trans(3,3)]]
α(33,3) = 0.01879 * [7.318654835493517e-43 * 0.03] + [5.5655674808414705e-45 * 0.4] + [7.6952731839576e-44 * 0.9] = 1.7557310264018438e-45

T: 34

i: 1
α(34,1) = emi(1,34) * Σ[[α((34-1),1) * trans(1,1)] + [α((34-1),2) * trans(2,1)] + [α((34-1),3) * trans(3,1)]]
α(34,1) = 0.06409 * [4.203795827766437e-44 * 0.97] + [2.4122276757009223e-46 * 0.0] + [1.7557310264018438e-45 * 0.07] = 2.62126309973879e-45

i: 2
α(34,2) = emi(2,34) * Σ[[α((34-1),1) * trans(1,2)] + [α((34-1),2) * trans(2,2)] + [α((34-1),3) * trans(3,2)]]
α(34,2) = 0.06927 * [4.203795827766437e-44 * 0.0] + [2.4122276757009223e-46 * 0.6] + [1.7557310264018438e-45 * 0.03] = 1.3674285311713845e-47

i: 3
α(34,3) = emi(3,34) * Σ[[α((34-1),1) * trans(1,3)] + [α((34-1),2) * trans(2,3)] + [α((34-1),3) * trans(3,3)]]
α(34,3) = 0.04995 * [4.203795827766437e-44 * 0.03] + [2.4122276757009223e-46 * 0.4] + [1.7557310264018438e-45 * 0.9] = 1.4674239966702538e-46

T: 35

i: 1
α(35,1) = emi(1,35) * Σ[[α((35-1),1) * trans(1,1)] + [α((35-1),2) * trans(2,1)] + [α((35-1),3) * trans(3,1)]]
α(35,1) = 0.11518 * [2.62126309973879e-45 * 0.97] + [1.3674285311713845e-47 * 0.0] + [1.4674239966702538e-46 * 0.07] = 2.940426965846318e-46

i: 2
α(35,2) = emi(2,35) * Σ[[α((35-1),1) * trans(1,2)] + [α((35-1),2) * trans(2,2)] + [α((35-1),3) * trans(3,2)]]
α(35,2) = 0.07232 * [2.62126309973879e-45 * 0.0] + [1.3674285311713845e-47 * 0.6] + [1.4674239966702538e-46 * 0.03] = 9.117268985634654e-49

i: 3
α(35,3) = emi(3,35) * Σ[[α((35-1),1) * trans(1,3)] + [α((35-1),2) * trans(2,3)] + [α((35-1),3) * trans(3,3)]]
α(35,3) = 0.00852 * [2.62126309973879e-45 * 0.03] + [1.3674285311713845e-47 * 0.4] + [1.4674239966702538e-46 * 0.9] = 1.841817533282306e-48

T: 36

i: 1
α(36,1) = emi(1,36) * Σ[[α((36-1),1) * trans(1,1)] + [α((36-1),2) * trans(2,1)] + [α((36-1),3) * trans(3,1)]]
α(36,1) = 0.04143 * [2.940426965846318e-46 * 0.97] + [9.117268985634654e-49 * 0.0] + [1.841817533282306e-48 * 0.07] = 1.1822064706944527e-47

i: 2
α(36,2) = emi(2,36) * Σ[[α((36-1),1) * trans(1,2)] + [α((36-1),2) * trans(2,2)] + [α((36-1),3) * trans(3,2)]]
α(36,2) = 0.0399 * [2.940426965846318e-46 * 0.0] + [9.117268985634654e-49 * 0.6] + [1.841817533282306e-48 * 0.03] = 2.4031397538948284e-50

i: 3
α(36,3) = emi(3,36) * Σ[[α((36-1),1) * trans(1,3)] + [α((36-1),2) * trans(2,3)] + [α((36-1),3) * trans(3,3)]]
α(36,3) = 0.00066 * [2.940426965846318e-46 * 0.03] + [9.117268985634654e-49 * 0.4] + [1.841817533282306e-48 * 0.9] = 7.156780908366153e-51

T: 37

i: 1
α(37,1) = emi(1,37) * Σ[[α((37-1),1) * trans(1,1)] + [α((37-1),2) * trans(2,1)] + [α((37-1),3) * trans(3,1)]]
α(37,1) = 0.06901 * [1.1822064706944527e-47 * 0.97] + [2.4031397538948284e-50 * 0.0] + [7.156780908366153e-51 * 0.07] = 7.914000371249885e-49

i: 2
α(37,2) = emi(2,37) * Σ[[α((37-1),1) * trans(1,2)] + [α((37-1),2) * trans(2,2)] + [α((37-1),3) * trans(3,2)]]
α(37,2) = 0.07052 * [1.1822064706944527e-47 * 0.0] + [2.4031397538948284e-50 * 0.6] + [7.156780908366153e-51 * 0.03] = 1.031957378357719e-51

i: 3
α(37,3) = emi(3,37) * Σ[[α((37-1),1) * trans(1,3)] + [α((37-1),2) * trans(2,3)] + [α((37-1),3) * trans(3,3)]]
α(37,3) = 0.09536 * [1.1822064706944527e-47 * 0.03] + [2.4031397538948284e-50 * 0.4] + [7.156780908366153e-51 * 0.9] = 3.5351439906032163e-50

T: 38

i: 1
α(38,1) = emi(1,38) * Σ[[α((38-1),1) * trans(1,1)] + [α((38-1),2) * trans(2,1)] + [α((38-1),3) * trans(3,1)]]
α(38,1) = 0.05877 * [7.914000371249885e-49 * 0.97] + [1.031957378357719e-51 * 0.0] + [3.5351439906032163e-50 * 0.07] = 4.526069506500993e-50

i: 2
α(38,2) = emi(2,38) * Σ[[α((38-1),1) * trans(1,2)] + [α((38-1),2) * trans(2,2)] + [α((38-1),3) * trans(3,2)]]
α(38,2) = 0.04271 * [7.914000371249885e-49 * 0.0] + [1.031957378357719e-51 * 0.6] + [3.5351439906032163e-50 * 0.03] = 7.174073972939392e-53

i: 3
α(38,3) = emi(3,38) * Σ[[α((38-1),1) * trans(1,3)] + [α((38-1),2) * trans(2,3)] + [α((38-1),3) * trans(3,3)]]
α(38,3) = 0.01879 * [7.914000371249885e-49 * 0.03] + [1.031957378357719e-51 * 0.4] + [3.5351439906032163e-50 * 0.9] = 1.0516965928340026e-51

T: 39

i: 1
α(39,1) = emi(1,39) * Σ[[α((39-1),1) * trans(1,1)] + [α((39-1),2) * trans(2,1)] + [α((39-1),3) * trans(3,1)]]
α(39,1) = 0.06409 * [4.526069506500993e-50 * 0.97] + [7.174073972939392e-53 * 0.0] + [1.0516965928340026e-51 * 0.07] = 2.8184534347394225e-51

i: 2
α(39,2) = emi(2,39) * Σ[[α((39-1),1) * trans(1,2)] + [α((39-1),2) * trans(2,2)] + [α((39-1),3) * trans(3,2)]]
α(39,2) = 0.06927 * [4.526069506500993e-50 * 0.0] + [7.174073972939392e-53 * 0.6] + [1.0516965928340026e-51 * 0.03] = 5.1672193142014106e-54

i: 3
α(39,3) = emi(3,39) * Σ[[α((39-1),1) * trans(1,3)] + [α((39-1),2) * trans(2,3)] + [α((39-1),3) * trans(3,3)]]
α(39,3) = 0.04995 * [4.526069506500993e-50 * 0.03] + [7.174073972939392e-53 * 0.4] + [1.0516965928340026e-51 * 0.9] = 1.1653555186556327e-52

T: 40

i: 1
α(40,1) = emi(1,40) * Σ[[α((40-1),1) * trans(1,1)] + [α((40-1),2) * trans(2,1)] + [α((40-1),3) * trans(3,1)]]
α(40,1) = 0.06409 * [2.8184534347394225e-51 * 0.97] + [5.1672193142014106e-54 * 0.0] + [1.1653555186556327e-52 * 0.07] = 1.7573845365981057e-52

i: 2
α(40,2) = emi(2,40) * Σ[[α((40-1),1) * trans(1,2)] + [α((40-1),2) * trans(2,2)] + [α((40-1),3) * trans(3,2)]]
α(40,2) = 0.06927 * [2.8184534347394225e-51 * 0.0] + [5.1672193142014106e-54 * 0.6] + [1.1653555186556327e-52 * 0.03] = 4.56932499468666e-55

i: 3
α(40,3) = emi(3,40) * Σ[[α((40-1),1) * trans(1,3)] + [α((40-1),2) * trans(2,3)] + [α((40-1),3) * trans(3,3)]]
α(40,3) = 0.04995 * [2.8184534347394225e-51 * 0.03] + [5.1672193142014106e-54 * 0.4] + [1.1653555186556327e-52 * 0.9] = 9.565549247971168e-54

T: 41

i: 1
α(41,1) = emi(1,41) * Σ[[α((41-1),1) * trans(1,1)] + [α((41-1),2) * trans(2,1)] + [α((41-1),3) * trans(3,1)]]
α(41,1) = 0.04143 * [1.7573845365981057e-52 * 0.97] + [4.56932499468666e-55 * 0.0] + [9.565549247971168e-54 * 0.07] = 7.090159860446213e-54

i: 2
α(41,2) = emi(2,41) * Σ[[α((41-1),1) * trans(1,2)] + [α((41-1),2) * trans(2,2)] + [α((41-1),3) * trans(3,2)]]
α(41,2) = 0.0399 * [1.7573845365981057e-52 * 0.0] + [4.56932499468666e-55 * 0.6] + [9.565549247971168e-54 * 0.03] = 2.2388926487101352e-56

i: 3
α(41,3) = emi(3,41) * Σ[[α((41-1),1) * trans(1,3)] + [α((41-1),2) * trans(2,3)] + [α((41-1),3) * trans(3,3)]]
α(41,3) = 0.00066 * [1.7573845365981057e-52 * 0.03] + [4.56932499468666e-55 * 0.4] + [9.565549247971168e-54 * 0.9] = 9.28218781561885e-57

T: 42

i: 1
α(42,1) = emi(1,42) * Σ[[α((42-1),1) * trans(1,1)] + [α((42-1),2) * trans(2,1)] + [α((42-1),3) * trans(3,1)]]
α(42,1) = 0.06901 * [7.090159860446213e-54 * 0.97] + [2.2388926487101352e-56 * 0.0] + [9.28218781561885e-57 * 0.07] = 4.7465801347499226e-55

i: 2
α(42,2) = emi(2,42) * Σ[[α((42-1),1) * trans(1,2)] + [α((42-1),2) * trans(2,2)] + [α((42-1),3) * trans(3,2)]]
α(42,2) = 0.07052 * [7.090159860446213e-54 * 0.0] + [2.2388926487101352e-56 * 0.6] + [9.28218781561885e-57 * 0.03] = 9.669576540649557e-58

i: 3
α(42,3) = emi(3,42) * Σ[[α((42-1),1) * trans(1,3)] + [α((42-1),2) * trans(2,3)] + [α((42-1),3) * trans(3,3)]]
α(42,3) = 0.09536 * [7.090159860446213e-54 * 0.03] + [2.2388926487101352e-56 * 0.4] + [9.28218781561885e-57 * 0.9] = 2.1934167027776192e-56

T: 43

i: 1
α(43,1) = emi(1,43) * Σ[[α((43-1),1) * trans(1,1)] + [α((43-1),2) * trans(2,1)] + [α((43-1),3) * trans(3,1)]]
α(43,1) = 0.06409 * [4.7465801347499226e-55 * 0.97] + [9.669576540649557e-58 * 0.0] + [2.1934167027776192e-56 * 0.07] = 2.9606610374640597e-56

i: 2
α(43,2) = emi(2,43) * Σ[[α((43-1),1) * trans(1,2)] + [α((43-1),2) * trans(2,2)] + [α((43-1),3) * trans(3,2)]]
α(43,2) = 0.06927 * [4.7465801347499226e-55 * 0.0] + [9.669576540649557e-58 * 0.6] + [2.1934167027776192e-56 * 0.03] = 8.57700865186694e-59

i: 3
α(43,3) = emi(3,43) * Σ[[α((43-1),1) * trans(1,3)] + [α((43-1),2) * trans(2,3)] + [α((43-1),3) * trans(3,3)]]
α(43,3) = 0.04995 * [4.7465801347499226e-55 * 0.03] + [9.669576540649557e-58 * 0.4] + [2.1934167027776192e-56 * 0.9] = 1.7166453258541723e-57

T: 44

i: 1
α(44,1) = emi(1,44) * Σ[[α((44-1),1) * trans(1,1)] + [α((44-1),2) * trans(2,1)] + [α((44-1),3) * trans(3,1)]]
α(44,1) = 0.05877 * [2.9606610374640597e-56 * 0.97] + [8.57700865186694e-59 * 0.0] + [1.7166453258541723e-57 * 0.07] = 1.6948431841721305e-57

i: 2
α(44,2) = emi(2,44) * Σ[[α((44-1),1) * trans(1,2)] + [α((44-1),2) * trans(2,2)] + [α((44-1),3) * trans(3,2)]]
α(44,2) = 0.04271 * [2.9606610374640597e-56 * 0.0] + [8.57700865186694e-59 * 0.6] + [1.7166453258541723e-57 * 0.03] = 4.3974818931443726e-60

i: 3
α(44,3) = emi(3,44) * Σ[[α((44-1),1) * trans(1,3)] + [α((44-1),2) * trans(2,3)] + [α((44-1),3) * trans(3,3)]]
α(44,3) = 0.01879 * [2.9606610374640597e-56 * 0.03] + [8.57700865186694e-59 * 0.4] + [1.7166453258541723e-57 * 0.9] = 4.636408334397914e-59

T: 45

i: 1
α(45,1) = emi(1,45) * Σ[[α((45-1),1) * trans(1,1)] + [α((45-1),2) * trans(2,1)] + [α((45-1),3) * trans(3,1)]]
α(45,1) = 0.01482 * [1.6948431841721305e-57 * 0.97] + [4.3974818931443726e-60 * 0.0] + [4.636408334397914e-59 * 0.07] = 2.441214680980909e-59

i: 2
α(45,2) = emi(2,45) * Σ[[α((45-1),1) * trans(1,2)] + [α((45-1),2) * trans(2,2)] + [α((45-1),3) * trans(3,2)]]
α(45,2) = 0.01439 * [1.6948431841721305e-57 * 0.0] + [4.3974818931443726e-60 * 0.6] + [4.636408334397914e-59 * 0.03] = 5.79832334450043e-62

i: 3
α(45,3) = emi(3,45) * Σ[[α((45-1),1) * trans(1,3)] + [α((45-1),2) * trans(2,3)] + [α((45-1),3) * trans(3,3)]]
α(45,3) = 0.04444 * [1.6948431841721305e-57 * 0.03] + [4.3974818931443726e-60 * 0.4] + [4.636408334397914e-59 * 0.9] = 4.192112448696609e-60

T: 46

i: 1
α(46,1) = emi(1,46) * Σ[[α((46-1),1) * trans(1,1)] + [α((46-1),2) * trans(2,1)] + [α((46-1),3) * trans(3,1)]]
α(46,1) = 0.06521 * [2.441214680980909e-59 * 0.97] + [5.79832334450043e-62 * 0.0] + [4.192112448696609e-60 * 0.07] = 1.5632943463581866e-60

i: 2
α(46,2) = emi(2,46) * Σ[[α((46-1),1) * trans(1,2)] + [α((46-1),2) * trans(2,2)] + [α((46-1),3) * trans(3,2)]]
α(46,2) = 0.05586 * [2.441214680980909e-59 * 0.0] + [5.79832334450043e-62 * 0.6] + [4.192112448696609e-60 * 0.03] = 8.968508093668541e-63

i: 3
α(46,3) = emi(3,46) * Σ[[α((46-1),1) * trans(1,3)] + [α((46-1),2) * trans(2,3)] + [α((46-1),3) * trans(3,3)]]
α(46,3) = 0.02246 * [2.441214680980909e-59 * 0.03] + [5.79832334450043e-62 * 0.4] + [4.192112448696609e-60 * 0.9] = 1.0170918692767255e-61

T: 47

i: 1
α(47,1) = emi(1,47) * Σ[[α((47-1),1) * trans(1,1)] + [α((47-1),2) * trans(2,1)] + [α((47-1),3) * trans(3,1)]]
α(47,1) = 0.04294 * [1.5632943463581866e-60 * 0.97] + [8.968508093668541e-63 * 0.0] + [1.0170918692767255e-61 * 0.07] = 6.541974092970911e-62

i: 2
α(47,2) = emi(2,47) * Σ[[α((47-1),1) * trans(1,2)] + [α((47-1),2) * trans(2,2)] + [α((47-1),3) * trans(3,2)]]
α(47,2) = 0.04257 * [1.5632943463581866e-60 * 0.0] + [8.968508093668541e-63 * 0.6] + [1.0170918692767255e-61 * 0.03] = 3.589664363538124e-64

i: 3
α(47,3) = emi(3,47) * Σ[[α((47-1),1) * trans(1,3)] + [α((47-1),2) * trans(2,3)] + [α((47-1),3) * trans(3,3)]]
α(47,3) = 0.11935 * [1.5632943463581866e-60 * 0.03] + [8.968508093668541e-63 * 0.4] + [1.0170918692767255e-61 * 0.9] = 1.695062429736317e-62

T: 48

i: 1
α(48,1) = emi(1,48) * Σ[[α((48-1),1) * trans(1,1)] + [α((48-1),2) * trans(2,1)] + [α((48-1),3) * trans(3,1)]]
α(48,1) = 0.04143 * [6.541974092970911e-62 * 0.97] + [3.589664363538124e-64 * 0.0] + [1.695062429736317e-62 * 0.07] = 2.6781881762410957e-63

i: 2
α(48,2) = emi(2,48) * Σ[[α((48-1),1) * trans(1,2)] + [α((48-1),2) * trans(2,2)] + [α((48-1),3) * trans(3,2)]]
α(48,2) = 0.0399 * [6.541974092970911e-62 * 0.0] + [3.589664363538124e-64 * 0.6] + [1.695062429736317e-62 * 0.03] = 2.888355377025398e-65

i: 3
α(48,3) = emi(3,48) * Σ[[α((48-1),1) * trans(1,3)] + [α((48-1),2) * trans(2,3)] + [α((48-1),3) * trans(3,3)]]
α(48,3) = 0.00066 * [6.541974092970911e-62 * 0.03] + [3.589664363538124e-64 * 0.4] + [1.695062429736317e-62 * 0.9] = 1.145874884223937e-65

T: 49

i: 1
α(49,1) = emi(1,49) * Σ[[α((49-1),1) * trans(1,1)] + [α((49-1),2) * trans(2,1)] + [α((49-1),3) * trans(3,1)]]
α(49,1) = 0.05591 * [2.6781881762410957e-63 * 0.97] + [2.888355377025398e-65 * 0.0] + [1.145874884223937e-65 * 0.07] = 1.4529022201097434e-64

i: 2
α(49,2) = emi(2,49) * Σ[[α((49-1),1) * trans(1,2)] + [α((49-1),2) * trans(2,2)] + [α((49-1),3) * trans(3,2)]]
α(49,2) = 0.07239 * [2.6781881762410957e-63 * 0.0] + [2.888355377025398e-65 * 0.6] + [1.145874884223937e-65 * 0.03] = 1.2794132393179025e-66

i: 3
α(49,3) = emi(3,49) * Σ[[α((49-1),1) * trans(1,3)] + [α((49-1),2) * trans(2,3)] + [α((49-1),3) * trans(3,3)]]
α(49,3) = 0.06898 * [2.6781881762410957e-63 * 0.03] + [2.888355377025398e-65 * 0.4] + [1.145874884223937e-65 * 0.9] = 7.050579673166075e-66

T: 50

i: 1
α(50,1) = emi(1,50) * Σ[[α((50-1),1) * trans(1,1)] + [α((50-1),2) * trans(2,1)] + [α((50-1),3) * trans(3,1)]]
α(50,1) = 0.04621 * [1.4529022201097434e-64 * 0.97] + [1.2794132393179025e-66 * 0.0] + [7.050579673166075e-66 * 0.07] = 6.535251834422101e-66

i: 2
α(50,2) = emi(2,50) * Σ[[α((50-1),1) * trans(1,2)] + [α((50-1),2) * trans(2,2)] + [α((50-1),3) * trans(3,2)]]
α(50,2) = 0.05409 * [1.4529022201097434e-64 * 0.0] + [1.2794132393179025e-66 * 0.6] + [7.050579673166075e-66 * 0.03] = 5.29630529044698e-68

i: 3
α(50,3) = emi(3,50) * Σ[[α((50-1),1) * trans(1,3)] + [α((50-1),2) * trans(2,3)] + [α((50-1),3) * trans(3,3)]]
α(50,3) = 0.00457 * [1.4529022201097434e-64 * 0.03] + [1.2794132393179025e-66 * 0.4] + [7.050579673166075e-66 * 0.9] = 5.125709103490978e-68

T: 51

i: 1
α(51,1) = emi(1,51) * Σ[[α((51-1),1) * trans(1,1)] + [α((51-1),2) * trans(2,1)] + [α((51-1),3) * trans(3,1)]]
α(51,1) = 0.05877 * [6.535251834422101e-66 * 0.97] + [5.29630529044698e-68 * 0.0] + [5.125709103490978e-68 * 0.07] = 3.727653143465257e-67

i: 2
α(51,2) = emi(2,51) * Σ[[α((51-1),1) * trans(1,2)] + [α((51-1),2) * trans(2,2)] + [α((51-1),3) * trans(3,2)]]
α(51,2) = 0.04271 * [6.535251834422101e-66 * 0.0] + [5.29630529044698e-68 * 0.6] + [5.125709103490978e-68 * 0.03] = 1.4229069044729729e-69

i: 3
α(51,3) = emi(3,51) * Σ[[α((51-1),1) * trans(1,3)] + [α((51-1),2) * trans(2,3)] + [α((51-1),3) * trans(3,3)]]
α(51,3) = 0.01879 * [6.535251834422101e-66 * 0.03] + [5.29630529044698e-68 * 0.4] + [5.125709103490978e-68 * 0.9] = 4.948800431185094e-69

T: 52

i: 1
α(52,1) = emi(1,52) * Σ[[α((52-1),1) * trans(1,1)] + [α((52-1),2) * trans(2,1)] + [α((52-1),3) * trans(3,1)]]
α(52,1) = 0.11518 * [3.727653143465257e-67 * 0.97] + [1.4229069044729729e-69 * 0.0] + [4.948800431185094e-69 * 0.07] = 4.168695583759632e-68

i: 2
α(52,2) = emi(2,52) * Σ[[α((52-1),1) * trans(1,2)] + [α((52-1),2) * trans(2,2)] + [α((52-1),3) * trans(3,2)]]
α(52,2) = 0.07232 * [3.727653143465257e-67 * 0.0] + [1.4229069044729729e-69 * 0.6] + [4.948800431185094e-69 * 0.03] = 7.247969381439041e-71

i: 3
α(52,3) = emi(3,52) * Σ[[α((52-1),1) * trans(1,3)] + [α((52-1),2) * trans(2,3)] + [α((52-1),3) * trans(3,3)]]
α(52,3) = 0.00852 * [3.727653143465257e-67 * 0.03] + [1.4229069044729729e-69 * 0.4] + [4.948800431185094e-69 * 0.9] = 1.3807548278374315e-70

T: 53

i: 1
α(53,1) = emi(1,53) * Σ[[α((53-1),1) * trans(1,1)] + [α((53-1),2) * trans(2,1)] + [α((53-1),3) * trans(3,1)]]
α(53,1) = 0.06521 * [4.168695583759632e-68 * 0.97] + [7.247969381439041e-71 * 0.0] + [1.3807548278374315e-70 * 0.07] = 2.6374844716208294e-69

i: 2
α(53,2) = emi(2,53) * Σ[[α((53-1),1) * trans(1,2)] + [α((53-1),2) * trans(2,2)] + [α((53-1),3) * trans(3,2)]]
α(53,2) = 0.05586 * [4.168695583759632e-68 * 0.0] + [7.247969381439041e-71 * 0.6] + [1.3807548278374315e-70 * 0.03] = 2.6606163119321057e-72

i: 3
α(53,3) = emi(3,53) * Σ[[α((53-1),1) * trans(1,3)] + [α((53-1),2) * trans(2,3)] + [α((53-1),3) * trans(3,3)]]
α(53,3) = 0.02246 * [4.168695583759632e-68 * 0.03] + [7.247969381439041e-71 * 0.4] + [1.3807548278374315e-70 * 0.9] = 3.153088622159147e-71

T: 54

i: 1
α(54,1) = emi(1,54) * Σ[[α((54-1),1) * trans(1,1)] + [α((54-1),2) * trans(2,1)] + [α((54-1),3) * trans(3,1)]]
α(54,1) = 0.05877 * [2.6374844716208294e-69 * 0.97] + [2.6606163119321057e-72 * 0.0] + [3.153088622159147e-71 * 0.07] = 1.504845284380685e-70

i: 2
α(54,2) = emi(2,54) * Σ[[α((54-1),1) * trans(1,2)] + [α((54-1),2) * trans(2,2)] + [α((54-1),3) * trans(3,2)]]
α(54,2) = 0.04271 * [2.6374844716208294e-69 * 0.0] + [2.6606163119321057e-72 * 0.6] + [3.153088622159147e-71 * 0.03] = 1.0858147812529727e-73

i: 3
α(54,3) = emi(3,54) * Σ[[α((54-1),1) * trans(1,3)] + [α((54-1),2) * trans(2,3)] + [α((54-1),3) * trans(3,3)]]
α(54,3) = 0.01879 * [2.6374844716208294e-69 * 0.03] + [2.6606163119321057e-72 * 0.4] + [3.153088622159147e-71 * 0.9] = 2.0399660057464765e-72

T: 55

i: 1
α(55,1) = emi(1,55) * Σ[[α((55-1),1) * trans(1,1)] + [α((55-1),2) * trans(2,1)] + [α((55-1),3) * trans(3,1)]]
α(55,1) = 0.05171 * [1.504845284380685e-70 * 0.97] + [1.0858147812529727e-73 * 0.0] + [2.0399660057464765e-72 * 0.07] = 7.555492381517546e-72

i: 2
α(55,2) = emi(2,55) * Σ[[α((55-1),1) * trans(1,2)] + [α((55-1),2) * trans(2,2)] + [α((55-1),3) * trans(3,2)]]
α(55,2) = 0.1033 * [1.504845284380685e-70 * 0.0] + [1.0858147812529727e-73 * 0.6] + [2.0399660057464765e-72 * 0.03] = 1.3051734666014256e-74

i: 3
α(55,3) = emi(3,55) * Σ[[α((55-1),1) * trans(1,3)] + [α((55-1),2) * trans(2,3)] + [α((55-1),3) * trans(3,3)]]
α(55,3) = 0.19429 * [1.504845284380685e-70 * 0.03] + [1.0858147812529727e-73 * 0.4] + [2.0399660057464765e-72 * 0.9] = 1.24227818479179e-72

T: 56

i: 1
α(56,1) = emi(1,56) * Σ[[α((56-1),1) * trans(1,1)] + [α((56-1),2) * trans(2,1)] + [α((56-1),3) * trans(3,1)]]
α(56,1) = 0.06409 * [7.555492381517546e-72 * 0.97] + [1.3051734666014256e-74 * 0.0] + [1.24227818479179e-72 * 0.07] = 4.7527779414994706e-73

i: 2
α(56,2) = emi(2,56) * Σ[[α((56-1),1) * trans(1,2)] + [α((56-1),2) * trans(2,2)] + [α((56-1),3) * trans(3,2)]]
α(56,2) = 0.06927 * [7.555492381517546e-72 * 0.0] + [1.3051734666014256e-74 * 0.6] + [1.24227818479179e-72 * 0.03] = 3.1240344920047033e-75

i: 3
α(56,3) = emi(3,56) * Σ[[α((56-1),1) * trans(1,3)] + [α((56-1),2) * trans(2,3)] + [α((56-1),3) * trans(3,3)]]
α(56,3) = 0.04995 * [7.555492381517546e-72 * 0.03] + [1.3051734666014256e-74 * 0.4] + [1.24227818479179e-72 * 0.9] = 6.742929478964592e-74

T: 57

i: 1
α(57,1) = emi(1,57) * Σ[[α((57-1),1) * trans(1,1)] + [α((57-1),2) * trans(2,1)] + [α((57-1),3) * trans(3,1)]]
α(57,1) = 0.03004 * [4.7527779414994706e-73 * 0.97] + [3.1240344920047033e-75 * 0.0] + [6.742929478964592e-74 * 0.07] = 1.3990814909260143e-74

i: 2
α(57,2) = emi(2,57) * Σ[[α((57-1),1) * trans(1,2)] + [α((57-1),2) * trans(2,2)] + [α((57-1),3) * trans(3,2)]]
α(57,2) = 0.05577 * [4.7527779414994706e-73 * 0.0] + [3.1240344920047033e-75 * 0.6] + [6.742929478964592e-74 * 0.03] = 2.1735239528401797e-76

i: 3
α(57,3) = emi(3,57) * Σ[[α((57-1),1) * trans(1,3)] + [α((57-1),2) * trans(2,3)] + [α((57-1),3) * trans(3,3)]]
α(57,3) = 0.09148 * [4.7527779414994706e-73 * 0.03] + [3.1240344920047033e-75 * 0.4] + [6.742929478964592e-74 * 0.9] = 6.97025574701768e-75

T: 58

i: 1
α(58,1) = emi(1,58) * Σ[[α((58-1),1) * trans(1,1)] + [α((58-1),2) * trans(2,1)] + [α((58-1),3) * trans(3,1)]]
α(58,1) = 0.04621 * [1.3990814909260143e-74 * 0.97] + [2.1735239528401797e-76 * 0.0] + [6.97025574701768e-75 * 0.07] = 6.49666776513082e-76

i: 2
α(58,2) = emi(2,58) * Σ[[α((58-1),1) * trans(1,2)] + [α((58-1),2) * trans(2,2)] + [α((58-1),3) * trans(3,2)]]
α(58,2) = 0.05409 * [1.3990814909260143e-74 * 0.0] + [2.1735239528401797e-76 * 0.6] + [6.97025574701768e-75 * 0.03] = 1.8364588637233103e-77

i: 3
α(58,3) = emi(3,58) * Σ[[α((58-1),1) * trans(1,3)] + [α((58-1),2) * trans(2,3)] + [α((58-1),3) * trans(3,3)]]
α(58,3) = 0.00457 * [1.3990814909260143e-74 * 0.03] + [2.1735239528401797e-76 * 0.4] + [6.97025574701768e-75 * 0.9] = 3.098412279012247e-77

T: 59

i: 1
α(59,1) = emi(1,59) * Σ[[α((59-1),1) * trans(1,1)] + [α((59-1),2) * trans(2,1)] + [α((59-1),3) * trans(3,1)]]
α(59,1) = 0.05591 * [6.49666776513082e-76 * 0.97] + [1.8364588637233103e-77 * 0.0] + [3.098412279012247e-77 * 0.07] = 3.5354445951964724e-77

i: 2
α(59,2) = emi(2,59) * Σ[[α((59-1),1) * trans(1,2)] + [α((59-1),2) * trans(2,2)] + [α((59-1),3) * trans(3,2)]]
α(59,2) = 0.07239 * [6.49666776513082e-76 * 0.0] + [1.8364588637233103e-77 * 0.6] + [3.098412279012247e-77 * 0.03] = 8.649357623328916e-79

i: 3
α(59,3) = emi(3,59) * Σ[[α((59-1),1) * trans(1,3)] + [α((59-1),2) * trans(2,3)] + [α((59-1),3) * trans(3,3)]]
α(59,3) = 0.06898 * [6.49666776513082e-76 * 0.03] + [1.8364588637233103e-77 * 0.4] + [3.098412279012247e-77 * 0.9] = 3.7746924680510904e-78

T: 60

i: 1
α(60,1) = emi(1,60) * Σ[[α((60-1),1) * trans(1,1)] + [α((60-1),2) * trans(2,1)] + [α((60-1),3) * trans(3,1)]]
α(60,1) = 0.03296 * [3.5354445951964724e-77 * 0.97] + [8.649357623328916e-79 * 0.0] + [3.7746924680510904e-78 * 0.07] = 1.139033032881742e-78

i: 2
α(60,2) = emi(2,60) * Σ[[α((60-1),1) * trans(1,2)] + [α((60-1),2) * trans(2,2)] + [α((60-1),3) * trans(3,2)]]
α(60,2) = 0.0261 * [3.5354445951964724e-77 * 0.0] + [8.649357623328916e-79 * 0.6] + [3.7746924680510904e-78 * 0.03] = 1.6500478240617085e-80

i: 3
α(60,3) = emi(3,60) * Σ[[α((60-1),1) * trans(1,3)] + [α((60-1),2) * trans(2,3)] + [α((60-1),3) * trans(3,3)]]

α(60,3) = 0.06541 * [3.5354445951964724e-77 * 0.03] + [8.649357623328916e-79 * 0.4] + [3.7746924680510904e-78 * 0.9] = 3.142185794789177e-79

T: 61

i: 1
α(61,1) = emi(1,61) * Σ[[α((61-1),1) * trans(1,1)] + [α((61-1),2) * trans(2,1)] + [α((61-1),3) * trans(3,1)]]
α(61,1) = 0.05171 * [1.139033032881742e-78 * 0.97] + [1.6500478240617085e-80 * 0.0] + [3.142185794789177e-79 * 0.07] = 5.826979317854527e-80

i: 2
α(61,2) = emi(2,61) * Σ[[α((61-1),1) * trans(1,2)] + [α((61-1),2) * trans(2,2)] + [α((61-1),3) * trans(3,2)]]
α(61,2) = 0.1033 * [1.139033032881742e-78 * 0.0] + [1.6500478240617085e-80 * 0.6] + [3.142185794789177e-79 * 0.03] = 1.9964630191586128e-81

i: 3
α(61,3) = emi(3,61) * Σ[[α((61-1),1) * trans(1,3)] + [α((61-1),2) * trans(2,3)] + [α((61-1),3) * trans(3,3)]]
α(61,3) = 0.19429 * [1.139033032881742e-78 * 0.03] + [1.6500478240617085e-80 * 0.4] + [3.142185794789177e-79 * 0.9] = 6.2866008031968645e-80

T: 62

i: 1
α(62,1) = emi(1,62) * Σ[[α((62-1),1) * trans(1,1)] + [α((62-1),2) * trans(2,1)] + [α((62-1),3) * trans(3,1)]]
α(62,1) = 0.03953 * [5.826979317854527e-80 * 0.97] + [1.9964630191586128e-81 * 0.0] + [6.2866008031968645e-80 * 0.07] = 2.4082593074427185e-81

i: 2
α(62,2) = emi(2,62) * Σ[[α((62-1),1) * trans(1,2)] + [α((62-1),2) * trans(2,2)] + [α((62-1),3) * trans(3,2)]]
α(62,2) = 0.05868 * [5.826979317854527e-80 * 0.0] + [1.9964630191586128e-81 * 0.6] + [6.2866008031968645e-80 * 0.03] = 1.8096079051801405e-82

i: 3
α(62,3) = emi(3,62) * Σ[[α((62-1),1) * trans(1,3)] + [α((62-1),2) * trans(2,3)] + [α((62-1),3) * trans(3,3)]]
α(62,3) = 0.01557 * [5.826979317854527e-80 * 0.03] + [1.9964630191586128e-81 * 0.4] + [6.2866008031968645e-80 * 0.9] = 9.205931626289951e-82

T: 63

i: 1
α(63,1) = emi(1,63) * Σ[[α((63-1),1) * trans(1,1)] + [α((63-1),2) * trans(2,1)] + [α((63-1),3) * trans(3,1)]]
α(63,1) = 0.03953 * [2.4082593074427185e-81 * 0.97] + [1.8096079051801405e-82 * 0.0] + [9.205931626289951e-82 * 0.07] = 9.488990905082502e-83

i: 2
α(63,2) = emi(2,63) * Σ[[α((63-1),1) * trans(1,2)] + [α((63-1),2) * trans(2,2)] + [α((63-1),3) * trans(3,2)]]
α(63,2) = 0.05868 * [2.4082593074427185e-81 * 0.0] + [1.8096079051801405e-82 * 0.6] + [9.205931626289951e-82 * 0.03] = 7.991879716050322e-84

i: 3
α(63,3) = emi(3,63) * Σ[[α((63-1),1) * trans(1,3)] + [α((63-1),2) * trans(2,3)] + [α((63-1),3) * trans(3,3)]]
α(63,3) = 0.01557 * [2.4082593074427185e-81 * 0.03] + [1.8096079051801405e-82 * 0.4] + [9.205931626289951e-82 * 0.9] = 1.5152193713772794e-83

T: 64

i: 1
α(64,1) = emi(1,64) * Σ[[α((64-1),1) * trans(1,1)] + [α((64-1),2) * trans(2,1)] + [α((64-1),3) * trans(3,1)]]
α(64,1) = 0.06521 * [9.488990905082502e-83 * 0.97] + [7.991879716050322e-84 * 0.0] + [1.5152193713772794e-83 * 0.07] = 6.071303058773428e-84

i: 2
α(64,2) = emi(2,64) * Σ[[α((64-1),1) * trans(1,2)] + [α((64-1),2) * trans(2,2)] + [α((64-1),3) * trans(3,2)]]
α(64,2) = 0.05586 * [9.488990905082502e-83 * 0.0] + [7.991879716050322e-84 * 0.6] + [1.5152193713772794e-83 * 0.03] = 2.93247886788683e-85

i: 3
α(64,3) = emi(3,64) * Σ[[α((64-1),1) * trans(1,3)] + [α((64-1),2) * trans(2,3)] + [α((64-1),3) * trans(3,3)]]
α(64,3) = 0.02246 * [9.488990905082502e-83 * 0.03] + [7.991879716050322e-84 * 0.4] + [1.5152193713772794e-83 * 0.9] = 4.420223118176452e-85

T: 65

i: 1
α(65,1) = emi(1,65) * Σ[[α((65-1),1) * trans(1,1)] + [α((65-1),2) * trans(2,1)] + [α((65-1),3) * trans(3,1)]]
α(65,1) = 0.02019 * [6.071303058773428e-84 * 0.97] + [2.93247886788683e-85 * 0.0] + [4.420223118176452e-85 * 0.07] = 1.1952693062722831e-85

i: 2
α(65,2) = emi(2,65) * Σ[[α((65-1),1) * trans(1,2)] + [α((65-1),2) * trans(2,2)] + [α((65-1),3) * trans(3,2)]]
α(65,2) = 0.01597 * [6.071303058773428e-84 * 0.0] + [2.93247886788683e-85 * 0.6] + [4.420223118176452e-85 * 0.03] = 3.0216741408009943e-87

i: 3
α(65,3) = emi(3,65) * Σ[[α((65-1),1) * trans(1,3)] + [α((65-1),2) * trans(2,3)] + [α((65-1),3) * trans(3,3)]]
α(65,3) = 0.01328 * [6.071303058773428e-84 * 0.03] + [2.93247886788683e-85 * 0.4] + [4.420223118176452e-85 * 0.9] = 9.259590584081315e-87

T: 66

i: 1
α(66,1) = emi(1,66) * Σ[[α((66-1),1) * trans(1,1)] + [α((66-1),2) * trans(2,1)] + [α((66-1),3) * trans(3,1)]]
α(66,1) = 0.05171 * [1.1952693062722831e-85 * 0.97] + [3.0216741408009943e-87 * 0.0] + [9.259590584081315e-87 * 0.07] = 6.028832395289156e-87

i: 2
α(66,2) = emi(2,66) * Σ[[α((66-1),1) * trans(1,2)] + [α((66-1),2) * trans(2,2)] + [α((66-1),3) * trans(3,2)]]
α(66,2) = 0.1033 * [1.1952693062722831e-85 * 0.0] + [3.0216741408009943e-87 * 0.6] + [9.259590584081315e-87 * 0.03] = 2.159788344669136e-88

i: 3

α(66,3) = emi(3,66) * Σ[[α((66-1),1) * trans(1,3)] + [α((66-1),2) * trans(2,3)] + [α((66-1),3) * trans(3,3)]]
α(66,3) = 0.19429 * [1.1952693062722831e-85 * 0.03] + [3.0216741408009943e-87 * 0.4] + [9.259590584081315e-87 * 0.9] = 2.5506603171964584e-87

T: 67

i: 1
α(67,1) = emi(1,67) * Σ[[α((67-1),1) * trans(1,1)] + [α((67-1),2) * trans(2,1)] + [α((67-1),3) * trans(3,1)]]
α(67,1) = 0.06409 * [6.028832395289156e-87 * 0.97] + [2.159788344669136e-88 * 0.0] + [2.5506603171964584e-87 * 0.07] = 3.8623925954869795e-88

i: 2
α(67,2) = emi(2,67) * Σ[[α((67-1),1) * trans(1,2)] + [α((67-1),2) * trans(2,2)] + [α((67-1),3) * trans(3,2)]]
α(67,2) = 0.06927 * [6.028832395289156e-87 * 0.0] + [2.159788344669136e-88 * 0.6] + [2.5506603171964584e-87 * 0.03] = 1.4277039523279821e-89

i: 3
α(67,3) = emi(3,67) * Σ[[α((67-1),1) * trans(1,3)] + [α((67-1),2) * trans(2,3)] + [α((67-1),3) * trans(3,3)]]
α(67,3) = 0.04995 * [6.028832395289156e-87 * 0.03] + [2.159788344669136e-88 * 0.4] + [2.5506603171964584e-87 * 0.9] = 1.280143970165565e-88

T: 68

i: 1
α(68,1) = emi(1,68) * Σ[[α((68-1),1) * trans(1,1)] + [α((68-1),2) * trans(2,1)] + [α((68-1),3) * trans(3,1)]]
α(68,1) = 0.04294 * [3.8623925954869795e-88 * 0.97] + [1.4277039523279821e-89 * 0.0] + [1.280143970165565e-88 * 0.07] = 1.6472346065422823e-89

i: 2
α(68,2) = emi(2,68) * Σ[[α((68-1),1) * trans(1,2)] + [α((68-1),2) * trans(2,2)] + [α((68-1),3) * trans(3,2)]]
α(68,2) = 0.04257 * [3.8623925954869795e-88 * 0.0] + [1.4277039523279821e-89 * 0.6] + [1.280143970165565e-88 * 0.03] = 5.281513299334574e-91

i: 3
α(68,3) = emi(3,68) * Σ[[α((68-1),1) * trans(1,3)] + [α((68-1),2) * trans(2,3)] + [α((68-1),3) * trans(3,3)]]
α(68,3) = 0.11935 * [3.8623925954869795e-88 * 0.03] + [1.4277039523279821e-89 * 0.4] + [1.280143970165565e-88 * 0.9] = 1.581518199118891e-89

T: 69

i: 1
α(69,1) = emi(1,69) * Σ[[α((69-1),1) * trans(1,1)] + [α((69-1),2) * trans(2,1)] + [α((69-1),3) * trans(3,1)]]
α(69,1) = 0.05171 * [1.6472346065422823e-89 * 0.97] + [5.281513299334574e-91 * 0.0] + [1.581518199118891e-89 * 0.07] = 8.834776788452302e-91

i: 2
α(69,2) = emi(2,69) * Σ[[α((69-1),1) * trans(1,2)] + [α((69-1),2) * trans(2,2)] + [α((69-1),3) * trans(3,2)]]
α(69,2) = 0.1033 * [1.6472346065422823e-89 * 0.0] + [5.281513299334574e-91 * 0.6] + [1.581518199118891e-89 * 0.03] = 8.174606841997013e-92

i: 3
α(69,3) = emi(3,69) * Σ[[α((69-1),1) * trans(1,3)] + [α((69-1),2) * trans(2,3)] + [α((69-1),3) * trans(3,3)]]
α(69,3) = 0.19429 * [1.6472346065422823e-89 * 0.03] + [5.281513299334574e-91 * 0.4] + [1.581518199118891e-89 * 0.9] = 2.9025167104299228e-90

T: 70

i: 1
α(70,1) = emi(1,70) * Σ[[α((70-1),1) * trans(1,1)] + [α((70-1),2) * trans(2,1)] + [α((70-1),3) * trans(3,1)]]
α(70,1) = 0.02253 * [8.834776788452302e-91 * 0.97] + [8.174606841997013e-92 * 0.0] + [2.9025167104299228e-90 * 0.07] = 2.388516864527058e-92

i: 2
α(70,2) = emi(2,70) * Σ[[α((70-1),1) * trans(1,2)] + [α((70-1),2) * trans(2,2)] + [α((70-1),3) * trans(3,2)]]
α(70,2) = 0.02129 * [8.834776788452302e-91 * 0.0] + [8.174606841997013e-92 * 0.6] + [2.9025167104299228e-90 * 0.03] = 2.89806170094829e-93

i: 3
α(70,3) = emi(3,70) * Σ[[α((70-1),1) * trans(1,3)] + [α((70-1),2) * trans(2,3)] + [α((70-1),3) * trans(3,3)]]
α(70,3) = 0.00782 * [8.834776788452302e-91 * 0.03] + [8.174606841997013e-92 * 0.4] + [2.9025167104299228e-90 * 0.9] = 2.0890878173480557e-92

T: 71

i: 1
α(71,1) = emi(1,71) * Σ[[α((71-1),1) * trans(1,1)] + [α((71-1),2) * trans(2,1)] + [α((71-1),3) * trans(3,1)]]
α(71,1) = 0.05171 * [2.388516864527058e-92 * 0.97] + [2.89806170094829e-93 * 0.0] + [2.0890878173480557e-92 * 0.07] = 1.273667720252081e-93

i: 2
α(71,2) = emi(2,71) * Σ[[α((71-1),1) * trans(1,2)] + [α((71-1),2) * trans(2,2)] + [α((71-1),3) * trans(3,2)]]
α(71,2) = 0.1033 * [2.388516864527058e-92 * 0.0] + [2.89806170094829e-93 * 0.6] + [2.0890878173480557e-92 * 0.03] = 2.4436269568439127e-94

i: 3
α(71,3) = emi(3,71) * Σ[[α((71-1),1) * trans(1,3)] + [α((71-1),2) * trans(2,3)] + [α((71-1),3) * trans(3,3)]]
α(71,3) = 0.19429 * [2.388516864527058e-92 * 0.03] + [2.89806170094829e-93 * 0.4] + [2.0890878173480557e-92 * 0.9] = 4.01744509392657e-93

T: 72

i: 1
α(72,1) = emi(1,72) * Σ[[α((72-1),1) * trans(1,1)] + [α((72-1),2) * trans(2,1)] + [α((72-1),3) * trans(3,1)]]
α(72,1) = 0.05092 * [1.273667720252081e-93 * 0.97] + [2.4436269568439127e-94 * 0.0] + [4.01744509392657e-93 * 0.07] = 7.722928679857076e-95

i: 2
α(72,2) = emi(2,72) * Σ[[α((72-1),1) * trans(1,2)] + [α((72-1),2) * trans(2,2)] + [α((72-1),3) * trans(3,2)]]
α(72,2) = 0.03962 * [1.273667720252081e-93 * 0.0] + [2.4436269568439127e-94 * 0.6] + [4.01744509392657e-93 * 0.03] = 1.058412524045047e-95

i: 3
α(72,3) = emi(3,72) * Σ[[α((72-1),1) * trans(1,3)] + [α((72-1),2) * trans(2,3)] + [α((72-1),3) * trans(3,3)]]
α(72,3) = 0.00687 * [1.273667720252081e-93 * 0.03] + [2.4436269568439127e-94 * 0.4] + [4.01744509392657e-93 * 0.9] = 2.5773874620632647e-95

T: 73

i: 1
α(73,1) = emi(1,73) * Σ[[α((73-1),1) * trans(1,1)] + [α((73-1),2) * trans(2,1)] + [α((73-1),3) * trans(3,1)]]
α(73,1) = 0.04621 * [7.722928679857076e-95 * 0.97] + [1.058412524045047e-95 * 0.0] + [2.5773874620632647e-95 * 0.07] = 3.545073134908456e-96

i: 2
α(73,2) = emi(2,73) * Σ[[α((73-1),1) * trans(1,2)] + [α((73-1),2) * trans(2,2)] + [α((73-1),3) * trans(3,2)]]
α(73,2) = 0.05409 * [7.722928679857076e-95 * 0.0] + [1.058412524045047e-95 * 0.6] + [2.5773874620632647e-95 * 0.03] = 3.853204669004801e-97

i: 3
α(73,3) = emi(3,73) * Σ[[α((73-1),1) * trans(1,3)] + [α((73-1),2) * trans(2,3)] + [α((73-1),3) * trans(3,3)]]
α(73,3) = 0.00457 * [7.722928679857076e-95 * 0.03] + [1.058412524045047e-95 * 0.4] + [2.5773874620632647e-95 * 0.9] = 1.359438624742896e-97

T: 74

i: 1
α(74,1) = emi(1,74) * Σ[[α((74-1),1) * trans(1,1)] + [α((74-1),2) * trans(2,1)] + [α((74-1),3) * trans(3,1)]]
α(74,1) = 0.09042 * [3.545073134908456e-96 * 0.97] + [3.853204669004801e-97 * 0.0] + [1.359438624742896e-97 * 0.07] = 3.117895905558147e-97

i: 2
α(74,2) = emi(2,74) * Σ[[α((74-1),1) * trans(1,2)] + [α((74-1),2) * trans(2,2)] + [α((74-1),3) * trans(3,2)]]
α(74,2) = 0.05638 * [3.545073134908456e-96 * 0.0] + [3.853204669004801e-97 * 0.6] + [1.359438624742896e-97 * 0.03] = 1.3264556203298453e-98

i: 3
α(74,3) = emi(3,74) * Σ[[α((74-1),1) * trans(1,3)] + [α((74-1),2) * trans(2,3)] + [α((74-1),3) * trans(3,3)]]
α(74,3) = 0.00398 * [3.545073134908456e-96 * 0.03] + [3.853204669004801e-97 * 0.4] + [1.359438624742896e-97 * 0.9] = 1.5236628309965392e-99

T: 75

i: 1
α(75,1) = emi(1,75) * Σ[[α((75-1),1) * trans(1,1)] + [α((75-1),2) * trans(2,1)] + [α((75-1),3) * trans(3,1)]]
α(75,1) = 0.05877 * [3.117895905558147e-97 * 0.97] + [1.3264556203298453e-98 * 0.0] + [1.5236628309965392e-99 * 0.07] = 1.7780426206376708e-98

i: 2
α(75,2) = emi(2,75) * Σ[[α((75-1),1) * trans(1,2)] + [α((75-1),2) * trans(2,2)] + [α((75-1),3) * trans(3,2)]]
α(75,2) = 0.04271 * [3.117895905558147e-97 * 0.0] + [1.3264556203298453e-98 * 0.6] + [1.5236628309965392e-99 * 0.03] = 3.41869786451082e-100

i: 3
α(75,3) = emi(3,75) * Σ[[α((75-1),1) * trans(1,3)] + [α((75-1),2) * trans(2,3)] + [α((75-1),3) * trans(3,3)]]
α(75,3) = 0.01879 * [3.117895905558147e-97 * 0.03] + [1.3264556203298453e-98 * 0.4] + [1.5236628309965392e-99 * 0.9] = 3.0121885875528645e-100

T: 76

i: 1
α(76,1) = emi(1,76) * Σ[[α((76-1),1) * trans(1,1)] + [α((76-1),2) * trans(2,1)] + [α((76-1),3) * trans(3,1)]]
α(76,1) = 0.01482 * [1.7780426206376708e-98 * 0.97] + [3.41869786451082e-100 * 0.0] + [3.0121885875528645e-100 * 0.07] = 2.5591322333122044e-100

i: 2
α(76,2) = emi(2,76) * Σ[[α((76-1),1) * trans(1,2)] + [α((76-1),2) * trans(2,2)] + [α((76-1),3) * trans(3,2)]]
α(76,2) = 0.01439 * [1.7780426206376708e-98 * 0.0] + [3.41869786451082e-100 * 0.6] + [3.0121885875528645e-100 * 0.03] = 3.081739917543299e-102

i: 3
α(76,3) = emi(3,76) * Σ[[α((76-1),1) * trans(1,3)] + [α((76-1),2) * trans(2,3)] + [α((76-1),3) * trans(3,3)]]
α(76,3) = 0.04444 * [1.7780426206376708e-98 * 0.03] + [3.41869786451082e-100 * 0.4] + [3.0121885875528645e-100 * 0.9] = 4.18294910170723e-101

T: 77

i: 1
α(77,1) = emi(1,77) * Σ[[α((77-1),1) * trans(1,1)] + [α((77-1),2) * trans(2,1)] + [α((77-1),3) * trans(3,1)]]
α(77,1) = 0.06409 * [2.5591322333122044e-100 * 0.97] + [3.081739917543299e-102 * 0.0] + [4.18294910170723e-101 * 0.07] = 1.609709377434887e-101

i: 2
α(77,2) = emi(2,77) * Σ[[α((77-1),1) * trans(1,2)] + [α((77-1),2) * trans(2,2)] + [α((77-1),3) * trans(3,2)]]
α(77,2) = 0.06927 * [2.5591322333122044e-100 * 0.0] + [3.081739917543299e-102 * 0.6] + [4.18294910170723e-101 * 0.03] = 2.150091397355125e-103

i: 3
α(77,3) = emi(3,77) * Σ[[α((77-1),1) * trans(1,3)] + [α((77-1),2) * trans(2,3)] + [α((77-1),3) * trans(3,3)]]
α(77,3) = 0.04995 * [2.5591322333122044e-100 * 0.03] + [3.081739917543299e-102 * 0.4] + [4.18294910170723e-101 * 0.9] = 2.3255038973868345e-102

T: 78

i: 1
α(78,1) = emi(1,78) * Σ[[α((78-1),1) * trans(1,1)] + [α((78-1),2) * trans(2,1)] + [α((78-1),3) * trans(3,1)]]
α(78,1) = 0.06901 * [1.609709377434887e-101 * 0.97] + [2.150091397355125e-103 * 0.0] + [2.3255038973868345e-102 * 0.07] = 1.0887684398038875e-102

i: 2
α(78,2) = emi(2,78) * Σ[[α((78-1),1) * trans(1,2)] + [α((78-1),2) * trans(2,2)] + [α((78-1),3) * trans(3,2)]]
α(78,2) = 0.07052 * [1.609709377434887e-101 * 0.0] + [2.150091397355125e-103 * 0.6] + [2.3255038973868345e-102 * 0.03] = 1.401730276580059e-104

i: 3
α(78,3) = emi(3,78) * Σ[[α((78-1),1) * trans(1,3)] + [α((78-1),2) * trans(2,3)] + [α((78-1),3) * trans(3,3)]]
α(78,3) = 0.09536 * [1.609709377434887e-101 * 0.03] + [2.150091397355125e-103 * 0.4] + [2.3255038973868345e-102 * 0.9] = 2.5383592098505634e-103

T: 79

i: 1
α(79,1) = emi(1,79) * Σ[[α((79-1),1) * trans(1,1)] + [α((79-1),2) * trans(2,1)] + [α((79-1),3) * trans(3,1)]]
α(79,1) = 0.05171 * [1.0887684398038875e-102 * 0.97] + [1.401730276580059e-104 * 0.0] + [2.5383592098505634e-103 * 0.07] = 5.553001942478085e-104

i: 2
α(79,2) = emi(2,79) * Σ[[α((79-1),1) * trans(1,2)] + [α((79-1),2) * trans(2,2)] + [α((79-1),3) * trans(3,2)]]
α(79,2) = 0.1033 * [1.0887684398038875e-102 * 0.0] + [1.401730276580059e-104 * 0.6] + [2.5383592098505634e-103 * 0.03] = 1.6554299445570102e-105

i: 3
α(79,3) = emi(3,79) * Σ[[α((79-1),1) * trans(1,3)] + [α((79-1),2) * trans(2,3)] + [α((79-1),3) * trans(3,3)]]
α(79,3) = 0.19429 * [1.0887684398038875e-102 * 0.03] + [1.401730276580059e-104 * 0.4] + [2.5383592098505634e-103 * 0.9] = 5.182147628619981e-104

T: 80

i: 1
α(80,1) = emi(1,80) * Σ[[α((80-1),1) * trans(1,1)] + [α((80-1),2) * trans(2,1)] + [α((80-1),3) * trans(3,1)]]
α(80,1) = 0.05171 * [5.553001942478085e-104 * 0.97] + [1.6554299445570102e-105 * 0.0] + [5.182147628619981e-104 * 0.07] = 2.9728917830349125e-105

i: 2
α(80,2) = emi(2,80) * Σ[[α((80-1),1) * trans(1,2)] + [α((80-1),2) * trans(2,2)] + [α((80-1),3) * trans(3,2)]]
α(80,2) = 0.1033 * [5.553001942478085e-104 * 0.0] + [1.6554299445570102e-105 * 0.6] + [5.182147628619981e-104 * 0.03] = 2.6319830297457673e-106

i: 3
α(80,3) = emi(3,80) * Σ[[α((80-1),1) * trans(1,3)] + [α((80-1),2) * trans(2,3)] + [α((80-1),3) * trans(3,3)]]
α(80,3) = 0.19429 * [5.553001942478085e-104 * 0.03] + [1.6554299445570102e-105 * 0.4] + [5.182147628619981e-104 * 0.9] = 9.513876382673597e-105

T: 81

i: 1
α(81,1) = emi(1,81) * Σ[[α((81-1),1) * trans(1,1)] + [α((81-1),2) * trans(2,1)] + [α((81-1),3) * trans(3,1)]]
α(81,1) = 0.06521 * [2.9728917830349125e-105 * 0.97] + [2.6319830297457673e-106 * 0.0] + [9.513876382673597e-105 * 0.07] = 2.3147439650054565e-106

i: 2
α(81,2) = emi(2,81) * Σ[[α((81-1),1) * trans(1,2)] + [α((81-1),2) * trans(2,2)] + [α((81-1),3) * trans(3,2)]]
α(81,2) = 0.05586 * [2.9728917830349125e-105 * 0.0] + [2.6319830297457673e-106 * 0.6] + [9.513876382673597e-105 * 0.03] = 2.476470836458033e-107

i: 3
α(81,3) = emi(3,81) * Σ[[α((81-1),1) * trans(1,3)] + [α((81-1),2) * trans(2,3)] + [α((81-1),3) * trans(3,3)]]
α(81,3) = 0.02246 * [2.9728917830349125e-105 * 0.03] + [2.6319830297457673e-106 * 0.4] + [9.513876382673597e-105 * 0.9] = 1.9668120523669663e-106

T: 82

i: 1
α(82,1) = emi(1,82) * Σ[[α((82-1),1) * trans(1,1)] + [α((82-1),2) * trans(2,1)] + [α((82-1),3) * trans(3,1)]]
α(82,1) = 0.06901 * [2.3147439650054565e-106 * 0.97] + [2.476470836458033e-107 * 0.0] + [1.9668120523669663e-106 * 0.07] = 1.6444934557564487e-107

i: 2
α(82,2) = emi(2,82) * Σ[[α((82-1),1) * trans(1,2)] + [α((82-1),2) * trans(2,2)] + [α((82-1),3) * trans(3,2)]]
α(82,2) = 0.07052 * [2.3147439650054565e-106 * 0.0] + [2.476470836458033e-107 * 0.6] + [1.9668120523669663e-106 * 0.03] = 1.4639430981208782e-108

i: 3
α(82,3) = emi(3,82) * Σ[[α((82-1),1) * trans(1,3)] + [α((82-1),2) * trans(2,3)] + [α((82-1),3) * trans(3,3)]]
α(82,3) = 0.09536 * [2.3147439650054565e-106 * 0.03] + [2.476470836458033e-107 * 0.4] + [1.9668120523669663e-106 * 0.9] = 1.8486794747601562e-107

T: 83

i: 1
α(83,1) = emi(1,83) * Σ[[α((83-1),1) * trans(1,1)] + [α((83-1),2) * trans(2,1)] + [α((83-1),3) * trans(3,1)]]
α(83,1) = 0.04595 * [1.6444934557564487e-107 * 0.97] + [1.4639430981208782e-108 * 0.0] + [1.8486794747601562e-107 * 0.07] = 7.924381759381458e-109

i: 2
α(83,2) = emi(2,83) * Σ[[α((83-1),1) * trans(1,2)] + [α((83-1),2) * trans(2,2)] + [α((83-1),3) * trans(3,2)]]
α(83,2) = 0.04801 * [1.6444934557564487e-107 * 0.0] + [1.4639430981208782e-108 * 0.6] + [1.8486794747601562e-107 * 0.03] = 6.879687535944054e-110

i: 3
α(83,3) = emi(3,83) * Σ[[α((83-1),1) * trans(1,3)] + [α((83-1),2) * trans(2,3)] + [α((83-1),3) * trans(3,3)]]
α(83,3) = 0.14222 * [1.6444934557564487e-107 * 0.03] + [1.4639430981208782e-108 * 0.4] + [1.8486794747601562e-107 * 0.9] = 2.5197175068527103e-108

T: 84

i: 1
α(84,1) = emi(1,84) * Σ[[α((84-1),1) * trans(1,1)] + [α((84-1),2) * trans(2,1)] + [α((84-1),3) * trans(3,1)]]
α(84,1) = 0.04595 * [7.924381759381458e-109 * 0.97] + [6.879687535944054e-110 * 0.0] + [2.5197175068527103e-108 * 0.07] = 4.34248295196188e-110

i: 2
α(84,2) = emi(2,84) * Σ[[α((84-1),1) * trans(1,2)] + [α((84-1),2) * trans(2,2)] + [α((84-1),3) * trans(3,2)]]

α(84,2) = 0.04801 * [7.924381759381458e-109 * 0.0] + [6.879687535944054e-110 * 0.6] + [2.5197175068527103e-108 * 0.03] = 5.610911916724003e-111

i: 3
α(84,3) = emi(3,84) * Σ[[α((84-1),1) * trans(1,3)] + [α((84-1),2) * trans(2,3)] + [α((84-1),3) * trans(3,3)]]
α(84,3) = 0.14222 * [7.924381759381458e-109 * 0.03] + [6.879687535944054e-110 * 0.4] + [2.5197175068527103e-108 * 0.9] = 3.2981353480903877e-109

T: 85

i: 1
α(85,1) = emi(1,85) * Σ[[α((85-1),1) * trans(1,1)] + [α((85-1),2) * trans(2,1)] + [α((85-1),3) * trans(3,1)]]
α(85,1) = 0.04294 * [4.34248295196188e-110 * 0.97] + [5.610911916724003e-111 * 0.0] + [3.2981353480903877e-109 * 0.07] = 2.8000758371142673e-111

i: 2
α(85,2) = emi(2,85) * Σ[[α((85-1),1) * trans(1,2)] + [α((85-1),2) * trans(2,2)] + [α((85-1),3) * trans(3,2)]]
α(85,2) = 0.04257 * [4.34248295196188e-110 * 0.0] + [5.610911916724003e-111 * 0.6] + [3.2981353480903877e-109 * 0.03] = 5.645187774815878e-112

i: 3
α(85,3) = emi(3,85) * Σ[[α((85-1),1) * trans(1,3)] + [α((85-1),2) * trans(2,3)] + [α((85-1),3) * trans(3,3)]]
α(85,3) = 0.11935 * [4.34248295196188e-110 * 0.03] + [5.610911916724003e-111 * 0.4] + [3.2981353480903877e-109 * 0.9] = 3.58502683785123e-110

T: 86

i: 1
α(86,1) = emi(1,86) * Σ[[α((86-1),1) * trans(1,1)] + [α((86-1),2) * trans(2,1)] + [α((86-1),3) * trans(3,1)]]
α(86,1) = 0.04294 * [2.8000758371142673e-111 * 0.97] + [5.645187774815878e-112 * 0.0] + [3.58502683785123e-110 * 0.07] = 2.243869354444483e-112

i: 2
α(86,2) = emi(2,86) * Σ[[α((86-1),1) * trans(1,2)] + [α((86-1),2) * trans(2,2)] + [α((86-1),3) * trans(3,2)]]
α(86,2) = 0.04257 * [2.8000758371142673e-111 * 0.0] + [5.645187774815878e-112 * 0.6] + [3.58502683785123e-110 * 0.03] = 6.020331636063278e-113

i: 3
α(86,3) = emi(3,86) * Σ[[α((86-1),1) * trans(1,3)] + [α((86-1),2) * trans(2,3)] + [α((86-1),3) * trans(3,3)]]
α(86,3) = 0.11935 * [2.8000758371142673e-111 * 0.03] + [5.645187774815878e-112 * 0.4] + [3.58502683785123e-110 * 0.9] = 3.887832375849657e-111

T: 87

i: 1
α(87,1) = emi(1,87) * Σ[[α((87-1),1) * trans(1,1)] + [α((87-1),2) * trans(2,1)] + [α((87-1),3) * trans(3,1)]]
α(87,1) = 0.05171 * [2.243869354444483e-112 * 0.97] + [6.020331636063278e-113 * 0.0] + [3.887832375849657e-111 * 0.07] = 2.5327743829740455e-113

i: 2
α(87,2) = emi(2,87) * Σ[[α((87-1),1) * trans(1,2)] + [α((87-1),2) * trans(2,2)] + [α((87-1),3) * trans(3,2)]]
α(87,2) = 0.1033 * [2.243869354444483e-112 * 0.0] + [6.020331636063278e-113 * 0.6] + [3.887832375849657e-111 * 0.03] = 1.5779794080790107e-113

i: 3
α(87,3) = emi(3,87) * Σ[[α((87-1),1) * trans(1,3)] + [α((87-1),2) * trans(2,3)] + [α((87-1),3) * trans(3,3)]]
α(87,3) = 0.19429 * [2.243869354444483e-112 * 0.03] + [6.020331636063278e-113 * 0.4] + [3.887832375849657e-111 * 0.9] = 6.858169021383549e-112

T: 88

i: 1
α(88,1) = emi(1,88) * Σ[[α((88-1),1) * trans(1,1)] + [α((88-1),2) * trans(2,1)] + [α((88-1),3) * trans(3,1)]]
α(88,1) = 0.06521 * [2.5327743829740455e-113 * 0.97] + [1.5779794080790107e-113 * 0.0] + [6.858169021383549e-112 * 0.07] = 4.732621923074203e-114

i: 2
α(88,2) = emi(2,88) * Σ[[α((88-1),1) * trans(1,2)] + [α((88-1),2) * trans(2,2)] + [α((88-1),3) * trans(3,2)]]
α(88,2) = 0.05586 * [2.5327743829740455e-113 * 0.0] + [1.5779794080790107e-113 * 0.6] + [6.858169021383549e-112 * 0.03] = 1.6781675430152162e-114

i: 3
α(88,3) = emi(3,88) * Σ[[α((88-1),1) * trans(1,3)] + [α((88-1),2) * trans(2,3)] + [α((88-1),3) * trans(3,3)]]
α(88,3) = 0.02246 * [2.5327743829740455e-113 * 0.03] + [1.5779794080790107e-113 * 0.4] + [6.858169021383549e-112 * 0.9] = 1.4021934363639005e-113

T: 89

i: 1
α(89,1) = emi(1,89) * Σ[[α((89-1),1) * trans(1,1)] + [α((89-1),2) * trans(2,1)] + [α((89-1),3) * trans(3,1)]]
α(89,1) = 0.04294 * [4.732621923074203e-114 * 0.97] + [1.6781675430152162e-114 * 0.0] + [1.4021934363639005e-113 * 0.07] = 2.3926935212572825e-115

i: 2
α(89,2) = emi(2,89) * Σ[[α((89-1),1) * trans(1,2)] + [α((89-1),2) * trans(2,2)] + [α((89-1),3) * trans(3,2)]]
α(89,2) = 0.04257 * [4.732621923074203e-114 * 0.0] + [1.6781675430152162e-114 * 0.6] + [1.4021934363639005e-113 * 0.03] = 6.077116775949802e-116

i: 3
α(89,3) = emi(3,89) * Σ[[α((89-1),1) * trans(1,3)] + [α((89-1),2) * trans(2,3)] + [α((89-1),3) * trans(3,3)]]
α(89,3) = 0.11935 * [4.732621923074203e-114 * 0.03] + [1.6781675430152162e-114 * 0.4] + [1.4021934363639005e-113 * 0.9] = 1.6032269509693975e-114

T: 90

i: 1
α(90,1) = emi(1,90) * Σ[[α((90-1),1) * trans(1,1)] + [α((90-1),2) * trans(2,1)] + [α((90-1),3) * trans(3,1)]]
α(90,1) = 0.06901 * [2.3926935212572825e-115 * 0.97] + [6.077116775949802e-116 * 0.0] + [1.6032269509693975e-114 * 0.07] = 2.376132708253848e-116

i: 2

α(90,2) = emi(2,90) * Σ[[α((90-1),1) * trans(1,2)] + [α((90-1),2) * trans(2,2)] + [α((90-1),3) * trans(3,2)]]
α(90,2) = 0.07052 * [2.3926935212572825e-115 * 0.0] + [6.077116775949802e-116 * 0.6] + [1.6032269509693975e-114 * 0.03] = 5.963136587710737e-117

i: 3
α(90,3) = emi(3,90) * Σ[[α((90-1),1) * trans(1,3)] + [α((90-1),2) * trans(2,3)] + [α((90-1),3) * trans(3,3)]]
α(90,3) = 0.09536 * [2.3926935212572825e-115 * 0.03] + [6.077116775949802e-116 * 0.4] + [1.6032269509693975e-114 * 0.9] = 1.4059790702557715e-115

T: 91

i: 1
α(91,1) = emi(1,91) * Σ[[α((91-1),1) * trans(1,1)] + [α((91-1),2) * trans(2,1)] + [α((91-1),3) * trans(3,1)]]
α(91,1) = 0.05591 * [2.376132708253848e-116 * 0.97] + [5.963136587710737e-117 * 0.0] + [1.4059790702557715e-115 * 0.07] = 1.838898951995186e-117

i: 2
α(91,2) = emi(2,91) * Σ[[α((91-1),1) * trans(1,2)] + [α((91-1),2) * trans(2,2)] + [α((91-1),3) * trans(3,2)]]
α(91,2) = 0.07239 * [2.376132708253848e-116 * 0.0] + [5.963136587710737e-117 * 0.6] + [1.4059790702557715e-115 * 0.03] = 5.64339349238074e-118

i: 3
α(91,3) = emi(3,91) * Σ[[α((91-1),1) * trans(1,3)] + [α((91-1),2) * trans(2,3)] + [α((91-1),3) * trans(3,3)]]
α(91,3) = 0.06898 * [2.376132708253848e-116 * 0.03] + [5.963136587710737e-117 * 0.4] + [1.4059790702557715e-115 * 0.9] = 8.9423058189546e-117

T: 92

i: 1
α(92,1) = emi(1,92) * Σ[[α((92-1),1) * trans(1,1)] + [α((92-1),2) * trans(2,1)] + [α((92-1),3) * trans(3,1)]]
α(92,1) = 0.05877 * [1.838898951995186e-117 * 0.97] + [5.64339349238074e-118 * 0.0] + [8.9423058189546e-117 * 0.07] = 1.4161768057509168e-118

i: 2
α(92,2) = emi(2,92) * Σ[[α((92-1),1) * trans(1,2)] + [α((92-1),2) * trans(2,2)] + [α((92-1),3) * trans(3,2)]]
α(92,2) = 0.04271 * [1.838898951995186e-117 * 0.0] + [5.64339349238074e-118 * 0.6] + [8.9423058189546e-117 * 0.03] = 2.591953660940141e-119

i: 3
α(92,3) = emi(3,92) * Σ[[α((92-1),1) * trans(1,3)] + [α((92-1),2) * trans(2,3)] + [α((92-1),3) * trans(3,3)]]
α(92,3) = 0.01879 * [1.838898951995186e-117 * 0.03] + [5.64339349238074e-118 * 0.4] + [8.9423058189546e-117 * 0.9] = 1.565014955924543e-118

T: 93

i: 1
α(93,1) = emi(1,93) * Σ[[α((93-1),1) * trans(1,1)] + [α((93-1),2) * trans(2,1)] + [α((93-1),3) * trans(3,1)]]
α(93,1) = 0.04294 * [1.4161768057509168e-118 * 0.97] + [2.591953660940141e-119 * 0.0] + [1.565014955924543e-118 * 0.07] = 6.369043503229403e-120

i: 2
α(93,2) = emi(2,93) * Σ[[α((93-1),1) * trans(1,2)] + [α((93-1),2) * trans(2,2)] + [α((93-1),3) * trans(3,2)]]
α(93,2) = 0.04257 * [1.4161768057509168e-118 * 0.0] + [2.591953660940141e-119 * 0.6] + [1.565014955924543e-118 * 0.03] = 8.61904864098454e-121

i: 3
α(93,3) = emi(3,93) * Σ[[α((93-1),1) * trans(1,3)] + [α((93-1),2) * trans(2,3)] + [α((93-1),3) * trans(3,3)]]
α(93,3) = 0.11935 * [1.4161768057509168e-118 * 0.03] + [2.591953660940141e-119 * 0.4] + [1.565014955924543e-118 * 0.9] = 1.8555068932095417e-119

T: 94

i: 1
α(94,1) = emi(1,94) * Σ[[α((94-1),1) * trans(1,1)] + [α((94-1),2) * trans(2,1)] + [α((94-1),3) * trans(3,1)]]
α(94,1) = 0.05171 * [6.369043503229403e-120 * 0.97] + [8.61904864098454e-121 * 0.0] + [1.8555068932095417e-119 * 0.07] = 3.866267253789384e-121

i: 2
α(94,2) = emi(2,94) * Σ[[α((94-1),1) * trans(1,2)] + [α((94-1),2) * trans(2,2)] + [α((94-1),3) * trans(3,2)]]
α(94,2) = 0.1033 * [6.369043503229403e-120 * 0.0] + [8.61904864098454e-121 * 0.6] + [1.8555068932095417e-119 * 0.03] = 1.109230220973859e-121

i: 3
α(94,3) = emi(3,94) * Σ[[α((94-1),1) * trans(1,3)] + [α((94-1),2) * trans(2,3)] + [α((94-1),3) * trans(3,3)]]
α(94,3) = 0.19429 * [6.369043503229403e-120 * 0.03] + [8.61904864098454e-121 * 0.4] + [1.8555068932095417e-119 * 0.9] = 3.348664950820685e-120

T: 95

i: 1
α(95,1) = emi(1,95) * Σ[[α((95-1),1) * trans(1,1)] + [α((95-1),2) * trans(2,1)] + [α((95-1),3) * trans(3,1)]]
α(95,1) = 0.04595 * [3.866267253789384e-121 * 0.97] + [1.109230220973859e-121 * 0.0] + [3.348664950820685e-120 * 0.07] = 2.8003513904542087e-122

i: 2
α(95,2) = emi(2,95) * Σ[[α((95-1),1) * trans(1,2)] + [α((95-1),2) * trans(2,2)] + [α((95-1),3) * trans(3,2)]]
α(95,2) = 0.04801 * [3.866267253789384e-121 * 0.0] + [1.109230220973859e-121 * 0.6] + [3.348664950820685e-120 * 0.03] = 8.018330703204329e-123

i: 3
α(95,3) = emi(3,95) * Σ[[α((95-1),1) * trans(1,3)] + [α((95-1),2) * trans(2,3)] + [α((95-1),3) * trans(3,3)]]
α(95,3) = 0.14222 * [3.866267253789384e-121 * 0.03] + [1.109230220973859e-121 * 0.4] + [3.348664950820685e-120 * 0.9] = 4.36582186842724e-121

T: 96

i: 1
α(96,1) = emi(1,96) * Σ[[α((96-1),1) * trans(1,1)] + [α((96-1),2) * trans(2,1)] + [α((96-1),3) * trans(3,1)]]
α(96,1) = 0.04294 * [2.8003513904542087e-122 * 0.97] + [8.018330703204329e-123 * 0.0] + [4.36582186842724e-121 * 0.07] = 2.478675497661066e-123

i: 2
α(96,2) = emi(2,96) * Σ[[α((96-1),1) * trans(1,2)] + [α((96-1),2) * trans(2,2)] + [α((96-1),3) * trans(3,2)]]
α(96,2) = 0.04257 * [2.8003513904542087e-122 * 0.0] + [8.018330703204329e-123 * 0.6] + [4.36582186842724e-121 * 0.03] = 7.623633136380878e-124

i: 3
α(96,3) = emi(3,96) * Σ[[α((96-1),1) * trans(1,3)] + [α((96-1),2) * trans(2,3)] + [α((96-1),3) * trans(3,3)]]
α(96,3) = 0.11935 * [2.8003513904542087e-122 * 0.03] + [8.018330703204329e-123 * 0.4] + [4.36582186842724e-121 * 0.9] = 4.737853728901738e-122

T: 97

i: 1
α(97,1) = emi(1,97) * Σ[[α((97-1),1) * trans(1,1)] + [α((97-1),2) * trans(2,1)] + [α((97-1),3) * trans(3,1)]]
α(97,1) = 0.0421 * [2.478675497661066e-123 * 0.97] + [7.623633136380878e-124 * 0.0] + [4.737853728901738e-122 * 0.07] = 2.408462206887192e-124

i: 2
α(97,2) = emi(2,97) * Σ[[α((97-1),1) * trans(1,2)] + [α((97-1),2) * trans(2,2)] + [α((97-1),3) * trans(3,2)]]
α(97,2) = 0.0408 * [2.478675497661066e-123 * 0.0] + [7.623633136380878e-124 * 0.6] + [4.737853728901738e-122 * 0.03] = 7.665398355961766e-125

i: 3
α(97,3) = emi(3,97) * Σ[[α((97-1),1) * trans(1,3)] + [α((97-1),2) * trans(2,3)] + [α((97-1),3) * trans(3,3)]]
α(97,3) = 0.02568 * [2.478675497661066e-123 * 0.03] + [7.623633136380878e-124 * 0.4] + [4.737853728901738e-122 * 0.9] = 1.1047533213848584e-123

T: 98

i: 1
α(98,1) = emi(1,98) * Σ[[α((98-1),1) * trans(1,1)] + [α((98-1),2) * trans(2,1)] + [α((98-1),3) * trans(3,1)]]
α(98,1) = 0.06901 * [2.408462206887192e-124 * 0.97] + [7.665398355961766e-125 * 0.0] + [1.1047533213848584e-123 * 0.07] = 2.1458905628650492e-125

i: 2
α(98,2) = emi(2,98) * Σ[[α((98-1),1) * trans(1,2)] + [α((98-1),2) * trans(2,2)] + [α((98-1),3) * trans(3,2)]]
α(98,2) = 0.07052 * [2.408462206887192e-124 * 0.0] + [7.665398355961766e-125 * 0.6] + [1.1047533213848584e-123 * 0.03] = 5.5805994790963485e-126

i: 3
α(98,3) = emi(3,98) * Σ[[α((98-1),1) * trans(1,3)] + [α((98-1),2) * trans(2,3)] + [α((98-1),3) * trans(3,3)]]
α(98,3) = 0.09536 * [2.408462206887192e-124 * 0.03] + [7.665398355961766e-125 * 0.4] + [1.1047533213848584e-123 * 0.9] = 9.842725147157843e-125

T: 99

i: 1
α(99,1) = emi(1,99) * Σ[[α((99-1),1) * trans(1,1)] + [α((99-1),2) * trans(2,1)] + [α((99-1),3) * trans(3,1)]]
α(99,1) = 0.04595 * [2.1458905628650492e-125 * 0.97] + [5.5805994790963485e-126 * 0.0] + [9.842725147157843e-125 * 0.07] = 1.2730468665857275e-126

i: 2
α(99,2) = emi(2,99) * Σ[[α((99-1),1) * trans(1,2)] + [α((99-1),2) * trans(2,2)] + [α((99-1),3) * trans(3,2)]]
α(99,2) = 0.04801 * [2.1458905628650492e-125 * 0.0] + [5.5805994790963485e-126 * 0.6] + [9.842725147157843e-125 * 0.03] = 3.025195188893638e-127

i: 3
α(99,3) = emi(3,99) * Σ[[α((99-1),1) * trans(1,3)] + [α((99-1),2) * trans(2,3)] + [α((99-1),3) * trans(3,3)]]
α(99,3) = 0.14222 * [2.1458905628650492e-125 * 0.03] + [5.5805994790963485e-126 * 0.4] + [9.842725147157843e-125 * 0.9] = 1.300751704378113e-125

T: 100

i: 1
α(100,1) = emi(1,100) * Σ[[α((100-1),1) * trans(1,1)] + [α((100-1),2) * trans(2,1)] + [α((100-1),3) * trans(3,1)]]
α(100,1) = 0.06409 * [1.2730468665857275e-126 * 0.97] + [3.025195188893638e-127 * 0.0] + [1.300751704378113e-125 * 0.07] = 1.3749751018261018e-127

i: 2
α(100,2) = emi(2,100) * Σ[[α((100-1),1) * trans(1,2)] + [α((100-1),2) * trans(2,2)] + [α((100-1),3) * trans(3,2)]]
α(100,2) = 0.06927 * [1.2730468665857275e-126 * 0.0] + [3.025195188893638e-127 * 0.6] + [1.300751704378113e-125 * 0.03] = 3.96042374127613e-128

i: 3
α(100,3) = emi(3,100) * Σ[[α((100-1),1) * trans(1,3)] + [α((100-1),2) * trans(2,3)] + [α((100-1),3) * trans(3,3)]]
α(100,3) = 0.04995 * [1.2730468665857275e-126 * 0.03] + [3.025195188893638e-127 * 0.4] + [1.300751704378113e-125 * 0.9] = 5.9270492942016886e-127

Termination

$$P(O) = \sum_{i=1}^{N} \alpha(L,i)$$

P(O: MEILCEDNTSLSSIPNSLMQVDGDSGLYRNDFNSRDANSSDASNWTIDGENRTNLSFEGYLPPTCLSILHLQEKNWSALLTAVVIILTIAGNILVIMAVS) = α(100,1) + α(100,2) + α(100,3)
P(O: MEILCEDNTSLSSIPNSLMQVDGDSGLYRNDFNSRDANSSDASNWTIDGENRTNLSFEGYLPPTCLSILHLQEKNWSALLTAVVIILTIAGNILVIMAVS) = (1.3749751018261018e-127) + (3.96042374127613e-128) + (5.9270492942016886e-127) = 7.698066770155403e-127

Matrix of α values

	M_1	E_2	I_3	L_4	C_5	E_6	D_7	N_8	T_9	S_{10}	L_{11}	S_{12}	S_{13}	I_{14}	P_{15}	N_{16}	S_{17}
**I**	0.037890	0.001698	0.000071	0.000004	0.000000	0.000000	0.000000	0.000000	0.000000	0.000000	0.000000	0.000000	0.000000	0.000000	0.000000	0.000000	0.000
**O**	0.004080	0.000132	0.000003	0.000000	0.000000	0.000000	0.000000	0.000000	0.000000	0.000000	0.000000	0.000000	0.000000	0.000000	0.000000	0.000000	0.000
**M**	0.000000	0.000013	0.000014	0.000003	0.000000	0.000000	0.000000	0.000000	0.000000	0.000000	0.000000	0.000000	0.000000	0.000000	0.000000	0.000000	0.000

Viterbi

Initialization

$$\delta_0\left(S_0\right) = 1$$
$$\delta_0\left(S_i\right) = 0 \quad \forall i > 0$$

Where:

- S_1 = I
- S_2 = O
- S_3 = M

δ_0 (S_0) = 1
δ_0 (S_1) = 0
δ_0 (S_2) = 0
δ_0 (S_3) = 0

T: 1

i: 1
$\delta_1(S_1) = \Pi_1$ * emi(1,1)
$\delta_1(S_1)$ = 0.9 * 0.0421 = 0.03789
i: 2
$\delta_2(S_2) = \Pi_2$ * emi(1,2)
$\delta_2(S_2)$ = 0.1 * 0.0408 = 0.00408
i: 3
$\delta_3(S_3) = \Pi_3$ * emi(1,3)
$\delta_3(S_3)$ = 0.0 * 0.02568 = 0.0

Recursion

$$\delta_t\left(S_i\right) = \pi_i \cdot e_{q_t = s_i}\left(O_t\right) \quad 1 \leq i \leq N, \quad t = 1$$
$$\delta_1\left(S_i\right) = \pi_i \cdot e_{q_1 = s_i}\left(O_1\right) \quad 1 \leq i \leq N$$

$$\delta_t\left(S_j\right) = e_{q_t = s_j}\left(O_t\right) \cdot \max_i\left[\delta_{t-1}\left(S_i\right) \cdot a_{ij}\right] \quad 2 \leq t \leq L$$
$$1 \leq j \leq N$$

$$ptr_t\left(S_j\right) = \arg\max_i\left[\delta_{t-1}\left(S_i\right) \cdot a_{ij}\right]$$

T: 2

i: 1
$\delta_2(S_1)$ = emi(2,1) * max([$\delta_{2\text{-}1}$ * (S_1) * trans(1,1)])
$\delta_2(S_1)$ = 0.04621 * 0.036753299999999996 = 0.0016983699929999999
i: 2
$\delta_2(S_2)$ = emi(2,2) * max([$\delta_{2\text{-}1}$ * (S_2) * trans(2,1)])
$\delta_2(S_2)$ = 0.05409 * 0.002448 = 0.0001324123200000001
i: 3
$\delta_2(S_3)$ = emi(2,3) * max([$\delta_{2\text{-}1}$ * (S_3) * trans(3,1)])
$\delta_2(S_3)$ = 0.00457 * 0.0016320000000000002 = 7.458240000000001e-06

T: 3

i: 1
$\delta_3(S_1)$ = emi(3,1) * max([$\delta_{3\text{-}1}$ * (S_1) * trans(1,2)])
$\delta_3(S_1)$ = 0.04294 * 0.0016474188932099998 = 7.074016727443739e-05
i: 2
$\delta_3(S_2)$ = emi(3,2) * max([$\delta_{3\text{-}1}$ * (S_2) * trans(2,2)])
$\delta_3(S_2)$ = 0.04257 * 7.9447392e-05 = 3.38207547744e-06
i: 3

$\delta_3(S_3) = emi(3,3) * max([\delta_{3-1} * (S_3) * trans(3,2)])$
$\delta_3(S_3) = 0.11935 * 5.296492800000001e-05 = 6.321364156800001e-06$

T: 4

i: 1
$\delta_4(S_1) = emi(4,1) * max([\delta_{4-1} * (S_1) * trans(1,3)])$
$\delta_4(S_1) = 0.05171 * 6.861796225620427e-05 = 3.5482348282683226e-06$
i: 2
$\delta_4(S_2) = emi(4,2) * max([\delta_{4-1} * (S_2) * trans(2,3)])$
$\delta_4(S_2) = 0.1033 * 2.0292452864639997e-06 = 2.0962103809173118e-07$
i: 3
$\delta_4(S_3) = emi(4,3) * max([\delta_{4-1} * (S_3) * trans(3,3)])$
$\delta_4(S_3) = 0.19429 * 5.689227741120001e-06 = 1.105360057822205e-06$

T: 5

i: 1
$\delta_5(S_1) = emi(5,1) * max([\delta_{5-1} * (S_1) * trans(1,4)])$
$\delta_5(S_1) = 0.02019 * 3.441787783420273e-06 = 6.94896953472553e-08$
i: 2
$\delta_5(S_2) = emi(5,2) * max([\delta_{5-1} * (S_2) * trans(2,4)])$
$\delta_5(S_2) = 0.01597 * 1.257726228550387e-07 = 2.0085887869949684e-09$
i: 3
$\delta_5(S_3) = emi(5,3) * max([\delta_{5-1} * (S_3) * trans(3,4)])$
$\delta_5(S_3) = 0.01328 * 9.948240520399846e-07 = 1.3211263411090996e-08$

T: 6

i: 1
$\delta_6(S_1) = emi(6,1) * max([\delta_{6-1} * (S_1) * trans(1,5)])$
$\delta_6(S_1) = 0.04621 * 6.740500448683764e-08 = 3.1147852573367673e-09$
i: 2
$\delta_6(S_2) = emi(6,2) * max([\delta_{6-1} * (S_2) * trans(2,5)])$
$\delta_6(S_2) = 0.05409 * 1.205153272196981e-09 = 6.51867404931347e-11$
i: 3
$\delta_6(S_3) = emi(6,3) * max([\delta_{6-1} * (S_3) * trans(3,5)])$
$\delta_6(S_3) = 0.00457 * 1.1890137069981897e-08 = 5.433792640981727e-11$

T: 7

i: 1
$\delta_7(S_1) = emi(7,1) * max([\delta_{7-1} * (S_1) * trans(1,6)])$
$\delta_7(S_1) = 0.04143 * 3.021341699616664e-09 = 1.251741866151184e-10$
i: 2
$\delta_7(S_2) = emi(7,2) * max([\delta_{7-1} * (S_2) * trans(2,6)])$
$\delta_7(S_2) = 0.0399 * 3.911204429588082e-11 = 1.5605705674056445e-12$
i: 3
$\delta_7(S_3) = emi(7,3) * max([\delta_{7-1} * (S_3) * trans(3,6)])$
$\delta_7(S_3) = 0.00066 * 9.344355772010302e-11 = 6.1672748095268e-14$

T: 8

i: 1
$\delta_8(S_1) = emi(8,1) * max([\delta_{8-1} * (S_1) * trans(1,7)])$
$\delta_8(S_1) = 0.05877 * 1.2141896101666484e-10 = 7.135792338949393e-12$
i: 2
$\delta_8(S_2) = emi(8,2) * max([\delta_{8-1} * (S_2) * trans(2,7)])$
$\delta_8(S_2) = 0.04271 * 9.363423404433867e-13 = 3.999118136033704e-14$
i: 3
$\delta_8(S_3) = emi(8,3) * max([\delta_{8-1} * (S_3) * trans(3,7)])$
$\delta_8(S_3) = 0.01879 * 3.755225598453552e-12 = 7.056068899494225e-14$

T: 9

i: 1
$\delta_9(S_1) = emi(9,1) * max([\delta_{9-1} * (S_1) * trans(1,8)])$
$\delta_9(S_1) = 0.06521 * 6.921718568780911e-12 = 4.513652678702032e-13$
i: 2
$\delta_9(S_2) = emi(9,2) * max([\delta_{9-1} * (S_2) * trans(2,8)])$
$\delta_9(S_2) = 0.05586 * 2.3994708816202226e-14 = 1.3403443444730563e-15$
i: 3
$\delta_9(S_3) = emi(9,3) * max([\delta_{9-1} * (S_3) * trans(3,8)])$
$\delta_9(S_3) = 0.02246 * 2.140737701684818e-13 = 4.8080968779841014e-15$

T: 10

i: 1
$\delta_{10}(S_1) = emi(10,1) * max([\delta_{10-1} * (S_1) * trans(1,9)])$
$\delta_{10}(S_1) = 0.06409 * 4.378243098340971e-13 = 2.806016001726728e-14$
i: 2
$\delta_{10}(S_2) = emi(10,2) * max([\delta_{10-1} * (S_2) * trans(2,9)])$
$\delta_{10}(S_2) = 0.06927 * 8.042066606838338e-16 = 5.570739538556916e-17$

i: 3
$\delta_{10}(S_3)$ = emi(10,3) * max([$\delta_{10\text{-}1}$ * (S_3) * trans(3,9)])
$\delta_{10}(S_3)$ = 0.04995 * 1.3540958036106097e-14 = 6.763708539034995e-16

T: 11

i: 1
$\delta_{11}(S_1)$ = emi(11,1) * max([$\delta_{11\text{-}1}$ * (S_1) * trans(1,10)])
$\delta_{11}(S_1)$ = 0.05171 * 2.721835521674926e-14 = 1.4074611482581042e-15
i: 2
$\delta_{11}(S_2)$ = emi(11,2) * max([$\delta_{11\text{-}1}$ * (S_2) * trans(2,10)])
$\delta_{11}(S_2)$ = 0.1033 * 3.34244372313415e-17 = 3.452744365997577e-18
i: 3
$\delta_{11}(S_3)$ = emi(11,3) * max([$\delta_{11\text{-}1}$ * (S_3) * trans(3,10)])
$\delta_{11}(S_3)$ = 0.19429 * 8.418048005180183e-16 = 1.6355425469264578e-16

T: 12

i: 1
$\delta_{12}(S_1)$ = emi(12,1) * max([$\delta_{12\text{-}1}$ * (S_1) * trans(1,11)])
$\delta_{12}(S_1)$ = 0.06409 * 1.365237313810361e-15 = 8.749805944210603e-17
i: 2
$\delta_{12}(S_2)$ = emi(12,2) * max([$\delta_{12\text{-}1}$ * (S_2) * trans(2,11)])
$\delta_{12}(S_2)$ = 0.06927 * 4.906627640779373e-18 = 3.3988209667678715e-19
i: 3
$\delta_{12}(S_3)$ = emi(12,3) * max([$\delta_{12\text{-}1}$ * (S_3) * trans(3,11)])
$\delta_{12}(S_3)$ = 0.04995 * 1.471988292233812e-16 = 7.352581519707891e-18

T: 13

i: 1
$\delta_{13}(S_1)$ = emi(13,1) * max([$\delta_{13\text{-}1}$ * (S_1) * trans(1,12)])
$\delta_{13}(S_1)$ = 0.06409 * 8.487311765884284e-17 = 5.4395181107552375e-18
i: 2
$\delta_{13}(S_2)$ = emi(13,2) * max([$\delta_{13\text{-}1}$ * (S_2) * trans(2,12)])
$\delta_{13}(S_2)$ = 0.06927 * 2.205774455912367e-19 = 1.5279399656104965e-20
i: 3
$\delta_{13}(S_3)$ = emi(13,3) * max([$\delta_{13\text{-}1}$ * (S_3) * trans(3,12)])
$\delta_{13}(S_3)$ = 0.04995 * 6.617323367737102e-18 = 3.3053530221846824e-19

T: 14

i: 1
$\delta_{14}(S_1)$ = emi(14,1) * max([$\delta_{14\text{-}1}$ * (S_1) * trans(1,13)])
$\delta_{14}(S_1)$ = 0.04294 * 5.27633256743258e-18 = 2.26565720445555e-19
i: 2
$\delta_{14}(S_2)$ = emi(14,2) * max([$\delta_{14\text{-}1}$ * (S_2) * trans(2,13)])
$\delta_{14}(S_2)$ = 0.04257 * 9.916059066554047e-21 = 4.221266344632058e-22
i: 3
$\delta_{14}(S_3)$ = emi(14,3) * max([$\delta_{14\text{-}1}$ * (S_3) * trans(3,13)])
$\delta_{14}(S_3)$ = 0.11935 * 2.974817719966214e-19 = 3.550444948779677e-20

T: 15

i: 1
$\delta_{15}(S_1)$ = emi(15,1) * max([$\delta_{15\text{-}1}$ * (S_1) * trans(1,14)])
$\delta_{15}(S_1)$ = 0.03953 * 2.1976874883218837e-19 = 8.687458641336407e-21
i: 2
$\delta_{15}(S_2)$ = emi(15,2) * max([$\delta_{15\text{-}1}$ * (S_2) * trans(2,14)])
$\delta_{15}(S_2)$ = 0.05868 * 1.065133484633903e-21 = 6.250203287831742e-23
i: 3
$\delta_{15}(S_3)$ = emi(15,3) * max([$\delta_{15\text{-}1}$ * (S_3) * trans(3,14)])
$\delta_{15}(S_3)$ = 0.01557 * 3.195400453901709e-20 = 4.975238506724961e-22

T: 16

i: 1
$\delta_{16}(S_1)$ = emi(16,1) * max([$\delta_{16\text{-}1}$ * (S_1) * trans(1,15)])
$\delta_{16}(S_1)$ = 0.05877 * 8.426834882096315e-21 = 4.952450860208004e-22
i: 2
$\delta_{16}(S_2)$ = emi(16,2) * max([$\delta_{16\text{-}1}$ * (S_2) * trans(2,15)])
$\delta_{16}(S_2)$ = 0.04271 * 3.750121972699045e-23 = 1.601677094539762e-24
i: 3
$\delta_{16}(S_3)$ = emi(16,3) * max([$\delta_{16\text{-}1}$ * (S_3) * trans(3,15)])
$\delta_{16}(S_3)$ = 0.01879 * 4.477714656052465e-22 = 8.413625838722583e-24

T: 17

i: 1
$\delta_{17}(S_1)$ = emi(17,1) * max([$\delta_{17\text{-}1}$ * (S_1) * trans(1,16)])
$\delta_{17}(S_1)$ = 0.06409 * 4.803877334401763e-22 = 3.07880498361809e-23
i: 2
$\delta_{17}(S_2)$ = emi(17,2) * max([$\delta_{17\text{-}1}$ * (S_2) * trans(2,16)])

$\delta_{17}(S_2)$ = 0.06927 * 9.61006256723857e-25 = 6.656890340326157e-26
i: 3
$\delta_{17}(S_3)$ = emi(17,3) * max([δ_{17-1} * (S_3) * trans(3,16)])
$\delta_{17}(S_3)$ = 0.04995 * 1.4857352580624012e-23 = 7.421247614021695e-25

T: 18

i: 1
$\delta_{18}(S_1)$ = emi(18,1) * max([δ_{18-1} * (S_1) * trans(1,17)])
$\delta_{18}(S_1)$ = 0.05171 * 2.986440834109547e-23 = 1.5442885553180468e-24
i: 2
$\delta_{18}(S_2)$ = emi(18,2) * max([δ_{18-1} * (S_2) * trans(2,17)])
$\delta_{18}(S_2)$ = 0.1033 * 3.9941342041956944e-26 = 4.1259406329341526e-27
i: 3
$\delta_{18}(S_3)$ = emi(18,3) * max([δ_{18-1} * (S_3) * trans(3,17)])
$\delta_{18}(S_3)$ = 0.19429 * 9.23641495085427e-25 = 1.7945430608014759e-25

T: 19

i: 1
$\delta_{19}(S_1)$ = emi(19,1) * max([δ_{19-1} * (S_1) * trans(1,18)])
$\delta_{19}(S_1)$ = 0.0421 * 1.4979598986585053e-24 = 6.306411173352307e-26
i: 2
$\delta_{19}(S_2)$ = emi(19,2) * max([δ_{19-1} * (S_2) * trans(2,18)])
$\delta_{19}(S_2)$ = 0.0408 * 5.3836291824044275e-27 = 2.1965207064210064e-28
i: 3
$\delta_{19}(S_3)$ = emi(19,3) * max([δ_{19-1} * (S_3) * trans(3,18)])
$\delta_{19}(S_3)$ = 0.02568 * 1.6150887547213283e-25 = 4.147547922124371e-27

T: 20

i: 1
$\delta_{20}(S_1)$ = emi(20,1) * max([δ_{20-1} * (S_1) * trans(1,19)])
$\delta_{20}(S_1)$ = 0.05092 * 6.117218838151738e-26 = 3.114887832386865e-27
i: 2
$\delta_{20}(S_2)$ = emi(20,2) * max([δ_{20-1} * (S_2) * trans(2,19)])
$\delta_{20}(S_2)$ = 0.03962 * 1.3179124238526038e-28 = 5.2215690233040165e-30
i: 3
$\delta_{20}(S_3)$ = emi(20,3) * max([δ_{20-1} * (S_3) * trans(3,19)])
$\delta_{20}(S_3)$ = 0.00687 * 3.732793129911934e-27 = 2.564428880249499e-29

T: 21

i: 1
$\delta_{21}(S_1)$ = emi(21,1) * max([δ_{21-1} * (S_1) * trans(1,20)])
$\delta_{21}(S_1)$ = 0.04595 * 3.021441197415259e-27 = 1.3883522302123114e-28
i: 2
$\delta_{21}(S_2)$ = emi(21,2) * max([δ_{21-1} * (S_2) * trans(2,20)])
$\delta_{21}(S_2)$ = 0.04801 * 3.13294141398241e-30 = 1.5041251728529548e-31
i: 3
$\delta_{21}(S_3)$ = emi(21,3) * max([δ_{21-1} * (S_3) * trans(3,20)])
$\delta_{21}(S_3)$ = 0.14222 * 9.344663497160594e-29 = 1.3289980425661798e-29

T: 22

i: 1
$\delta_{22}(S_1)$ = emi(22,1) * max([δ_{22-1} * (S_1) * trans(1,21)])
$\delta_{22}(S_1)$ = 0.04143 * 1.346701663305942e-28 = 5.579384991076518e-30
i: 2
$\delta_{22}(S_2)$ = emi(22,2) * max([δ_{22-1} * (S_2) * trans(2,21)])
$\delta_{22}(S_2)$ = 0.0399 * 3.986994127698539e-31 = 1.5908106569517172e-32
i: 3
$\delta_{22}(S_3)$ = emi(22,3) * max([δ_{22-1} * (S_3) * trans(3,21)])
$\delta_{22}(S_3)$ = 0.00066 * 1.1960982383095619e-29 = 7.894248372843109e-33

T: 23

i: 1
$\delta_{23}(S_1)$ = emi(23,1) * max([δ_{23-1} * (S_1) * trans(1,22)])
$\delta_{23}(S_1)$ = 0.05591 * 5.412003441344222e-30 = 3.0258511240555547e-31
i: 2
$\delta_{23}(S_2)$ = emi(23,2) * max([δ_{23-1} * (S_2) * trans(2,22)])
$\delta_{23}(S_2)$ = 0.07239 * 9.544863941710303e-33 = 6.909527007404088e-34
i: 3
$\delta_{23}(S_3)$ = emi(23,3) * max([δ_{23-1} * (S_3) * trans(3,22)])
$\delta_{23}(S_3)$ = 0.06898 * 1.6738154973229554e-31 = 1.1545979300533747e-32

T: 24

i: 1
$\delta_{24}(S_1)$ = emi(24,1) * max([δ_{24-1} * (S_1) * trans(1,23)])
$\delta_{24}(S_1)$ = 0.04143 * 2.935075590333888e-31 = 1.21600181707533e-32
i: 2

$\delta_{24}(S_2)$ = emi(24,2) * max([δ_{24-1} * (S_2) * trans(2,23)])
$\delta_{24}(S_2)$ = 0.0399 * 4.145716204442453e-34 = 1.6541407655725387e-35
i: 3
$\delta_{24}(S_3)$ = emi(24,3) * max([δ_{24-1} * (S_3) * trans(3,23)])
$\delta_{24}(S_3)$ = 0.00066 * 1.0391381370480372e-32 = 6.858311704517045e-36

T: 25

i: 1
$\delta_{25}(S_1)$ = emi(25,1) * max([δ_{25-1} * (S_1) * trans(1,24)])
$\delta_{25}(S_1)$ = 0.06409 * 1.17952176256307e-32 = 7.559554976266716e-34
i: 2
$\delta_{25}(S_2)$ = emi(25,2) * max([δ_{25-1} * (S_2) * trans(2,24)])
$\delta_{25}(S_2)$ = 0.06927 * 9.924844593435232e-36 = 6.874939849872585e-37
i: 3
$\delta_{25}(S_3)$ = emi(25,3) * max([δ_{25-1} * (S_3) * trans(3,24)])
$\delta_{25}(S_3)$ = 0.04995 * 3.6480054512259896e-34 = 1.8221787228873818e-35

T: 26

i: 1
$\delta_{26}(S_1)$ = emi(26,1) * max([δ_{26-1} * (S_1) * trans(1,25)])
$\delta_{26}(S_1)$ = 0.05591 * 7.332768326978714e-34 = 4.099750771613799e-35
i: 2
$\delta_{26}(S_2)$ = emi(26,2) * max([δ_{26-1} * (S_2) * trans(2,25)])
$\delta_{26}(S_2)$ = 0.07239 * 5.4665361686621454e-37 = 3.9572255324945267e-38
i: 3
$\delta_{26}(S_3)$ = emi(26,3) * max([δ_{26-1} * (S_3) * trans(3,25)])
$\delta_{26}(S_3)$ = 0.06898 * 2.2678664928800147e-35 = 1.564374306788634e-36

T: 27

i: 1
$\delta_{27}(S_1)$ = emi(27,1) * max([δ_{27-1} * (S_1) * trans(1,26)])
$\delta_{27}(S_1)$ = 0.05171 * 3.976758248465385e-35 = 2.0563816902814504e-36
i: 2
$\delta_{27}(S_2)$ = emi(27,2) * max([δ_{27-1} * (S_2) * trans(2,26)])
$\delta_{27}(S_2)$ = 0.1033 * 4.6931229203659017e-38 = 4.847995976737977e-39
i: 3
$\delta_{27}(S_3)$ = emi(27,3) * max([δ_{27-1} * (S_3) * trans(3,26)])
$\delta_{27}(S_3)$ = 0.19429 * 1.4079368761097706e-36 = 2.735480556593673e-37

T: 28

i: 1
$\delta_{28}(S_1)$ = emi(28,1) * max([δ_{28-1} * (S_1) * trans(1,27)])
$\delta_{28}(S_1)$ = 0.03296 * 1.9946902395730068e-36 = 6.574499029632631e-38
i: 2
$\delta_{28}(S_2)$ = emi(28,2) * max([δ_{28-1} * (S_2) * trans(2,27)])
$\delta_{28}(S_2)$ = 0.0261 * 8.206441669781018e-39 = 2.141881275812846e-40
i: 3
$\delta_{28}(S_3)$ = emi(28,3) * max([δ_{28-1} * (S_3) * trans(3,27)])
$\delta_{28}(S_3)$ = 0.06541 * 2.4619325009343056e-37 = 1.6103500488611292e-38

T: 29

i: 1
$\delta_{29}(S_1)$ = emi(29,1) * max([δ_{29-1} * (S_1) * trans(1,28)])
$\delta_{29}(S_1)$ = 0.11518 * 6.377264058743651e-38 = 7.345332742860938e-39
i: 2
$\delta_{29}(S_2)$ = emi(29,2) * max([δ_{29-1} * (S_2) * trans(2,28)])
$\delta_{29}(S_2)$ = 0.07232 * 4.8310501465833874e-40 = 3.4938154660091057e-41
i: 3
$\delta_{29}(S_3)$ = emi(29,3) * max([δ_{29-1} * (S_3) * trans(3,28)])
$\delta_{29}(S_3)$ = 0.00852 * 1.4493150439750162e-38 = 1.2348164174667137e-40

T: 30

i: 1
$\delta_{30}(S_1)$ = emi(30,1) * max([δ_{30-1} * (S_1) * trans(1,29)])
$\delta_{30}(S_1)$ = 0.05877 * 7.12497276057511e-39 = 4.187346491389992e-40
i: 2
$\delta_{30}(S_2)$ = emi(30,2) * max([δ_{30-1} * (S_2) * trans(2,29)])
$\delta_{30}(S_2)$ = 0.04271 * 2.0962892796054633e-41 = 8.953251513194933e-43
i: 3
$\delta_{30}(S_3)$ = emi(30,3) * max([δ_{30-1} * (S_3) * trans(3,29)])
$\delta_{30}(S_3)$ = 0.01879 * 2.2035998228582813e-40 = 4.140564067150711e-42

T: 31

i: 1
$\delta_{31}(S_1)$ = emi(31,1) * max([δ_{31-1} * (S_1) * trans(1,30)])
$\delta_{31}(S_1)$ = 0.04143 * 4.061726096648292e-40 = 1.6827731218413874e-41

i: 2
$\delta_{31}(S_2)$ = emi(31,2) * max([δ_{31-1} * (S_2) * trans(2,30)])
$\delta_{31}(S_2)$ = 0.0399 * 5.37195090791696e-43 = 2.143408412258867e-44
i: 3
$\delta_{31}(S_3)$ = emi(31,3) * max([δ_{31-1} * (S_3) * trans(3,30)])
$\delta_{31}(S_3)$ = 0.00066 * 1.2562039474169975e-41 = 8.290946052952183e-45

T: 32

i: 1
$\delta_{32}(S_1)$ = emi(32,1) * max([δ_{32-1} * (S_1) * trans(1,31)])
$\delta_{32}(S_1)$ = 0.03004 * 1.6322899281861457e-41 = 4.903398944271182e-43
i: 2
$\delta_{32}(S_2)$ = emi(32,2) * max([δ_{32-1} * (S_2) * trans(2,31)])
$\delta_{32}(S_2)$ = 0.05577 * 1.2860450473553202e-44 = 7.172273229100621e-46
i: 3
$\delta_{32}(S_3)$ = emi(32,3) * max([δ_{32-1} * (S_3) * trans(3,31)])
$\delta_{32}(S_3)$ = 0.09148 * 5.0483193655524162e-43 = 4.618202555581504e-44

T: 33

i: 1
$\delta_{33}(S_1)$ = emi(33,1) * max([δ_{33-1} * (S_1) * trans(1,32)])
$\delta_{33}(S_1)$ = 0.05877 * 4.756296975943047e-43 = 2.7952757327617285e-44
i: 2
$\delta_{33}(S_2)$ = emi(33,2) * max([δ_{33-1} * (S_2) * trans(2,32)])
$\delta_{33}(S_2)$ = 0.04271 * 1.3854607666744511e-45 = 5.91730293446658e-47
i: 3
$\delta_{33}(S_3)$ = emi(33,3) * max([δ_{33-1} * (S_3) * trans(3,32)])
$\delta_{33}(S_3)$ = 0.01879 * 4.156382300023354e-44 = 7.809842341743883e-46

T: 34

i: 1
$\delta_{34}(S_1)$ = emi(34,1) * max([δ_{34-1} * (S_1) * trans(1,33)])
$\delta_{34}(S_1)$ = 0.06409 * 2.7114174607788767e-44 = 1.737747450613182e-45
i: 2
$\delta_{34}(S_2)$ = emi(34,2) * max([δ_{34-1} * (S_2) * trans(2,33)])
$\delta_{34}(S_2)$ = 0.06927 * 3.5503817606799483e-47 = 2.4593494456230002e-48
i: 3
$\delta_{34}(S_3)$ = emi(34,3) * max([δ_{34-1} * (S_3) * trans(3,33)])
$\delta_{34}(S_3)$ = 0.04995 * 8.385827198285185e-46 = 4.1887206855434496e-47

T: 35

i: 1
$\delta_{35}(S_1)$ = emi(35,1) * max([δ_{35-1} * (S_1) * trans(1,34)])
$\delta_{35}(S_1)$ = 0.11518 * 1.6856150270947864e-45 = 1.9414913882077752e-46
i: 2
$\delta_{35}(S_2)$ = emi(35,2) * max([δ_{35-1} * (S_2) * trans(2,34)])
$\delta_{35}(S_2)$ = 0.07232 * 1.4756096673738e-48 = 1.0671609114447322e-49
i: 3
$\delta_{35}(S_3)$ = emi(35,3) * max([δ_{35-1} * (S_3) * trans(3,34)])
$\delta_{35}(S_3)$ = 0.00852 * 5.2132423518395455e-47 = 4.4416824837672926e-49

T: 36

i: 1
$\delta_{36}(S_1)$ = emi(36,1) * max([δ_{36-1} * (S_1) * trans(1,35)])
$\delta_{36}(S_1)$ = 0.04143 * 1.883246646561542e-46 = 7.802290856704469e-48
i: 2
$\delta_{36}(S_2)$ = emi(36,2) * max([δ_{36-1} * (S_2) * trans(2,35)])
$\delta_{36}(S_2)$ = 0.0399 * 6.402965468668392e-50 = 2.5547832219986884e-51
i: 3
$\delta_{36}(S_3)$ = emi(36,3) * max([δ_{36-1} * (S_3) * trans(3,35)])
$\delta_{36}(S_3)$ = 0.00066 * 5.824474164623325e-48 = 3.8441529486513947e-51

T: 37

i: 1
$\delta_{37}(S_1)$ = emi(37,1) * max([δ_{37-1} * (S_1) * trans(1,36)])
$\delta_{37}(S_1)$ = 0.06901 * 7.568222131003334e-48 = 5.222830092605401e-49
i: 2
$\delta_{37}(S_2)$ = emi(37,2) * max([δ_{37-1} * (S_2) * trans(2,36)])
$\delta_{37}(S_2)$ = 0.07052 * 1.532869933199213e-51 = 1.080979876892085e-52
i: 3
$\delta_{37}(S_3)$ = emi(37,3) * max([δ_{37-1} * (S_3) * trans(3,36)])
$\delta_{37}(S_3)$ = 0.09536 * 2.3406872570113404e-49 = 2.232079368286014e-50

T: 38

i: 1
$\delta_{38}(S_1)$ = emi(38,1) * max([δ_{38-1} * (S_1) * trans(1,37)])

$\delta_{38}(S_1) = 0.05877 * 5.0661451898272385e-49 = 2.9773735280614684e-50$
i: 2
$\delta_{38}(S_2)$ = emi(38,2) * max([δ_{38-1} * (S_2) * trans(2,37)])
$\delta_{38}(S_2) = 0.04271 * 6.696238104858042e-52 = 2.8599632945848694e-53$
i: 3
$\delta_{38}(S_3)$ = emi(38,3) * max([δ_{38-1} * (S_3) * trans(3,37)])
$\delta_{38}(S_3) = 0.01879 * 2.0088714314574129e-50 = 3.774669419708479e-52$

T: 39

i: 1
$\delta_{39}(S_1)$ = emi(39,1) * max([δ_{39-1} * (S_1) * trans(1,38)])
$\delta_{39}(S_1) = 0.06409 * 2.8880523222196245e-50 = 1.850952733310557e-51$
i: 2
$\delta_{39}(S_2)$ = emi(39,2) * max([δ_{39-1} * (S_2) * trans(2,38)])
$\delta_{39}(S_2) = 0.06927 * 1.7159779767509216e-53 = 1.1886579444953634e-54$
i: 3
$\delta_{39}(S_3)$ = emi(39,3) * max([δ_{39-1} * (S_3) * trans(3,38)])
$\delta_{39}(S_3) = 0.04995 * 8.932120584184404e-52 = 4.46159423180011e-53$

T: 40

i: 1
$\delta_{40}(S_1)$ = emi(40,1) * max([δ_{40-1} * (S_1) * trans(1,39)])
$\delta_{40}(S_1) = 0.06409 * 1.7954241513112403e-51 = 1.1506873385753738e-52$
i: 2
$\delta_{40}(S_2)$ = emi(40,2) * max([δ_{40-1} * (S_2) * trans(2,39)])
$\delta_{40}(S_2) = 0.06927 * 1.338478269540033e-54 = 9.271638973103809e-56$
i: 3
$\delta_{40}(S_3)$ = emi(40,3) * max([δ_{40-1} * (S_3) * trans(3,39)])
$\delta_{40}(S_3) = 0.04995 * 5.552858199931671e-53 = 2.7736526708658694e-54$

T: 41

i: 1
$\delta_{41}(S_1)$ = emi(41,1) * max([δ_{41-1} * (S_1) * trans(1,40)])
$\delta_{41}(S_1) = 0.04143 * 1.1161667184181125e-52 = 4.6242787144062406e-54$
i: 2
$\delta_{41}(S_2)$ = emi(41,2) * max([δ_{41-1} * (S_2) * trans(2,40)])
$\delta_{41}(S_2) = 0.0399 * 8.320958012597608e-56 = 3.320062247026445e-57$
i: 3
$\delta_{41}(S_3)$ = emi(41,3) * max([δ_{41-1} * (S_3) * trans(3,40)])
$\delta_{41}(S_3) = 0.00066 * 3.4520620157261214e-54 = 2.27836093037924e-57$

T: 42

i: 1
$\delta_{42}(S_1)$ = emi(42,1) * max([δ_{42-1} * (S_1) * trans(1,41)])
$\delta_{42}(S_1) = 0.06901 * 4.485550352974053e-54 = 3.095478298587394e-55$
i: 2
$\delta_{42}(S_2)$ = emi(42,2) * max([δ_{42-1} * (S_2) * trans(2,41)])
$\delta_{42}(S_2) = 0.07052 * 1.992037348215867e-57 = 1.4047847379618294e-58$
i: 3
$\delta_{42}(S_3)$ = emi(42,3) * max([δ_{42-1} * (S_3) * trans(3,41)])
$\delta_{42}(S_3) = 0.09536 * 1.387283614321872e-55 = 1.3229136546173372e-56$

T: 43

i: 1
$\delta_{43}(S_1)$ = emi(43,1) * max([δ_{43-1} * (S_1) * trans(1,42)])
$\delta_{43}(S_1) = 0.06409 * 3.0026139496297724e-55 = 1.924375280317721e-56$
i: 2
$\delta_{43}(S_2)$ = emi(43,2) * max([δ_{43-1} * (S_2) * trans(2,42)])
$\delta_{43}(S_2) = 0.06927 * 3.9687409638520115e-58 = 2.7491468656602884e-59$
i: 3
$\delta_{43}(S_3)$ = emi(43,3) * max([δ_{43-1} * (S_3) * trans(3,42)])
$\delta_{43}(S_3) = 0.04995 * 1.1906222891556035e-56 = 5.9471583343322394e-58$

T: 44

i: 1
$\delta_{44}(S_1)$ = emi(44,1) * max([δ_{44-1} * (S_1) * trans(1,43)])
$\delta_{44}(S_1) = 0.05877 * 1.8666440219081893e-56 = 1.097026691675443e-57$
i: 2
$\delta_{44}(S_2)$ = emi(44,2) * max([δ_{44-1} * (S_2) * trans(2,43)])
$\delta_{44}(S_2) = 0.04271 * 1.7841475002996719e-59 = 7.620093973779898e-61$
i: 3
$\delta_{44}(S_3)$ = emi(44,3) * max([δ_{44-1} * (S_3) * trans(3,43)])
$\delta_{44}(S_3) = 0.01879 * 5.773125840953163e-58 = 1.0847703455150994e-59$

T: 45

i: 1

$\delta_{45}(S_1)$ = emi(45,1) * max([δ_{45-1} * (S_1) * trans(1,44)])
$\delta_{45}(S_1)$ = 0.01482 * 1.0641158909251796e-57 = 1.5770197503511162e-59
i: 2
$\delta_{45}(S_2)$ = emi(45,2) * max([δ_{45-1} * (S_2) * trans(2,44)])
$\delta_{45}(S_2)$ = 0.01439 * 4.572056384267938e-61 = 6.579189136961563e-63
i: 3
$\delta_{45}(S_3)$ = emi(45,3) * max([δ_{45-1} * (S_3) * trans(3,44)])
$\delta_{45}(S_3)$ = 0.04444 * 3.2910800750263287e-59 = 1.4625559853417004e-60

T: 46

i: 1
$\delta_{46}(S_1)$ = emi(46,1) * max([δ_{46-1} * (S_1) * trans(1,45)])
$\delta_{46}(S_1)$ = 0.06521 * 1.5297091578405826e-59 = 9.97523341827844e-61
i: 2
$\delta_{46}(S_2)$ = emi(46,2) * max([δ_{46-1} * (S_2) * trans(2,45)])
$\delta_{46}(S_2)$ = 0.05586 * 4.387667956025101e-62 = 2.450951320235621e-63
i: 3
$\delta_{46}(S_3)$ = emi(46,3) * max([δ_{46-1} * (S_3) * trans(3,45)])
$\delta_{46}(S_3)$ = 0.02246 * 1.3163003868075305e-60 = 2.9564106687697133e-62

T: 47

i: 1
$\delta_{47}(S_1)$ = emi(47,1) * max([δ_{47-1} * (S_1) * trans(1,46)])
$\delta_{47}(S_1)$ = 0.04294 * 9.675976415730087e-61 = 4.1548642729144995e-62
i: 2
$\delta_{47}(S_2)$ = emi(47,2) * max([δ_{47-1} * (S_2) * trans(2,46)])
$\delta_{47}(S_2)$ = 0.04257 * 1.4705707921413727e-63 = 6.260219862145823e-65
i: 3
$\delta_{47}(S_3)$ = emi(47,3) * max([δ_{47-1} * (S_3) * trans(3,46)])
$\delta_{47}(S_3)$ = 0.11935 * 2.992570025483532e-62 = 3.5716323254145955e-63

T: 48

i: 1
$\delta_{48}(S_1)$ = emi(48,1) * max([δ_{48-1} * (S_1) * trans(1,47)])
$\delta_{48}(S_1)$ = 0.04143 * 4.030218344727064e-62 = 1.6697194602204228e-63
i: 2
$\delta_{48}(S_2)$ = emi(48,2) * max([δ_{48-1} * (S_2) * trans(2,47)])
$\delta_{48}(S_2)$ = 0.0399 * 1.0714896976243786e-64 = 4.2752438935212703e-66
i: 3
$\delta_{48}(S_3)$ = emi(48,3) * max([δ_{48-1} * (S_3) * trans(3,47)])
$\delta_{48}(S_3)$ = 0.00066 * 3.214469092873136e-63 = 2.1215496012962696e-66

T: 49

i: 1
$\delta_{49}(S_1)$ = emi(49,1) * max([δ_{49-1} * (S_1) * trans(1,48)])
$\delta_{49}(S_1)$ = 0.05591 * 1.61962787641381e-63 = 9.055339457029613e-65
i: 2
$\delta_{49}(S_2)$ = emi(49,2) * max([δ_{49-1} * (S_2) * trans(2,48)])
$\delta_{49}(S_2)$ = 0.07239 * 2.565146336112762e-66 = 1.8569094327120283e-67
i: 3
$\delta_{49}(S_3)$ = emi(49,3) * max([δ_{49-1} * (S_3) * trans(3,48)])
$\delta_{49}(S_3)$ = 0.06898 * 5.0091583806612684e-65 = 3.455317450980143e-66

T: 50

i: 1
$\delta_{50}(S_1)$ = emi(50,1) * max([δ_{50-1} * (S_1) * trans(1,49)])
$\delta_{50}(S_1)$ = 0.04621 * 8.783679273318724e-65 = 4.0589381922005824e-66
i: 2
$\delta_{50}(S_2)$ = emi(50,2) * max([δ_{50-1} * (S_2) * trans(2,49)])
$\delta_{50}(S_2)$ = 0.05409 * 1.114145659627217e-67 = 6.026413872923617e-69
i: 3
$\delta_{50}(S_3)$ = emi(50,3) * max([δ_{50-1} * (S_3) * trans(3,49)])
$\delta_{50}(S_3)$ = 0.00457 * 3.1097857058821287e-66 = 1.421172067588133e-68

T: 51

i: 1
$\delta_{51}(S_1)$ = emi(51,1) * max([δ_{51-1} * (S_1) * trans(1,50)])
$\delta_{51}(S_1)$ = 0.05877 * 3.937170046434565e-66 = 2.313874836289594e-67
i: 2
$\delta_{51}(S_2)$ = emi(51,2) * max([δ_{51-1} * (S_2) * trans(2,50)])
$\delta_{51}(S_2)$ = 0.04271 * 3.61584832375417e-69 = 1.544328819075406e-70
i: 3
$\delta_{51}(S_3)$ = emi(51,3) * max([δ_{51-1} * (S_3) * trans(3,50)])
$\delta_{51}(S_3)$ = 0.01879 * 1.2176814576601746e-67 = 2.2880234589434683e-69

T: 52

i: 1
$\delta_{52}(S_1)$ = emi(52,1) * max([δ_{52-1} * (S_1) * trans(1,51)])
$\delta_{52}(S_1)$ = 0.11518 * 2.2444585912009064e-67 = 2.585167405345204e-68
i: 2
$\delta_{52}(S_2)$ = emi(52,2) * max([δ_{52-1} * (S_2) * trans(2,51)])
$\delta_{52}(S_2)$ = 0.07232 * 9.265972914452436e-71 = 6.701151611732002e-72
i: 3
$\delta_{52}(S_3)$ = emi(52,3) * max([δ_{52-1} * (S_3) * trans(3,51)])
$\delta_{52}(S_3)$ = 0.00852 * 6.941624508868782e-69 = 5.914264081556202e-71

T: 53

i: 1
$\delta_{53}(S_1)$ = emi(53,1) * max([δ_{53-1} * (S_1) * trans(1,52)])
$\delta_{53}(S_1)$ = 0.06521 * 2.507612383184848e-68 = 1.6352140350748393e-69
i: 2
$\delta_{53}(S_2)$ = emi(53,2) * max([δ_{53-1} * (S_2) * trans(2,52)])
$\delta_{53}(S_2)$ = 0.05586 * 4.020690967039201e-72 = 2.2459579741880977e-73
i: 3
$\delta_{53}(S_3)$ = emi(53,3) * max([δ_{53-1} * (S_3) * trans(3,52)])
$\delta_{53}(S_3)$ = 0.02246 * 7.755502216035611e-70 = 1.7418857977215982e-71

T: 54

i: 1
$\delta_{54}(S_1)$ = emi(54,1) * max([δ_{54-1} * (S_1) * trans(1,53)])
$\delta_{54}(S_1)$ = 0.05877 * 1.5861576140225941e-69 = 9.321848297610786e-71
i: 2
$\delta_{54}(S_2)$ = emi(54,2) * max([δ_{54-1} * (S_2) * trans(2,53)])
$\delta_{54}(S_2)$ = 0.04271 * 5.225657393164794e-73 = 2.2318782726206833e-74
i: 3
$\delta_{54}(S_3)$ = emi(54,3) * max([δ_{54-1} * (S_3) * trans(3,53)])
$\delta_{54}(S_3)$ = 0.01879 * 4.905642105224518e-71 = 9.21770151571687e-73

T: 55

i: 1
$\delta_{55}(S_1)$ = emi(55,1) * max([δ_{55-1} * (S_1) * trans(1,54)])
$\delta_{55}(S_1)$ = 0.05171 * 9.042192848682462e-71 = 4.675717922053701e-72
i: 2
$\delta_{55}(S_2)$ = emi(55,2) * max([δ_{55-1} * (S_2) * trans(2,54)])
$\delta_{55}(S_2)$ = 0.1033 * 2.7653104547150606e-74 = 2.8565656997206575e-75
i: 3
$\delta_{55}(S_3)$ = emi(55,3) * max([δ_{55-1} * (S_3) * trans(3,54)])
$\delta_{55}(S_3)$ = 0.19429 * 2.7965544892832357e-72 = 5.433425717228398e-73

T: 56

i: 1
$\delta_{56}(S_1)$ = emi(56,1) * max([δ_{56-1} * (S_1) * trans(1,55)])
$\delta_{56}(S_1)$ = 0.06409 * 4.53544638439209e-72 = 2.9067675877568905e-73
i: 2
$\delta_{56}(S_2)$ = emi(56,2) * max([δ_{56-1} * (S_2) * trans(2,55)])
$\delta_{56}(S_2)$ = 0.06927 * 1.6300277151685193e-74 = 1.1291201982972332e-75
i: 3
$\delta_{56}(S_3)$ = emi(56,3) * max([δ_{56-1} * (S_3) * trans(3,55)])
$\delta_{56}(S_3)$ = 0.04995 * 4.890083145505558e-73 = 2.442596531180026e-74

T: 57

i: 1
$\delta_{57}(S_1)$ = emi(57,1) * max([δ_{57-1} * (S_1) * trans(1,56)])
$\delta_{57}(S_1)$ = 0.03004 * 2.8195645601241837e-73 = 8.469971938613048e-75
i: 2
$\delta_{57}(S_2)$ = emi(57,2) * max([δ_{57-1} * (S_2) * trans(2,56)])
$\delta_{57}(S_2)$ = 0.05577 * 7.327789593540078e-76 = 4.086708256317302e-77
i: 3
$\delta_{57}(S_3)$ = emi(57,3) * max([δ_{57-1} * (S_3) * trans(3,56)])
$\delta_{57}(S_3)$ = 0.09148 * 2.1983368780620234e-74 = 2.0110385760511392e-75

T: 58

i: 1
$\delta_{58}(S_1)$ = emi(58,1) * max([δ_{58-1} * (S_1) * trans(1,57)])
$\delta_{58}(S_1)$ = 0.04621 * 8.215872780454656e-75 = 3.7965548118480967e-76
i: 2
$\delta_{58}(S_2)$ = emi(58,2) * max([δ_{58-1} * (S_2) * trans(2,57)])
$\delta_{58}(S_2)$ = 0.05409 * 6.0331157281534174e-77 = 3.2633122973581836e-78
i: 3
$\delta_{58}(S_3)$ = emi(58,3) * max([δ_{58-1} * (S_3) * trans(3,57)])
$\delta_{58}(S_3)$ = 0.00457 * 1.8099347184460254e-75 = 8.271401663298337e-78

T: 59

i: 1
$\delta_{59}(S_1)$ = emi(59,1) * max([δ_{59-1} * (S_1) * trans(1,58)])
$\delta_{59}(S_1)$ = 0.05591 * 3.682658167492654e-76 = 2.0589741814451427e-77
i: 2
$\delta_{59}(S_2)$ = emi(59,2) * max([δ_{59-1} * (S_2) * trans(2,58)])
$\delta_{59}(S_2)$ = 0.07239 * 1.95798737841491e-78 = 1.4173870632345533e-79
i: 3
$\delta_{59}(S_3)$ = emi(59,3) * max([δ_{59-1} * (S_3) * trans(3,58)])
$\delta_{59}(S_3)$ = 0.06898 * 1.1389664435544429e-77 = 7.856590527638452e-79

T: 60

i: 1
$\delta_{60}(S_1)$ = emi(60,1) * max([δ_{60-1} * (S_1) * trans(1,59)])
$\delta_{60}(S_1)$ = 0.03296 * 1.9972049560017884e-77 = 6.582787534981895e-79
i: 2
$\delta_{60}(S_2)$ = emi(60,2) * max([δ_{60-1} * (S_2) * trans(2,59)])
$\delta_{60}(S_2)$ = 0.0261 * 8.50432237940732e-80 = 2.2196281410253106e-81
i: 3
$\delta_{60}(S_3)$ = emi(60,3) * max([δ_{60-1} * (S_3) * trans(3,59)])
$\delta_{60}(S_3)$ = 0.06541 * 7.070931474874607e-79 = 4.6250962777154796e-80

T: 61

i: 1
$\delta_{61}(S_1)$ = emi(61,1) * max([δ_{61-1} * (S_1) * trans(1,60)])
$\delta_{61}(S_1)$ = 0.05171 * 6.385303908932438e-79 = 3.3018406513089638e-80
i: 2
$\delta_{61}(S_2)$ = emi(61,2) * max([δ_{61-1} * (S_2) * trans(2,60)])
$\delta_{61}(S_2)$ = 0.1033 * 1.387528883314644e-81 = 1.4333173364640272e-82
i: 3
$\delta_{61}(S_3)$ = emi(61,3) * max([δ_{61-1} * (S_3) * trans(3,60)])
$\delta_{61}(S_3)$ = 0.19429 * 4.162586649943932e-80 = 8.087489602176064e-81

T: 62

i: 1
$\delta_{62}(S_1)$ = emi(62,1) * max([δ_{62-1} * (S_1) * trans(1,61)])
$\delta_{62}(S_1)$ = 0.03953 * 3.2027854317696947e-80 = 1.2660610811785604e-81
i: 2
$\delta_{62}(S_2)$ = emi(62,2) * max([δ_{62-1} * (S_2) * trans(2,61)])
$\delta_{62}(S_2)$ = 0.05868 * 2.4262468806528192e-82 = 1.4237216695670744e-83
i: 3
$\delta_{62}(S_3)$ = emi(62,3) * max([δ_{62-1} * (S_3) * trans(3,61)])
$\delta_{62}(S_3)$ = 0.01557 * 7.2787406419584585e-81 = 1.133299917952932e-82

T: 63

i: 1
$\delta_{63}(S_1)$ = emi(63,1) * max([δ_{63-1} * (S_1) * trans(1,62)])
$\delta_{63}(S_1)$ = 0.03953 * 1.2280792487432036e-81 = 4.854597270281884e-83
i: 2
$\delta_{63}(S_2)$ = emi(63,2) * max([δ_{63-1} * (S_2) * trans(2,62)])
$\delta_{63}(S_2)$ = 0.05868 * 8.542330017402447e-84 = 5.012639254211756e-85
i: 3
$\delta_{63}(S_3)$ = emi(63,3) * max([δ_{63-1} * (S_3) * trans(3,62)])
$\delta_{63}(S_3)$ = 0.01557 * 1.0199699261576388e-82 = 1.5880931750274435e-84

T: 64

i: 1
$\delta_{64}(S_1)$ = emi(64,1) * max([δ_{64-1} * (S_1) * trans(1,63)])
$\delta_{64}(S_1)$ = 0.06521 * 4.708959352173427e-83 = 3.070712393552292e-84
i: 2
$\delta_{64}(S_2)$ = emi(64,2) * max([δ_{64-1} * (S_2) * trans(2,63)])
$\delta_{64}(S_2)$ = 0.05586 * 3.0075835525270536e-85 = 1.680036172441612e-86
i: 3
$\delta_{64}(S_3)$ = emi(64,3) * max([δ_{64-1} * (S_3) * trans(3,63)])
$\delta_{64}(S_3)$ = 0.02246 * 1.4563791810845652e-84 = 3.271027640715933e-86

T: 65

i: 1
$\delta_{65}(S_1)$ = emi(65,1) * max([δ_{65-1} * (S_1) * trans(1,64)])
$\delta_{65}(S_1)$ = 0.02019 * 2.978591021745723e-84 = 6.013775272904615e-86
i: 2
$\delta_{65}(S_2)$ = emi(65,2) * max([δ_{65-1} * (S_2) * trans(2,64)])
$\delta_{65}(S_2)$ = 0.01597 * 1.0080217034649672e-86 = 1.6098106604335527e-88
i: 3
$\delta_{65}(S_3)$ = emi(65,3) * max([δ_{65-1} * (S_3) * trans(3,64)])
$\delta_{65}(S_3)$ = 0.01328 * 9.212137180656876e-86 = 1.223371817591233e-87

T: 66

i: 1
$\delta_{66}(S_1)$ = emi(66,1) * max([δ_{66-1} * (S_1) * trans(1,65)])
$\delta_{66}(S_1)$ = 0.05171 * 5.833362014717476e-86 = 3.016431497810407e-87
i: 2
$\delta_{66}(S_2)$ = emi(66,2) * max([δ_{66-1} * (S_2) * trans(2,65)])
$\delta_{66}(S_2)$ = 0.1033 * 9.658863962601316e-89 = 9.97760647336716e-90
i: 3
$\delta_{66}(S_3)$ = emi(66,3) * max([δ_{66-1} * (S_3) * trans(3,65)])
$\delta_{66}(S_3)$ = 0.19429 * 1.8041325818713846e-87 = 3.505249193317913e-88

T: 67

i: 1
$\delta_{67}(S_1)$ = emi(67,1) * max([δ_{67-1} * (S_1) * trans(1,66)])
$\delta_{67}(S_1)$ = 0.06409 * 2.9259385528760946e-87 = 1.8752340185382889e-88
i: 2
$\delta_{67}(S_2)$ = emi(67,2) * max([δ_{67-1} * (S_2) * trans(2,66)])
$\delta_{67}(S_2)$ = 0.06927 * 1.0515747579953739e-89 = 7.284258348633955e-91
i: 3
$\delta_{67}(S_3)$ = emi(67,3) * max([δ_{67-1} * (S_3) * trans(3,66)])
$\delta_{67}(S_3)$ = 0.04995 * 3.1547242739861215e-88 = 1.5757847748560677e-89

T: 68

i: 1
$\delta_{68}(S_1)$ = emi(68,1) * max([δ_{68-1} * (S_1) * trans(1,67)])
$\delta_{68}(S_1)$ = 0.04294 * 1.8189769979821402e-88 = 7.81068722933531e-90
i: 2
$\delta_{68}(S_2)$ = emi(68,2) * max([δ_{68-1} * (S_2) * trans(2,67)])
$\delta_{68}(S_2)$ = 0.04257 * 4.727354324568203e-91 = 2.0124347359686837e-92
i: 3
$\delta_{68}(S_3)$ = emi(68,3) * max([δ_{68-1} * (S_3) * trans(3,67)])
$\delta_{68}(S_3)$ = 0.11935 * 1.418206297370461e-89 = 1.6926292159116451e-90

T: 69

i: 1
$\delta_{69}(S_1)$ = emi(69,1) * max([δ_{69-1} * (S_1) * trans(1,68)])
$\delta_{69}(S_1)$ = 0.05171 * 7.576366612455251e-90 = 3.91773917530061e-91
i: 2
$\delta_{69}(S_2)$ = emi(69,2) * max([δ_{69-1} * (S_2) * trans(2,68)])
$\delta_{69}(S_2)$ = 0.1033 * 5.077887647734935e-92 = 5.245457940110188e-93
i: 3
$\delta_{69}(S_3)$ = emi(69,3) * max([δ_{69-1} * (S_3) * trans(3,68)])
$\delta_{69}(S_3)$ = 0.19429 * 1.5233662943204807e-90 = 2.959748373235262e-91

T: 70

i: 1
$\delta_{70}(S_1)$ = emi(70,1) * max([δ_{70-1} * (S_1) * trans(1,69)])
$\delta_{70}(S_1)$ = 0.02253 * 3.800207000041592e-91 = 8.561866371093706e-93
i: 2
$\delta_{70}(S_2)$ = emi(70,2) * max([δ_{70-1} * (S_2) * trans(2,69)])
$\delta_{70}(S_2)$ = 0.02129 * 8.879245119705786e-93 = 1.8903912859853617e-94
i: 3
$\delta_{70}(S_3)$ = emi(70,3) * max([δ_{70-1} * (S_3) * trans(3,69)])
$\delta_{70}(S_3)$ = 0.00782 * 2.663773535911736e-91 = 2.0830709050829773e-93

T: 71

i: 1
$\delta_{71}(S_1)$ = emi(71,1) * max([δ_{71-1} * (S_1) * trans(1,70)])
$\delta_{71}(S_1)$ = 0.05171 * 8.305010379960896e-93 = 4.294520867477779e-94
i: 2
$\delta_{71}(S_2)$ = emi(71,2) * max([δ_{71-1} * (S_2) * trans(2,70)])
$\delta_{71}(S_2)$ = 0.1033 * 1.134234771591217e-94 = 1.1716645190537272e-95
i: 3
$\delta_{71}(S_3)$ = emi(71,3) * max([δ_{71-1} * (S_3) * trans(3,70)])
$\delta_{71}(S_3)$ = 0.19429 * 1.8747638145746797e-93 = 3.642478615337145e-94

T: 72

i: 1
$\delta_{72}(S_1)$ = emi(72,1) * max([δ_{72-1} * (S_1) * trans(1,71)])
$\delta_{72}(S_1)$ = 0.05092 * 4.165685241453446e-94 = 2.1211669249480948e-95
i: 2
$\delta_{72}(S_2)$ = emi(72,2) * max([δ_{72-1} * (S_2) * trans(2,71)])
$\delta_{72}(S_2)$ = 0.03962 * 1.0927435846011433e-95 = 4.3294500821897303e-97
i: 3
$\delta_{72}(S_3)$ = emi(72,3) * max([δ_{72-1} * (S_3) * trans(3,71)])
$\delta_{72}(S_3)$ = 0.00687 * 3.2782307538034304e-94 = 2.2521445278629567e-96

T: 73

i: 1
$\delta_{73}(S_1)$ = emi(73,1) * max([δ_{73-1} * (S_1) * trans(1,72)])
$\delta_{73}(S_1)$ = 0.04621 * 2.057531917199652e-95 = 9.50785498937959e-97
i: 2
$\delta_{73}(S_2)$ = emi(73,2) * max([δ_{73-1} * (S_2) * trans(2,72)])
$\delta_{73}(S_2)$ = 0.05409 * 2.597670049313838e-97 = 1.405079729673855e-98
i: 3
$\delta_{73}(S_3)$ = emi(73,3) * max([δ_{73-1} * (S_3) * trans(3,72)])
$\delta_{73}(S_3)$ = 0.00457 * 2.026930075076661e-96 = 9.263070443100341e-99

T: 74

i: 1
$\delta_{74}(S_1)$ = emi(74,1) * max([δ_{74-1} * (S_1) * trans(1,73)])
$\delta_{74}(S_1)$ = 0.09042 * 9.222619339698203e-97 = 8.339092406955116e-98
i: 2
$\delta_{74}(S_2)$ = emi(74,2) * max([δ_{74-1} * (S_2) * trans(2,73)])
$\delta_{74}(S_2)$ = 0.05638 * 8.43047837804313e-99 = 4.753103709540717e-100
i: 3
$\delta_{74}(S_3)$ = emi(74,3) * max([δ_{74-1} * (S_3) * trans(3,73)])
$\delta_{74}(S_3)$ = 0.00398 * 2.852356496813877e-98 = 1.135237885731923e-100

T: 75

i: 1
$\delta_{75}(S_1)$ = emi(75,1) * max([δ_{75-1} * (S_1) * trans(1,74)])
$\delta_{75}(S_1)$ = 0.05877 * 8.088919634746462e-98 = 4.753858069340496e-99
i: 2
$\delta_{75}(S_2)$ = emi(75,2) * max([δ_{75-1} * (S_2) * trans(2,74)])
$\delta_{75}(S_2)$ = 0.04271 * 2.85186222572443e-100 = 1.218030356606904e-101
i: 3
$\delta_{75}(S_3)$ = emi(75,3) * max([δ_{75-1} * (S_3) * trans(3,74)])
$\delta_{75}(S_3)$ = 0.01879 * 2.5017277220865348e-99 = 4.700746389800599e-101

T: 76

i: 1
$\delta_{76}(S_1)$ = emi(76,1) * max([δ_{76-1} * (S_1) * trans(1,75)])
$\delta_{76}(S_1)$ = 0.01482 * 4.611242327260281e-99 = 6.833861128999736e-101
i: 2
$\delta_{76}(S_2)$ = emi(76,2) * max([δ_{76-1} * (S_2) * trans(2,75)])
$\delta_{76}(S_2)$ = 0.01439 * 7.308182139641423e-102 = 1.0516474098944007e-103
i: 3
$\delta_{76}(S_3)$ = emi(76,3) * max([δ_{76-1} * (S_3) * trans(3,75)])
$\delta_{76}(S_3)$ = 0.04444 * 1.4261574208021487e-100 = 6.337843578044749e-102

T: 77

i: 1
$\delta_{77}(S_1)$ = emi(77,1) * max([δ_{77-1} * (S_1) * trans(1,76)])
$\delta_{77}(S_1)$ = 0.06409 * 6.628845295129744e-101 = 4.2484269496486525e-102
i: 2
$\delta_{77}(S_2)$ = emi(77,2) * max([δ_{77-1} * (S_2) * trans(2,76)])
$\delta_{77}(S_2)$ = 0.06927 * 1.9013530734134246e-103 = 1.3170672739534792e-104
i: 3
$\delta_{77}(S_3)$ = emi(77,3) * max([δ_{77-1} * (S_3) * trans(3,76)])
$\delta_{77}(S_3)$ = 0.04995 * 5.704059220240274e-102 = 2.849177580510017e-103

T: 78

i: 1
$\delta_{78}(S_1)$ = emi(78,1) * max([δ_{78-1} * (S_1) * trans(1,77)])
$\delta_{78}(S_1)$ = 0.06901 * 4.120974141159193e-102 = 2.8438842548139593e-103
i: 2
$\delta_{78}(S_2)$ = emi(78,2) * max([δ_{78-1} * (S_2) * trans(2,77)])
$\delta_{78}(S_2)$ = 0.07052 * 8.547532741530052e-105 = 6.027720089326992e-106
i: 3
$\delta_{78}(S_3)$ = emi(78,3) * max([δ_{78-1} * (S_3) * trans(3,77)])
$\delta_{78}(S_3)$ = 0.09536 * 2.5642598224590153e-103 = 2.445278166696917e-104

T: 79

i: 1
$\delta_{79}(S_1)$ = emi(79,1) * max([δ_{79-1} * (S_1) * trans(1,78)])
$\delta_{79}(S_1)$ = 0.05171 * 2.7585677271695404e-103 = 1.4264553717193694e-104
i: 2
$\delta_{79}(S_2)$ = emi(79,2) * max([δ_{79-1} * (S_2) * trans(2,78)])
$\delta_{79}(S_2)$ = 0.1033 * 7.335834500090751e-106 = 7.577917038593747e-107
i: 3
$\delta_{79}(S_3)$ = emi(79,3) * max([δ_{79-1} * (S_3) * trans(3,78)])

$\delta_{79}(S_3)$ = 0.19429 * 2.2007503500272253e-104 = 4.2758378550678955e-105

T: 80

i: 1
$\delta_{80}(S_1)$ = emi(80,1) * max([δ_{80-1} * (S_1) * trans(1,79)])
$\delta_{80}(S_1)$ = 0.05171 * 1.3836617105677883e-104 = 7.154914705346034e-106
i: 2
$\delta_{80}(S_2)$ = emi(80,2) * max([δ_{80-1} * (S_2) * trans(2,79)])
$\delta_{80}(S_2)$ = 0.1033 * 1.2827513565203687e-106 = 1.3250821512855409e-107
i: 3
$\delta_{80}(S_3)$ = emi(80,3) * max([δ_{80-1} * (S_3) * trans(3,79)])
$\delta_{80}(S_3)$ = 0.19429 * 3.848254069561106e-105 = 7.476772831750273e-106

T: 81

i: 1
$\delta_{81}(S_1)$ = emi(81,1) * max([δ_{81-1} * (S_1) * trans(1,80)])
$\delta_{81}(S_1)$ = 0.06521 * 6.940267264185653e-106 = 4.525748282975465e-107
i: 2
$\delta_{81}(S_2)$ = emi(81,2) * max([δ_{81-1} * (S_2) * trans(2,80)])
$\delta_{81}(S_2)$ = 0.05586 * 2.2430318495250817e-107 = 1.2529575911447106e-108
i: 3
$\delta_{81}(S_3)$ = emi(81,3) * max([δ_{81-1} * (S_3) * trans(3,80)])
$\delta_{81}(S_3)$ = 0.02246 * 6.729095548575246e-106 = 1.5113548602100002e-107

T: 82

i: 1
$\delta_{82}(S_1)$ = emi(82,1) * max([δ_{82-1} * (S_1) * trans(1,81)])
$\delta_{82}(S_1)$ = 0.06901 * 4.389975834486201e-107 = 3.029522323378927e-108
i: 2
$\delta_{82}(S_2)$ = emi(82,2) * max([δ_{82-1} * (S_2) * trans(2,81)])
$\delta_{82}(S_2)$ = 0.07052 * 7.517745546868263e-109 = 5.301514159651499e-110
i: 3
$\delta_{82}(S_3)$ = emi(82,3) * max([δ_{82-1} * (S_3) * trans(3,81)])
$\delta_{82}(S_3)$ = 0.09536 * 1.3602193741890002e-107 = 1.2971051952266307e-108

T: 83

i: 1
$\delta_{83}(S_1)$ = emi(83,1) * max([δ_{83-1} * (S_1) * trans(1,82)])
$\delta_{83}(S_1)$ = 0.04595 * 2.9386366536775593e-108 = 1.3503035423648385e-109
i: 2
$\delta_{83}(S_2)$ = emi(83,2) * max([δ_{83-1} * (S_2) * trans(2,82)])
$\delta_{83}(S_2)$ = 0.04801 * 3.891315585679892e-110 = 1.868220612684916e-111
i: 3
$\delta_{83}(S_3)$ = emi(83,3) * max([δ_{83-1} * (S_3) * trans(3,82)])
$\delta_{83}(S_3)$ = 0.14222 * 1.1673946757039676e-108 = 1.6602687077861828e-109

T: 84

i: 1
$\delta_{84}(S_1)$ = emi(84,1) * max([δ_{84-1} * (S_1) * trans(1,83)])
$\delta_{84}(S_1)$ = 0.04595 * 1.3097944360938932e-109 = 6.018505433851439e-111
i: 2
$\delta_{84}(S_2)$ = emi(84,2) * max([δ_{84-1} * (S_2) * trans(2,83)])
$\delta_{84}(S_2)$ = 0.04801 * 4.980806123358548e-111 = 2.391285019824439e-112
i: 3
$\delta_{84}(S_3)$ = emi(84,3) * max([δ_{84-1} * (S_3) * trans(3,83)])
$\delta_{84}(S_3)$ = 0.14222 * 1.4942418370075644e-109 = 2.1251107405921583e-110

T: 85

i: 1
$\delta_{85}(S_1)$ = emi(85,1) * max([δ_{85-1} * (S_1) * trans(1,84)])
$\delta_{85}(S_1)$ = 0.04294 * 5.837950270835896e-111 = 2.5068158462969334e-112
i: 2
$\delta_{85}(S_2)$ = emi(85,2) * max([δ_{85-1} * (S_2) * trans(2,84)])
$\delta_{85}(S_2)$ = 0.04257 * 6.375332221776475e-112 = 2.7139789268102455e-113
i: 3
$\delta_{85}(S_3)$ = emi(85,3) * max([δ_{85-1} * (S_3) * trans(3,84)])
$\delta_{85}(S_3)$ = 0.11935 * 1.9125996665329427e-110 = 2.282687702007067e-111

T: 86

i: 1
$\delta_{86}(S_1)$ = emi(86,1) * max([δ_{86-1} * (S_1) * trans(1,85)])
$\delta_{86}(S_1)$ = 0.04294 * 2.431611370908025e-112 = 1.044133922667906e-113
i: 2
$\delta_{86}(S_2)$ = emi(86,2) * max([δ_{86-1} * (S_2) * trans(2,85)])
$\delta_{86}(S_2)$ = 0.04257 * 6.848063106021201e-113 = 2.915220464233225e-114
i: 3

$\delta_{86}(S_3)$ = emi(86,3) * max([δ_{86-1} * (S_3) * trans(3,85)])
$\delta_{86}(S_3)$ = 0.11935 * 2.0544189318063604e-111 = 2.451948995110891e-112

T: 87

i: 1
$\delta_{87}(S_1)$ = emi(87,1) * max([δ_{87-1} * (S_1) * trans(1,86)])
$\delta_{87}(S_1)$ = 0.05171 * 1.716364296577624e-113 = 8.875319777602892e-115
i: 2
$\delta_{87}(S_2)$ = emi(87,2) * max([δ_{87-1} * (S_2) * trans(2,86)])
$\delta_{87}(S_2)$ = 0.1033 * 7.355846985332673e-114 = 7.598589935848651e-115
i: 3
$\delta_{87}(S_3)$ = emi(87,3) * max([δ_{87-1} * (S_3) * trans(3,86)])
$\delta_{87}(S_3)$ = 0.19429 * 2.206754095599802e-112 = 4.287502532340855e-113

T: 88

i: 1
$\delta_{88}(S_1)$ = emi(88,1) * max([δ_{88-1} * (S_1) * trans(1,87)])
$\delta_{88}(S_1)$ = 0.06521 * 3.0012517726385988e-114 = 1.9571162809376303e-115
i: 2
$\delta_{88}(S_2)$ = emi(88,2) * max([δ_{88-1} * (S_2) * trans(2,87)])
$\delta_{88}(S_2)$ = 0.05586 * 1.2862507597022564e-114 = 7.184996743696804e-116
i: 3
$\delta_{88}(S_3)$ = emi(88,3) * max([δ_{88-1} * (S_3) * trans(3,87)])
$\delta_{88}(S_3)$ = 0.02246 * 3.85875227910677e-113 = 8.666757618873805e-115

T: 89

i: 1
$\delta_{89}(S_1)$ = emi(89,1) * max([δ_{89-1} * (S_1) * trans(1,88)])
$\delta_{89}(S_1)$ = 0.04294 * 1.8984027925095014e-115 = 8.151741591035799e-117
i: 2
$\delta_{89}(S_2)$ = emi(89,2) * max([δ_{89-1} * (S_2) * trans(2,88)])
$\delta_{89}(S_2)$ = 0.04257 * 4.310998046218082e-116 = 1.8351918682750376e-117
i: 3
$\delta_{89}(S_3)$ = emi(89,3) * max([δ_{89-1} * (S_3) * trans(3,88)])
$\delta_{89}(S_3)$ = 0.11935 * 7.800081856986425e-115 = 9.309397696313298e-116

T: 90

i: 1
$\delta_{90}(S_1)$ = emi(90,1) * max([δ_{90-1} * (S_1) * trans(1,89)])
$\delta_{90}(S_1)$ = 0.06901 * 7.907189343304725e-117 = 5.4567513658145906e-118
i: 2
$\delta_{90}(S_2)$ = emi(90,2) * max([δ_{90-1} * (S_2) * trans(2,89)])
$\delta_{90}(S_2)$ = 0.07052 * 2.7928193088939893e-117 = 1.9694961766320413e-118
i: 3
$\delta_{90}(S_3)$ = emi(90,3) * max([δ_{90-1} * (S_3) * trans(3,89)])
$\delta_{90}(S_3)$ = 0.09536 * 8.378457926681968e-116 = 7.989697478883925e-117

T: 91

i: 1
$\delta_{91}(S_1)$ = emi(91,1) * max([δ_{91-1} * (S_1) * trans(1,90)])
$\delta_{91}(S_1)$ = 0.05591 * 5.592788235218748e-118 = 3.126927902310802e-119
i: 2
$\delta_{91}(S_2)$ = emi(91,2) * max([δ_{91-1} * (S_2) * trans(2,90)])
$\delta_{91}(S_2)$ = 0.07239 * 2.3969092436651775e-118 = 1.735122601489222e-119
i: 3
$\delta_{91}(S_3)$ = emi(91,3) * max([δ_{91-1} * (S_3) * trans(3,90)])
$\delta_{91}(S_3)$ = 0.06898 * 7.190727730995533e-117 = 4.960163988840719e-118

T: 92

i: 1
$\delta_{92}(S_1)$ = emi(92,1) * max([δ_{92-1} * (S_1) * trans(1,91)])
$\delta_{92}(S_1)$ = 0.05877 * 3.4721147921885034e-119 = 2.0405618633691834e-120
i: 2
$\delta_{92}(S_2)$ = emi(92,2) * max([δ_{92-1} * (S_2) * trans(2,91)])
$\delta_{92}(S_2)$ = 0.04271 * 1.4880491966522155e-119 = 6.355458118901612e-121
i: 3
$\delta_{92}(S_3)$ = emi(92,3) * max([δ_{92-1} * (S_3) * trans(3,91)])
$\delta_{92}(S_3)$ = 0.01879 * 4.464147589956647e-118 = 8.38813332152854e-120

T: 93

i: 1
$\delta_{93}(S_1)$ = emi(93,1) * max([δ_{93-1} * (S_1) * trans(1,92)])
$\delta_{93}(S_1)$ = 0.04294 * 1.979345007468108e-120 = 8.499307462068055e-122
i: 2
$\delta_{93}(S_2)$ = emi(93,2) * max([δ_{93-1} * (S_2) * trans(2,92)])
$\delta_{93}(S_2)$ = 0.04257 * 3.813274871340967e-121 = 1.6233111127298495e-122

i: 3
$\delta_{93}(S_3)$ = emi(93,3) * max([δ_{93-1} * (S_3) * trans(3,92)])
$\delta_{93}(S_3)$ = 0.11935 * 7.549319989375687e-120 = 9.010113407319883e-121

T: 94

i: 1
$\delta_{94}(S_1)$ = emi(94,1) * max([δ_{94-1} * (S_1) * trans(1,93)])
$\delta_{94}(S_1)$ = 0.05171 * 8.244328238206013e-122 = 4.26314213197633e-123
i: 2
$\delta_{94}(S_2)$ = emi(94,2) * max([δ_{94-1} * (S_2) * trans(2,93)])
$\delta_{94}(S_2)$ = 0.1033 * 2.703034022195965e-122 = 2.7922341449284317e-123
i: 3
$\delta_{94}(S_3)$ = emi(94,3) * max([δ_{94-1} * (S_3) * trans(3,93)])
$\delta_{94}(S_3)$ = 0.19429 * 8.109102066587895e-121 = 1.575517440517362e-121

T: 95

i: 1
$\delta_{95}(S_1)$ = emi(95,1) * max([δ_{95-1} * (S_1) * trans(1,94)])
$\delta_{95}(S_1)$ = 0.04595 * 1.1028622083621534e-122 = 5.067651847424095e-124
i: 2
$\delta_{95}(S_2)$ = emi(95,2) * max([δ_{95-1} * (S_2) * trans(2,94)])
$\delta_{95}(S_2)$ = 0.04801 * 4.7265523215520855e-123 = 2.269217769577156e-124
i: 3
$\delta_{95}(S_3)$ = emi(95,3) * max([δ_{95-1} * (S_3) * trans(3,94)])
$\delta_{95}(S_3)$ = 0.14222 * 1.4179656964656258e-121 = 2.0166308135134132e-122

T: 96

i: 1
$\delta_{96}(S_1)$ = emi(96,1) * max([δ_{96-1} * (S_1) * trans(1,95)])
$\delta_{96}(S_1)$ = 0.04294 * 1.4116415694593893e-123 = 6.061588899258618e-125
i: 2
$\delta_{96}(S_2)$ = emi(96,2) * max([δ_{96-1} * (S_2) * trans(2,95)])
$\delta_{96}(S_2)$ = 0.04257 * 6.0498924405402395e-124 = 2.5754392119379795e-125
i: 3
$\delta_{96}(S_3)$ = emi(96,3) * max([δ_{96-1} * (S_3) * trans(3,95)])
$\delta_{96}(S_3)$ = 0.11935 * 1.814967732162072e-122 = 2.1661639883354327e-123

T: 97

i: 1
$\delta_{97}(S_1)$ = emi(97,1) * max([δ_{97-1} * (S_1) * trans(1,96)])
$\delta_{97}(S_1)$ = 0.0421 * 1.5163147918348031e-124 = 6.383685273624521e-126
i: 2
$\delta_{97}(S_2)$ = emi(97,2) * max([δ_{97-1} * (S_2) * trans(2,96)])
$\delta_{97}(S_2)$ = 0.0408 * 6.498491965006297e-125 = 2.6513847217225697e-126
i: 3
$\delta_{97}(S_3)$ = emi(97,3) * max([δ_{97-1} * (S_3) * trans(3,96)])
$\delta_{97}(S_3)$ = 0.02568 * 1.9495475895018896e-123 = 5.006438209840852e-125

T: 98

i: 1
$\delta_{98}(S_1)$ = emi(98,1) * max([δ_{98-1} * (S_1) * trans(1,97)])
$\delta_{98}(S_1)$ = 0.06901 * 6.192174715415785e-126 = 4.2732197711084334e-127
i: 2
$\delta_{98}(S_2)$ = emi(98,2) * max([δ_{98-1} * (S_2) * trans(2,97)])
$\delta_{98}(S_2)$ = 0.07052 * 1.5908308330335417e-126 = 1.1218539034552535e-127
i: 3
$\delta_{98}(S_3)$ = emi(98,3) * max([δ_{98-1} * (S_3) * trans(3,97)])
$\delta_{98}(S_3)$ = 0.09536 * 4.505794388856767e-125 = 4.2967255292138134e-126

T: 99

i: 1
$\delta_{99}(S_1)$ = emi(99,1) * max([δ_{99-1} * (S_1) * trans(1,98)])
$\delta_{99}(S_1)$ = 0.04595 * 4.14502317797518e-127 = 1.9046381502795952e-128
i: 2
$\delta_{99}(S_2)$ = emi(99,2) * max([δ_{99-1} * (S_2) * trans(2,98)])
$\delta_{99}(S_2)$ = 0.04801 * 1.289017658764144e-127 = 6.188573779726656e-129
i: 3
$\delta_{99}(S_3)$ = emi(99,3) * max([δ_{99-1} * (S_3) * trans(3,98)])
$\delta_{99}(S_3)$ = 0.14222 * 3.8670529762924324e-126 = 5.499722742883098e-127

T: 100

i: 1
$\delta_{100}(S_1)$ = emi(100,1) * max([δ_{100-1} * (S_1) * trans(1,99)])
$\delta_{100}(S_1)$ = 0.06409 * 3.849805920018169e-128 = 2.467340614139644e-129
i: 2
$\delta_{100}(S_2)$ = emi(100,2) * max([δ_{100-1} * (S_2) * trans(2,99)])

$\delta_{100}(S_2)$ = 0.06927 * 1.6499168228649293e-128 = 1.1428973831985365e-129
i: 3
$\delta_{100}(S_3)$ = emi(100,3) * max([δ_{100-1} * (S_3) * trans(3,99)])
$\delta_{100}(S_3)$ = 0.04995 * 4.9497504685947886e-127 = 2.472400359063097e-128

Backtracking

1. Each cell shows the delta corresponding to the particular hidden state at a particular position of the observed sequence.
2. In green is shown the calculated backtracking.
3. The backtracking starts on letter (S) at the last position, going back to the first letter (M) at the first position.

Matrix of δ values

	M_1	E_2	I_3	L_4	C_5	E_6	D_7	N_8	T_9	S_{10}	L_{11}	S_{12}	S_{13}	I_{14}	P_{15}	N_{16}	S_{17}
**I**	0.037890	0.001698	0.000071	0.000004	0.000000	0.000000	0.000000	0.000000	0.000000	0.000000	0.000000	0.000000	0.000000	0.000000	0.000000	0.000000	0.000
**O**	0.004080	0.000132	0.000003	0.000000	0.000000	0.000000	0.000000	0.000000	0.000000	0.000000	0.000000	0.000000	0.000000	0.000000	0.000000	0.000000	0.000
**M**	0.000000	0.000007	0.000006	0.000001	0.000000	0.000000	0.000000	0.000000	0.000000	0.000000	0.000000	0.000000	0.000000	0.000000	0.000000	0.000000	0.000

Termination

$$P(O \cdot Q) = \max_i \left[\delta_L\left(S_i\right) \cdot a_{i0}\right]$$
$$q_L^* = \arg\max_i \left[\delta_L\left(S_i\right) \cdot a_{i0}\right]$$

The most probable sequence of hidden states is:
I I I I I I I I I I I I I I I I I I I I I I I I I I I I I I I I I I I I I I I I I I I I I I I I I I I I I I I I I I I I I I I I I I I I I I I I I I I M M M M M M M M M M M M M M M M M M M M M M M M M
P(Q|O) = 2.472400359063097e-128

Backward

Initialization

Probability that the position 2 (E) was emitted by hidden state M.
Forward data:
P(O: MEILCEDNTSLSSIPNSLMQVDGDSGLYRNDFNSRDANSSDASNWTIDGENRTNLSFEGYLPPTCLSILHLQEKNWSALLTAVVIILTIAGNILVIMAVS) = 7.698066770155403e-127

Initialization

$$\beta(L, k) = 1 \quad \forall k, k = 1..N$$

1. The length of observed sequence is: 100.
2. The probability of β in the last state will be 1.
3. There are 3 hidden states.

β(100,1) = 1
β(100,2) = 1
β(100,3) = 1

Recursion

$$\beta(t-1, i) = \sum_{j=1}^{N} a_{ij} \cdot e_{q_t = S_j}\left(O_t\right) \cdot \beta(t, j)$$

T: 100

i: 1
β(100-1,1) = Σ[trans(1,1) * emi(1,100) * β(100,1)][trans(1,2) * emi(2,100) * β(100,2)][trans(1,3) * emi(3,100) * β(100,3)]
β(99,1)[0.97 * 0.06409 * 1][0.0 * 0.06927 * 1][0.03 * 0.04995 * 1] + = 0.0636658
i: 2
β(100-1,2) = Σ[trans(1,1) * emi(1,99) * β(99,1)][trans(1,2) * emi(2,99) * β(99,2)][trans(1,3) * emi(3,99) * β(99,3)]
β(99,2)[0.0 * 0.06409 * 1][0.6 * 0.06927 * 1][0.4 * 0.04995 * 1] + = 0.061542
i: 3
β(100-1,3) = Σ[trans(1,1) * emi(1,98) * β(98,1)][trans(1,2) * emi(2,98) * β(98,2)][trans(1,3) * emi(3,98) * β(98,3)]

β(99,3)[0.07 * 0.06409 * 1][0.03 * 0.06927 * 1][0.9 * 0.04995 * 1] + = 0.0515194

T: 99

i: 1
β(99-1,1) = Σ[[trans(2,1) * emi(1,100) * β(100,1)][trans(2,2) * emi(2,100) * β(100,2)][trans(2,3) * emi(3,100) * β(100,3)]
β(98,1)[0.97 * 0.06409 * 0.0636658][0.0 * 0.06927 * 0.0636658][0.03 * 0.04995 * 0.0636658] + = 0.0030574928767399994
i: 2
β(99-1,2) = Σ[[trans(2,1) * emi(1,99) * β(99,1)][trans(2,2) * emi(2,99) * β(99,2)][trans(2,3) * emi(3,99) * β(99,3)]
β(98,2)[0.0 * 0.06409 * 0.061542][0.6 * 0.06927 * 0.061542][0.4 * 0.04995 * 0.061542] + = 0.0047036144792
i: 3
β(99-1,3) = Σ[[trans(2,1) * emi(1,98) * β(98,1)][trans(2,2) * emi(2,98) * β(98,2)][trans(2,3) * emi(3,98) * β(98,3)]
β(98,3)[0.07 * 0.06409 * 0.0515194][0.03 * 0.06927 * 0.0515194][0.9 * 0.04995 * 0.0515194] + = 0.006887800149500001

T: 98

i: 1
β(98-1,1) = Σ[[trans(3,1) * emi(1,100) * β(100,1)][trans(3,2) * emi(2,100) * β(100,2)][trans(3,3) * emi(3,100) * β(100,3)]
β(97,1)[0.97 * 0.04595 * 0.0030574928767399994][0.0 * 0.04801 * 0.0030574928767399994][0.03 * 0.14222 * 0.0030574928767399994] + = 0.00022437227458880213
i: 2
β(98-1,2) = Σ[[trans(3,1) * emi(1,99) * β(99,1)][trans(3,2) * emi(2,99) * β(99,2)][trans(3,3) * emi(3,99) * β(99,3)]
β(97,2)[0.0 * 0.04595 * 0.0047036144792][0.6 * 0.04801 * 0.0047036144792][0.4 * 0.14222 * 0.0047036144792] + = 0.0004617475847464385
i: 3
β(98-1,3) = Σ[[trans(3,1) * emi(1,98) * β(98,1)][trans(3,2) * emi(2,98) * β(98,2)][trans(3,3) * emi(3,98) * β(98,3)]
β(97,3)[0.07 * 0.04595 * 0.006887800149500001][0.03 * 0.04801 * 0.006887800149500001][0.9 * 0.14222 * 0.006887800149500001] + = 0.0006158593576625516

T: 97

i: 1
β(97-1,1) = Σ[[trans(4,1) * emi(1,100) * β(100,1)][trans(4,2) * emi(2,100) * β(100,2)][trans(4,3) * emi(3,100) * β(100,3)]
β(96,1)[0.97 * 0.06901 * 0.00022437227458880213][0.0 * 0.07052 * 0.00022437227458880213][0.03 * 0.09536 * 0.00022437227458880213] + = 9.637148626526142e-06
i: 2
β(97-1,2) = Σ[[trans(4,1) * emi(1,99) * β(99,1)][trans(4,2) * emi(2,99) * β(99,2)][trans(4,3) * emi(3,99) * β(99,3)]
β(96,2)[0.0 * 0.06901 * 0.0004617475847464385][0.6 * 0.07052 * 0.0004617475847464385][0.4 * 0.09536 * 0.0004617475847464385] + = 1.7629688196502545e-05
i: 3
β(97-1,3) = Σ[[trans(4,1) * emi(1,98) * β(98,1)][trans(4,2) * emi(2,98) * β(98,2)][trans(4,3) * emi(3,98) * β(98,3)]
β(96,3)[0.07 * 0.06901 * 0.0006158593576625516][0.03 * 0.07052 * 0.0006158593576625516][0.9 * 0.09536 * 0.0006158593576625516] + = 1.546014561123973e-05

T: 96

i: 1
β(96-1,1) = Σ[[trans(5,1) * emi(1,100) * β(100,1)][trans(5,2) * emi(2,100) * β(100,2)][trans(5,3) * emi(3,100) * β(100,3)]
β(95,1)[0.97 * 0.0421 * 9.637148626526142e-06][0.0 * 0.0408 * 9.637148626526142e-06][0.03 * 0.02568 * 9.637148626526142e-06] + = 4.5675963852338534e-07
i: 2
β(96-1,2) = Σ[[trans(5,1) * emi(1,99) * β(99,1)][trans(5,2) * emi(2,99) * β(99,2)][trans(5,3) * emi(3,99) * β(99,3)]
β(95,2)[0.0 * 0.0421 * 1.7629688196502545e-05][0.6 * 0.0408 * 1.7629688196502545e-05][0.4 * 0.02568 * 1.7629688196502545e-05] + = 1.1883648473956529e-06
i: 3
β(96-1,3) = Σ[[trans(5,1) * emi(1,98) * β(98,1)][trans(5,2) * emi(2,98) * β(98,2)][trans(5,3) * emi(3,98) * β(98,3)]
β(95,3)[0.07 * 0.0421 * 1.546014561123973e-05][0.03 * 0.0408 * 1.546014561123973e-05][0.9 * 0.02568 * 1.546014561123973e-05] + = 1.7121337569686812e-06

T: 95

i: 1
β(95-1,1) = Σ[[trans(6,1) * emi(1,100) * β(100,1)][trans(6,2) * emi(2,100) * β(100,2)][trans(6,3) * emi(3,100) * β(100,3)]
β(94,1)[0.97 * 0.04294 * 4.5675963852338534e-07][0.0 * 0.04257 * 4.5675963852338534e-07][0.03 * 0.11935 * 4.5675963852338534e-07] + = 2.7663452115927645e-08
i: 2
β(95-1,2) = Σ[[trans(6,1) * emi(1,99) * β(99,1)][trans(6,2) * emi(2,99) * β(99,2)][trans(6,3) * emi(3,99) * β(99,3)]
β(94,2)[0.0 * 0.04294 * 1.1883648473956529e-06][0.6 * 0.04257 * 1.1883648473956529e-06][0.4 * 0.11935 * 1.1883648473956529e-06] + = 1.3163190296051354e-07
i: 3
β(95-1,3) = Σ[[trans(6,1) * emi(1,98) * β(98,1)][trans(6,2) * emi(2,98) * β(98,2)][trans(6,3) * emi(3,98) * β(98,3)]
β(94,3)[0.07 * 0.04294 * 1.7121337569686812e-06][0.03 * 0.04257 * 1.7121337569686812e-06][0.9 * 0.11935 * 1.7121337569686812e-06] + = 2.2233046589149172e-07

T: 94

i: 1
β(94-1,1) = Σ[[trans(7,1) * emi(1,100) * β(100,1)][trans(7,2) * emi(2,100) * β(100,2)][trans(7,3) * emi(3,100) * β(100,3)]
β(93,1)[0.97 * 0.04595 * 2.7663452115927645e-08][0.0 * 0.04801 * 2.7663452115927645e-08][0.03 * 0.14222 * 2.7663452115927645e-08] + = 2.6834603821889174e-09
i: 2
β(94-1,2) = Σ[[trans(7,1) * emi(1,99) * β(99,1)][trans(7,2) * emi(2,99) * β(99,2)][trans(7,3) * emi(3,99) * β(99,3)]
β(93,2)[0.0 * 0.04595 * 1.3163190296051354e-07][0.6 * 0.04801 * 1.3163190296051354e-07][0.4 * 0.14222 * 1.3163190296051354e-07] + = 2.54371798327158e-08
i: 3
β(94-1,3) = Σ[[trans(7,1) * emi(1,98) * β(98,1)][trans(7,2) * emi(2,98) * β(98,2)][trans(7,3) * emi(3,98) * β(98,3)]
β(93,3)[0.07 * 0.04595 * 2.2233046589149172e-07][0.03 * 0.04801 * 2.2233046589149172e-07][0.9 * 0.14222 * 2.2233046589149172e-07] + = 3.938498826115079e-08

T: 93

i: 1
β(93-1,1) = Σ[[trans(8,1) * emi(1,100) * β(100,1)][trans(8,2) * emi(2,100) * β(100,2)][trans(8,3) * emi(3,100) * β(100,3)]
β(92,1)[0.97 * 0.05171 * 2.6834603821889174e-09][0.0 * 0.1033 * 2.6834603821889174e-09][0.03 * 0.19429 * 2.6834603821889174e-09] + = 2.5278890561590674e-10
i: 2
β(93-1,2) = Σ[[trans(8,1) * emi(1,99) * β(99,1)][trans(8,2) * emi(2,99) * β(99,2)][trans(8,3) * emi(3,99) * β(99,3)]
β(92,2)[0.0 * 0.05171 * 2.54371798327158e-08][0.6 * 0.1033 * 2.54371798327158e-08][0.4 * 0.19429 * 2.54371798327158e-08] + = 2.5299557868745658e-09
i: 3

β(93-1,3) = Σ[[trans(8,1) * emi(1,98) * β(98,1)][trans(8,2) * emi(2,98) * β(98,2)][trans(8,3) * emi(3,98) * β(98,3)]
β(92,3)[0.07 * 0.05171 * 3.938498826115079e-08][0.03 * 0.1033 * 3.938498826115079e-08][0.9 * 0.19429 * 3.938498826115079e-08] + = 4.271090281652656e-09

T: 92

i: 1
β(92-1,1) = Σ[[trans(9,1) * emi(1,100) * β(100,1)][trans(9,2) * emi(2,100) * β(100,2)][trans(9,3) * emi(3,100) * β(100,3)]
β(91,1)[0.97 * 0.04294 * 2.5278890561590674e-10][0.0 * 0.04257 * 2.5278890561590674e-10][0.03 * 0.11935 * 2.5278890561590674e-10] + = 1.6818325455323037e-11
i: 2
β(92-1,2) = Σ[[trans(9,1) * emi(1,99) * β(99,1)][trans(9,2) * emi(2,99) * β(99,2)][trans(9,3) * emi(3,99) * β(99,3)]
β(91,2)[0.0 * 0.04294 * 2.5299557868745658e-09][0.6 * 0.04257 * 2.5299557868745658e-09][0.4 * 0.11935 * 2.5299557868745658e-09] + = 9.693416155134899e-11
i: 3
β(92-1,3) = Σ[[trans(9,1) * emi(1,98) * β(98,1)][trans(9,2) * emi(2,98) * β(98,2)][trans(9,3) * emi(3,98) * β(98,3)]
β(91,3)[0.07 * 0.04294 * 4.271090281652656e-09][0.03 * 0.04257 * 4.271090281652656e-09][0.9 * 0.11935 * 4.271090281652656e-09] + = 7.650998838156374e-11

T: 91

i: 1
β(91-1,1) = Σ[[trans(10,1) * emi(1,100) * β(100,1)][trans(10,2) * emi(2,100) * β(100,2)][trans(10,3) * emi(3,100) * β(100,3)]
β(90,1)[0.97 * 0.05877 * 1.6818325455323037e-11][0.0 * 0.04271 * 1.6818325455323037e-11][0.03 * 0.01879 * 1.6818325455323037e-11] + = 1.070432968877706e-12
i: 2
β(91-1,2) = Σ[[trans(10,1) * emi(1,99) * β(99,1)][trans(10,2) * emi(2,99) * β(99,2)][trans(10,3) * emi(3,99) * β(99,3)]
β(90,2)[0.0 * 0.05877 * 9.693416155134899e-11][0.6 * 0.04271 * 9.693416155134899e-11][0.4 * 0.01879 * 9.693416155134899e-11] + = 6.321301972245398e-12
i: 3
β(91-1,3) = Σ[[trans(10,1) * emi(1,98) * β(98,1)][trans(10,2) * emi(2,98) * β(98,2)][trans(10,3) * emi(3,98) * β(98,3)]
β(90,3)[0.07 * 0.05877 * 7.650998838156374e-11][0.03 * 0.04271 * 7.650998838156374e-11][0.9 * 0.01879 * 7.650998838156374e-11] + = 5.026226897679803e-12

T: 90

i: 1
β(90-1,1) = Σ[[trans(11,1) * emi(1,100) * β(100,1)][trans(11,2) * emi(2,100) * β(100,2)][trans(11,3) * emi(3,100) * β(100,3)]
β(89,1)[0.97 * 0.05591 * 1.070432968877706e-12][0.0 * 0.07239 * 1.070432968877706e-12][0.03 * 0.06898 * 1.070432968877706e-12] + = 8.603349171566536e-14
i: 2
β(90-1,2) = Σ[[trans(11,1) * emi(1,99) * β(99,1)][trans(11,2) * emi(2,99) * β(99,2)][trans(11,3) * emi(3,99) * β(99,3)]
β(89,2)[0.0 * 0.05591 * 6.321301972245398e-12][0.6 * 0.07239 * 6.321301972245398e-12][0.4 * 0.06898 * 6.321301972245398e-12] + = 4.591873278347457e-13
i: 3
β(90-1,3) = Σ[[trans(11,1) * emi(1,98) * β(98,1)][trans(11,2) * emi(2,98) * β(98,2)][trans(11,3) * emi(3,98) * β(98,3)]
β(89,3)[0.07 * 0.05591 * 5.026226897679803e-12][0.03 * 0.07239 * 5.026226897679803e-12][0.9 * 0.06898 * 5.026226897679803e-12] + = 4.4991518426171126e-13

T: 89

i: 1
β(89-1,1) = Σ[[trans(12,1) * emi(1,100) * β(100,1)][trans(12,2) * emi(2,100) * β(100,2)][trans(12,3) * emi(3,100) * β(100,3)]
β(88,1)[0.97 * 0.06901 * 8.603349171566536e-14][0.0 * 0.07052 * 8.603349171566536e-14][0.03 * 0.09536 * 8.603349171566536e-14] + = 5.1943711074916075e-15
i: 2
β(89-1,2) = Σ[[trans(12,1) * emi(1,99) * β(99,1)][trans(12,2) * emi(2,99) * β(99,2)][trans(12,3) * emi(3,99) * β(99,3)]
β(88,2)[0.0 * 0.06901 * 4.591873278347457e-13][0.6 * 0.07052 * 4.591873278347457e-13][0.4 * 0.09536 * 4.591873278347457e-13] + = 3.320751362420917e-14
i: 3
β(89-1,3) = Σ[[trans(12,1) * emi(1,98) * β(98,1)][trans(12,2) * emi(2,98) * β(98,2)][trans(12,3) * emi(3,98) * β(98,3)]
β(88,3)[0.07 * 0.06901 * 4.4991518426171126e-13][0.03 * 0.07052 * 4.4991518426171126e-13][0.9 * 0.09536 * 4.4991518426171126e-13] + = 4.917266712324841e-14

T: 88

i: 1
β(88-1,1) = Σ[[trans(13,1) * emi(1,100) * β(100,1)][trans(13,2) * emi(2,100) * β(100,2)][trans(13,3) * emi(3,100) * β(100,3)]
β(87,1)[0.97 * 0.04294 * 5.1943711074916075e-15][0.0 * 0.04257 * 5.1943711074916075e-15][0.03 * 0.11935 * 5.1943711074916075e-15] + = 3.6169573482958664e-16
i: 2
β(88-1,2) = Σ[[trans(13,1) * emi(1,99) * β(99,1)][trans(13,2) * emi(2,99) * β(99,2)][trans(13,3) * emi(3,99) * β(99,3)]
β(87,2)[0.0 * 0.04294 * 3.320751362420917e-14][0.6 * 0.04257 * 3.320751362420917e-14][0.4 * 0.11935 * 3.320751362420917e-14] + = 1.554750268064258e-15
i: 3
β(88-1,3) = Σ[[trans(13,1) * emi(1,98) * β(98,1)][trans(13,2) * emi(2,98) * β(98,2)][trans(13,3) * emi(3,98) * β(98,3)]
β(87,3)[0.07 * 0.04294 * 4.917266712324841e-14][0.03 * 0.04257 * 4.917266712324841e-14][0.9 * 0.11935 * 4.917266712324841e-14] + = 1.07333619035516e-15

T: 87

i: 1
β(87-1,1) = Σ[[trans(14,1) * emi(1,100) * β(100,1)][trans(14,2) * emi(2,100) * β(100,2)][trans(14,3) * emi(3,100) * β(100,3)]
β(86,1)[0.97 * 0.06521 * 3.6169573482958664e-16][0.0 * 0.05586 * 3.6169573482958664e-16][0.03 * 0.02246 * 3.6169573482958664e-16] + = 2.4398342507319907e-17
i: 2
β(87-1,2) = Σ[[trans(14,1) * emi(1,99) * β(99,1)][trans(14,2) * emi(2,99) * β(99,2)][trans(14,3) * emi(3,99) * β(99,3)]
β(86,2)[0.0 * 0.06521 * 1.554750268064258e-15][0.6 * 0.05586 * 1.554750268064258e-15][0.4 * 0.02246 * 1.554750268064258e-15] + = 1.7977881698426434e-16
i: 3
β(87-1,3) = Σ[[trans(14,1) * emi(1,98) * β(98,1)][trans(14,2) * emi(2,98) * β(98,2)][trans(14,3) * emi(3,98) * β(98,3)]
β(86,3)[0.07 * 0.06521 * 1.07333619035516e-15][0.03 * 0.05586 * 1.07333619035516e-15][0.9 * 0.02246 * 1.07333619035516e-15] + = 1.9381204071378742e-16

T: 86

i: 1
β(86-1,1) = Σ[[trans(15,1) * emi(1,100) * β(100,1)][trans(15,2) * emi(2,100) * β(100,2)][trans(15,3) * emi(3,100) * β(100,3)]
β(85,1)[0.97 * 0.05171 * 2.4398342507319907e-17][0.0 * 0.1033 * 2.4398342507319907e-17][0.03 * 0.19429 * 2.4398342507319907e-17] + = 1.710178894222103e-18
i: 2
β(86-1,2) = Σ[[trans(15,1) * emi(1,99) * β(99,1)][trans(15,2) * emi(2,99) * β(99,2)][trans(15,3) * emi(3,99) * β(99,3)]
β(85,2)[0.0 * 0.05171 * 1.7977881698426434e-16][0.6 * 0.1033 * 1.7977881698426434e-16][0.4 * 0.19429 * 1.7977881698426434e-16] + = 1.384449736708829e-17

i: 3
β(86-1,3) = Σ[[trans(15,1) * emi(1,98) * β(98,1)][trans(15,2) * emi(2,98) * β(98,2)][trans(15,3) * emi(3,98) * β(98,3)]
β(85,3)[0.07 * 0.05171 * 1.9381204071378742e-16][0.03 * 0.1033 * 1.9381204071378742e-16][0.9 * 0.19429 * 1.9381204071378742e-16] + = 2.112125241835058e-17

T: 85

i: 1
β(85-1,1) = Σ[[trans(16,1) * emi(1,100) * β(100,1)][trans(16,2) * emi(2,100) * β(100,2)][trans(16,3) * emi(3,100) * β(100,3)]
β(84,1)[0.97 * 0.04294 * 1.710178894222103e-18][0.0 * 0.04257 * 1.710178894222103e-18][0.03 * 0.11935 * 1.710178894222103e-18] + = 1.4685667355026444e-19
i: 2
β(85-1,2) = Σ[[trans(16,1) * emi(1,99) * β(99,1)][trans(16,2) * emi(2,99) * β(99,2)][trans(16,3) * emi(3,99) * β(99,3)]
β(84,2)[0.0 * 0.04294 * 1.384449736708829e-17][0.6 * 0.04257 * 1.384449736708829e-17][0.4 * 0.11935 * 1.384449736708829e-17] + = 1.3619447422022259e-18
i: 3
β(85-1,3) = Σ[[trans(16,1) * emi(1,98) * β(98,1)][trans(16,2) * emi(2,98) * β(98,2)][trans(16,3) * emi(3,98) * β(98,3)]
β(84,3)[0.07 * 0.04294 * 2.112125241835058e-17][0.03 * 0.04257 * 2.112125241835058e-17][0.9 * 0.11935 * 2.112125241835058e-17] + = 2.2915605918248887e-18

T: 84

i: 1
β(84-1,1) = Σ[[trans(17,1) * emi(1,100) * β(100,1)][trans(17,2) * emi(2,100) * β(100,2)][trans(17,3) * emi(3,100) * β(100,3)]
β(83,1)[0.97 * 0.04294 * 1.4685667355026444e-19][0.0 * 0.04257 * 1.4685667355026444e-19][0.03 * 0.11935 * 1.4685667355026444e-19] + = 1.6322794646225683e-20
i: 2
β(84-1,2) = Σ[[trans(17,1) * emi(1,99) * β(99,1)][trans(17,2) * emi(2,99) * β(99,2)][trans(17,3) * emi(3,99) * β(99,3)]
β(83,2)[0.0 * 0.04294 * 1.3619447422022259e-18][0.6 * 0.04257 * 1.3619447422022259e-18][0.4 * 0.11935 * 1.3619447422022259e-18] + = 1.6959447919161158e-19
i: 3
β(84-1,3) = Σ[[trans(17,1) * emi(1,98) * β(98,1)][trans(17,2) * emi(2,98) * β(98,2)][trans(17,3) * emi(3,98) * β(98,3)]
β(83,3)[0.07 * 0.04294 * 2.2915605918248887e-18][0.03 * 0.04257 * 2.2915605918248887e-18][0.9 * 0.11935 * 2.2915605918248887e-18] + = 2.9574914613507046e-19

T: 83

i: 1
β(83-1,1) = Σ[[trans(18,1) * emi(1,100) * β(100,1)][trans(18,2) * emi(2,100) * β(100,2)][trans(18,3) * emi(3,100) * β(100,3)]
β(82,1)[0.97 * 0.04595 * 1.6322794646225683e-20][0.0 * 0.04801 * 1.6322794646225683e-20][0.03 * 0.14222 * 1.6322794646225683e-20] + = 1.98937474847414e-21
i: 2
β(83-1,2) = Σ[[trans(18,1) * emi(1,99) * β(99,1)][trans(18,2) * emi(2,99) * β(99,2)][trans(18,3) * emi(3,99) * β(99,3)]
β(82,2)[0.0 * 0.04595 * 1.6959447919161158e-19][0.6 * 0.04801 * 1.6959447919161158e-19][0.4 * 0.14222 * 1.6959447919161158e-19] + = 2.1709915992925453e-20
i: 3
β(83-1,3) = Σ[[trans(18,1) * emi(1,98) * β(98,1)][trans(18,2) * emi(2,98) * β(98,2)][trans(18,3) * emi(3,98) * β(98,3)]
β(82,3)[0.07 * 0.04595 * 2.9574914613507046e-19][0.03 * 0.04801 * 2.9574914613507046e-19][0.9 * 0.14222 * 2.9574914613507046e-19] + = 3.815206840435602e-20

T: 82

i: 1
β(82-1,1) = Σ[[trans(19,1) * emi(1,100) * β(100,1)][trans(19,2) * emi(2,100) * β(100,2)][trans(19,3) * emi(3,100) * β(100,3)]
β(81,1)[0.97 * 0.04595 * 1.98937474847414e-21][0.0 * 0.04801 * 1.98937474847414e-21][0.03 * 0.14222 * 1.98937474847414e-21] + = 2.4231358614161606e-22
i: 2
β(82-1,2) = Σ[[trans(19,1) * emi(1,99) * β(99,1)][trans(19,2) * emi(2,99) * β(99,2)][trans(19,3) * emi(3,99) * β(99,3)]
β(81,2)[0.0 * 0.04595 * 2.1709915992925453e-20][0.6 * 0.04801 * 2.1709915992925453e-20][0.4 * 0.14222 * 2.1709915992925453e-20] + = 2.373862462708418e-21
i: 3
β(82-1,3) = Σ[[trans(19,1) * emi(1,98) * β(98,1)][trans(19,2) * emi(2,98) * β(98,2)][trans(19,3) * emi(3,98) * β(98,3)]
β(81,3)[0.07 * 0.04595 * 3.815206840435602e-20][0.03 * 0.04801 * 3.815206840435602e-20][0.9 * 0.14222 * 3.815206840435602e-20] + = 3.329902689607538e-21

T: 81

i: 1
β(81-1,1) = Σ[[trans(20,1) * emi(1,100) * β(100,1)][trans(20,2) * emi(2,100) * β(100,2)][trans(20,3) * emi(3,100) * β(100,3)]
β(80,1)[0.97 * 0.06901 * 2.4231358614161606e-22][0.0 * 0.07052 * 2.4231358614161606e-22][0.03 * 0.09536 * 2.4231358614161606e-22] + = 1.75709193159835e-23
i: 2
β(81-1,2) = Σ[[trans(20,1) * emi(1,99) * β(99,1)][trans(20,2) * emi(2,99) * β(99,2)][trans(20,3) * emi(3,99) * β(99,3)]
β(80,2)[0.0 * 0.06901 * 2.373862462708418e-21][0.6 * 0.07052 * 2.373862462708418e-21][0.4 * 0.09536 * 2.373862462708418e-21] + = 1.0947822006356944e-22
i: 3
β(81-1,3) = Σ[[trans(20,1) * emi(1,98) * β(98,1)][trans(20,2) * emi(2,98) * β(98,2)][trans(20,3) * emi(3,98) * β(98,3)]
β(80,3)[0.07 * 0.06901 * 3.329902689607538e-21][0.03 * 0.07052 * 3.329902689607538e-21][0.9 * 0.09536 * 3.329902689607538e-21] + = 7.239486050939418e-23

T: 80

i: 1
β(80-1,1) = Σ[[trans(21,1) * emi(1,100) * β(100,1)][trans(21,2) * emi(2,100) * β(100,2)][trans(21,3) * emi(3,100) * β(100,3)]
β(79,1)[0.97 * 0.06521 * 1.75709193159835e-23][0.0 * 0.05586 * 1.75709193159835e-23][0.03 * 0.02246 * 1.75709193159835e-23] + = 1.3033023941457275e-24
i: 2
β(80-1,2) = Σ[[trans(21,1) * emi(1,99) * β(99,1)][trans(21,2) * emi(2,99) * β(99,2)][trans(21,3) * emi(3,99) * β(99,3)]
β(79,2)[0.0 * 0.06521 * 1.0947822006356944e-22][0.6 * 0.05586 * 1.0947822006356944e-22][0.4 * 0.02246 * 1.0947822006356944e-22] + = 1.2411699058888112e-23
i: 3
β(80-1,3) = Σ[[trans(21,1) * emi(1,98) * β(98,1)][trans(21,2) * emi(2,98) * β(98,2)][trans(21,3) * emi(3,98) * β(98,3)]
β(79,3)[0.07 * 0.06521 * 7.239486050939418e-23][0.03 * 0.05586 * 7.239486050939418e-23][0.9 * 0.02246 * 7.239486050939418e-23] + = 1.3061912164158243e-23

T: 79

i: 1
β(79-1,1) = Σ[[trans(22,1) * emi(1,100) * β(100,1)][trans(22,2) * emi(2,100) * β(100,2)][trans(22,3) * emi(3,100) * β(100,3)]
β(78,1)[0.97 * 0.05171 * 1.3033023941457275e-24][0.0 * 0.1033 * 1.3033023941457275e-24][0.03 * 0.19429 * 1.3033023941457275e-24] + = 1.4150592122846645e-25
i: 2
β(79-1,2) = Σ[[trans(22,1) * emi(1,99) * β(99,1)][trans(22,2) * emi(2,99) * β(99,2)][trans(22,3) * emi(3,99) * β(99,3)]

β(78,2)[0.0 * 0.05171 * 1.2411699058888112e-23][0.6 * 0.1033 * 1.2411699058888112e-23][0.4 * 0.19429 * 1.2411699058888112e-23] + = 1.784396673419607e-24
i: 3
β(79-1,3) = Σ[[trans(22,1) * emi(1,98) * β(98,1)][trans(22,2) * emi(2,98) * β(98,2)][trans(22,3) * emi(3,98) * β(98,3)]
β(78,3)[0.07 * 0.05171 * 1.3061912164158243e-23][0.03 * 0.1033 * 1.3061912164158243e-23][0.9 * 0.19429 * 1.3061912164158243e-23] + = 2.327200441996458e-24

T: 78

i: 1
β(78-1,1) = Σ[[trans(23,1) * emi(1,100) * β(100,1)][trans(23,2) * emi(2,100) * β(100,2)][trans(23,3) * emi(3,100) * β(100,3)]
β(77,1)[0.97 * 0.05171 * 1.4150592122846645e-25][0.0 * 0.1033 * 1.4150592122846645e-25][0.03 * 0.19429 * 1.4150592122846645e-25] + = 1.6130018939720644e-26
i: 2
β(78-1,2) = Σ[[trans(23,1) * emi(1,99) * β(99,1)][trans(23,2) * emi(2,99) * β(99,2)][trans(23,3) * emi(3,99) * β(99,3)]
β(77,2)[0.0 * 0.05171 * 1.784396673419607e-24][0.6 * 0.1033 * 1.784396673419607e-24][0.4 * 0.19429 * 1.784396673419607e-24] + = 1.642701257052433e-25
i: 3
β(78-1,3) = Σ[[trans(23,1) * emi(1,98) * β(98,1)][trans(23,2) * emi(2,98) * β(98,2)][trans(23,3) * emi(3,98) * β(98,3)]
β(77,3)[0.07 * 0.05171 * 2.327200441996458e-24][0.03 * 0.1033 * 2.327200441996458e-24][0.9 * 0.19429 * 2.327200441996458e-24] + = 2.0418829298986888e-25

T: 77

i: 1
β(77-1,1) = Σ[[trans(24,1) * emi(1,100) * β(100,1)][trans(24,2) * emi(2,100) * β(100,2)][trans(24,3) * emi(3,100) * β(100,3)]
β(76,1)[0.97 * 0.06901 * 1.6130018939720644e-26][0.0 * 0.07052 * 1.6130018939720644e-26][0.03 * 0.09536 * 1.6130018939720644e-26] + = 1.3087358834766136e-27
i: 2
β(77-1,2) = Σ[[trans(24,1) * emi(1,99) * β(99,1)][trans(24,2) * emi(2,99) * β(99,2)][trans(24,3) * emi(3,99) * β(99,3)]
β(76,2)[0.0 * 0.06901 * 1.642701257052433e-25][0.6 * 0.07052 * 1.642701257052433e-25][0.4 * 0.09536 * 1.642701257052433e-25] + = 1.0907077058498902e-26
i: 3
β(77-1,3) = Σ[[trans(24,1) * emi(1,98) * β(98,1)][trans(24,2) * emi(2,98) * β(98,2)][trans(24,3) * emi(3,98) * β(98,3)]
β(76,3)[0.07 * 0.06901 * 2.0418829298986888e-25][0.03 * 0.07052 * 2.0418829298986888e-25][0.9 * 0.09536 * 2.0418829298986888e-25] + = 9.593018563556891e-27

T: 76

i: 1
β(76-1,1) = Σ[[trans(25,1) * emi(1,100) * β(100,1)][trans(25,2) * emi(2,100) * β(100,2)][trans(25,3) * emi(3,100) * β(100,3)]
β(75,1)[0.97 * 0.06409 * 1.3087358834766136e-27][0.0 * 0.06927 * 1.3087358834766136e-27][0.03 * 0.04995 * 1.3087358834766136e-27] + = 3.160301416826376e-29
i: 2
β(76-1,2) = Σ[[trans(25,1) * emi(1,99) * β(99,1)][trans(25,2) * emi(2,99) * β(99,2)][trans(25,3) * emi(3,99) * β(99,3)]
β(75,2)[0.0 * 0.06409 * 1.0907077058498902e-26][0.6 * 0.06927 * 1.0907077058498902e-26][0.4 * 0.04995 * 1.0907077058498902e-26] + = 2.646972013088668e-28
i: 3
β(76-1,3) = Σ[[trans(25,1) * emi(1,98) * β(98,1)][trans(25,2) * emi(2,98) * β(98,2)][trans(25,3) * emi(3,98) * β(98,3)]
β(75,3)[0.07 * 0.06409 * 9.593018563556891e-27][0.03 * 0.06927 * 9.593018563556891e-27][0.9 * 0.04995 * 9.593018563556891e-27] + = 3.8974863823969405e-28

T: 75

i: 1
β(75-1,1) = Σ[[trans(26,1) * emi(1,100) * β(100,1)][trans(26,2) * emi(2,100) * β(100,2)][trans(26,3) * emi(3,100) * β(100,3)]
β(74,1)[0.97 * 0.01482 * 3.160301416826376e-29][0.0 * 0.01439 * 3.160301416826376e-29][0.03 * 0.04444 * 3.160301416826376e-29] + = 2.021291175764511e-30
i: 2
β(75-1,2) = Σ[[trans(26,1) * emi(1,99) * β(99,1)][trans(26,2) * emi(2,99) * β(99,2)][trans(26,3) * emi(3,99) * β(99,3)]
β(74,2)[0.0 * 0.01482 * 2.646972013088668e-28][0.6 * 0.01439 * 2.646972013088668e-28][0.4 * 0.04444 * 2.646972013088668e-28] + = 9.712481245750562e-30
i: 3
β(75-1,3) = Σ[[trans(26,1) * emi(1,98) * β(98,1)][trans(26,2) * emi(2,98) * β(98,2)][trans(26,3) * emi(3,98) * β(98,3)]
β(74,3)[0.07 * 0.01482 * 3.8974863823969405e-28][0.03 * 0.01439 * 3.8974863823969405e-28][0.9 * 0.04444 * 3.8974863823969405e-28] + = 7.060207385295339e-30

T: 74

i: 1
β(74-1,1) = Σ[[trans(27,1) * emi(1,100) * β(100,1)][trans(27,2) * emi(2,100) * β(100,2)][trans(27,3) * emi(3,100) * β(100,3)]
β(73,1)[0.97 * 0.05877 * 2.021291175764511e-30][0.0 * 0.04271 * 2.021291175764511e-30][0.03 * 0.01879 * 2.021291175764511e-30] + = 1.7812518243105253e-31
i: 2
β(74-1,2) = Σ[[trans(27,1) * emi(1,99) * β(99,1)][trans(27,2) * emi(2,99) * β(99,2)][trans(27,3) * emi(3,99) * β(99,3)]
β(73,2)[0.0 * 0.05877 * 9.712481245750562e-30][0.6 * 0.04271 * 9.712481245750562e-30][0.4 * 0.01879 * 9.712481245750562e-30] + = 3.397936657386402e-31
i: 3
β(74-1,3) = Σ[[trans(27,1) * emi(1,98) * β(98,1)][trans(27,2) * emi(2,98) * β(98,2)][trans(27,3) * emi(3,98) * β(98,3)]
β(73,3)[0.07 * 0.05877 * 7.060207385295339e-30][0.03 * 0.04271 * 7.060207385295339e-30][0.9 * 0.01879 * 7.060207385295339e-30] + = 5.45109140010743e-32

T: 73

i: 1
β(73-1,1) = Σ[[trans(28,1) * emi(1,100) * β(100,1)][trans(28,2) * emi(2,100) * β(100,2)][trans(28,3) * emi(3,100) * β(100,3)]
β(72,1)[0.97 * 0.09042 * 1.7812518243105253e-31][0.0 * 0.05638 * 1.7812518243105253e-31][0.03 * 0.00398 * 1.7812518243105253e-31] + = 7.991703186044316e-33
i: 2
β(73-1,2) = Σ[[trans(28,1) * emi(1,99) * β(99,1)][trans(28,2) * emi(2,99) * β(99,2)][trans(28,3) * emi(3,99) * β(99,3)]
β(72,2)[0.0 * 0.09042 * 3.397936657386402e-31][0.6 * 0.05638 * 3.397936657386402e-31][0.4 * 0.00398 * 3.397936657386402e-31] + = 1.1127309578675791e-32
i: 3
β(73-1,3) = Σ[[trans(28,1) * emi(1,98) * β(98,1)][trans(28,2) * emi(2,98) * β(98,2)][trans(28,3) * emi(3,98) * β(98,3)]
β(72,3)[0.07 * 0.09042 * 5.45109140010743e-32][0.03 * 0.05638 * 5.45109140010743e-32][0.9 * 0.00398 * 5.45109140010743e-32] + = 1.3517680982902358e-33

T: 72

i: 1
β(72-1,1) = Σ[[trans(29,1) * emi(1,100) * β(100,1)][trans(29,2) * emi(2,100) * β(100,2)][trans(29,3) * emi(3,100) * β(100,3)]
β(71,1)[0.97 * 0.04621 * 7.991703186044316e-33][0.0 * 0.05409 * 7.991703186044316e-33][0.03 * 0.00457 * 7.991703186044316e-33] + = 3.950079998514329e-34
i: 2

β(72-1,2) = Σ[[trans(29,1) * emi(1,99) * β(99,1)][trans(29,2) * emi(2,99) * β(99,2)][trans(29,3) * emi(3,99) * β(99,3)]
β(71,2)[0.0 * 0.04621 * 1.1127309578675791e-32][0.6 * 0.05409 * 1.1127309578675791e-32][0.4 * 0.00457 * 1.1127309578675791e-32] + = 2.682330620383825e-34
i: 3
β(72-1,3) = Σ[[trans(29,1) * emi(1,98) * β(98,1)][trans(29,2) * emi(2,98) * β(98,2)][trans(29,3) * emi(3,98) * β(98,3)]
β(71,3)[0.07 * 0.04621 * 1.3517680982902358e-33][0.03 * 0.05409 * 1.3517680982902358e-33][0.9 * 0.00457 * 1.3517680982902358e-33] + = 5.006952915327894e-35

T: 71

i: 1
β(71-1,1) = Σ[[trans(30,1) * emi(1,100) * β(100,1)][trans(30,2) * emi(2,100) * β(100,2)][trans(30,3) * emi(3,100) * β(100,3)]
β(70,1)[0.97 * 0.05092 * 3.950079998514329e-34][0.0 * 0.03962 * 3.950079998514329e-34][0.03 * 0.00687 * 3.950079998514329e-34] + = 2.0104928026723787e-35
i: 2
β(71-1,2) = Σ[[trans(30,1) * emi(1,99) * β(99,1)][trans(30,2) * emi(2,99) * β(99,2)][trans(30,3) * emi(3,99) * β(99,3)]
β(70,2)[0.0 * 0.05092 * 2.682330620383825e-34][0.6 * 0.03962 * 2.682330620383825e-34][0.4 * 0.00687 * 2.682330620383825e-34] + = 2.0516288712815173e-35
i: 3
β(71-1,3) = Σ[[trans(30,1) * emi(1,98) * β(98,1)][trans(30,2) * emi(2,98) * β(98,2)][trans(30,3) * emi(3,98) * β(98,3)]
β(70,3)[0.07 * 0.05092 * 5.006952915327894e-35][0.03 * 0.03962 * 5.006952915327894e-35][0.9 * 0.00687 * 5.006952915327894e-35] + = 1.1016272653590688e-35

T: 70

i: 1
β(70-1,1) = Σ[[trans(31,1) * emi(1,100) * β(100,1)][trans(31,2) * emi(2,100) * β(100,2)][trans(31,3) * emi(3,100) * β(100,3)]
β(69,1)[0.97 * 0.05171 * 2.0104928026723787e-35][0.0 * 0.1033 * 2.0104928026723787e-35][0.03 * 0.19429 * 2.0104928026723787e-35] + = 4.4195952515335675e-37
i: 2
β(70-1,2) = Σ[[trans(31,1) * emi(1,99) * β(99,1)][trans(31,2) * emi(2,99) * β(99,2)][trans(31,3) * emi(3,99) * β(99,3)]
β(69,2)[0.0 * 0.05171 * 2.0516288712815173e-35][0.6 * 0.1033 * 2.0516288712815173e-35][0.4 * 0.19429 * 2.0516288712815173e-35] + = 2.965339728779327e-37
i: 3
β(70-1,3) = Σ[[trans(31,1) * emi(1,98) * β(98,1)][trans(31,2) * emi(2,98) * β(98,2)][trans(31,3) * emi(3,98) * β(98,3)]
β(69,3)[0.07 * 0.05171 * 1.1016272653590688e-35][0.03 * 0.1033 * 1.1016272653590688e-35][0.9 * 0.19429 * 1.1016272653590688e-35] + = 1.223437625277924e-37

T: 69

i: 1
β(69-1,1) = Σ[[trans(32,1) * emi(1,100) * β(100,1)][trans(32,2) * emi(2,100) * β(100,2)][trans(32,3) * emi(3,100) * β(100,3)]
β(68,1)[0.97 * 0.02253 * 4.4195952515335675e-37][0.0 * 0.02129 * 4.4195952515335675e-37][0.03 * 0.00782 * 4.4195952515335675e-37] + = 2.2881220322955418e-38
i: 2
β(69-1,2) = Σ[[trans(32,1) * emi(1,99) * β(99,1)][trans(32,2) * emi(2,99) * β(99,2)][trans(32,3) * emi(3,99) * β(99,3)]
β(68,2)[0.0 * 0.02253 * 2.965339728779327e-37][0.6 * 0.02129 * 2.965339728779327e-37][0.4 * 0.00782 * 2.965339728779327e-37] + = 2.7887243487584186e-38
i: 3
β(69-1,3) = Σ[[trans(32,1) * emi(1,98) * β(98,1)][trans(32,2) * emi(2,98) * β(98,2)][trans(32,3) * emi(3,98) * β(98,3)]
β(68,3)[0.07 * 0.02253 * 1.223437625277924e-37][0.03 * 0.02129 * 1.223437625277924e-37][0.9 * 0.00782 * 1.223437625277924e-37] + = 2.3911872334518627e-38

T: 68

i: 1
β(68-1,1) = Σ[[trans(33,1) * emi(1,100) * β(100,1)][trans(33,2) * emi(2,100) * β(100,2)][trans(33,3) * emi(3,100) * β(100,3)]
β(67,1)[0.97 * 0.05171 * 2.2881220322955418e-38][0.0 * 0.1033 * 2.2881220322955418e-38][0.03 * 0.19429 * 2.2881220322955418e-38] + = 1.0386604715414183e-39
i: 2
β(68-1,2) = Σ[[trans(33,1) * emi(1,99) * β(99,1)][trans(33,2) * emi(2,99) * β(99,2)][trans(33,3) * emi(3,99) * β(99,3)]
β(67,2)[0.0 * 0.05171 * 2.7887243487584186e-38][0.6 * 0.1033 * 2.7887243487584186e-38][0.4 * 0.19429 * 2.7887243487584186e-38] + = 1.8538487584097945e-39
i: 3
β(68-1,3) = Σ[[trans(33,1) * emi(1,98) * β(98,1)][trans(33,2) * emi(2,98) * β(98,2)][trans(33,3) * emi(3,98) * β(98,3)]
β(67,3)[0.07 * 0.05171 * 2.3911872334518627e-38][0.03 * 0.1033 * 2.3911872334518627e-38][0.9 * 0.19429 * 2.3911872334518627e-38] + = 2.6728849375170516e-39

T: 67

i: 1
β(67-1,1) = Σ[[trans(34,1) * emi(1,100) * β(100,1)][trans(34,2) * emi(2,100) * β(100,2)][trans(34,3) * emi(3,100) * β(100,3)]
β(66,1)[0.97 * 0.04294 * 1.0386604715414183e-39][0.0 * 0.04257 * 1.0386604715414183e-39][0.03 * 0.11935 * 1.0386604715414183e-39] + = 6.857603521132611e-41
i: 2
β(67-1,2) = Σ[[trans(34,1) * emi(1,99) * β(99,1)][trans(34,2) * emi(2,99) * β(99,2)][trans(34,3) * emi(3,99) * β(99,3)]
β(66,2)[0.0 * 0.04294 * 1.8538487584097945e-39][0.6 * 0.04257 * 1.8538487584097945e-39][0.4 * 0.11935 * 1.8538487584097945e-39] + = 1.3045390314861857e-40
i: 3
β(67-1,3) = Σ[[trans(34,1) * emi(1,98) * β(98,1)][trans(34,2) * emi(2,98) * β(98,2)][trans(34,3) * emi(3,98) * β(98,3)]
β(66,3)[0.07 * 0.04294 * 2.6728849375170516e-39][0.03 * 0.04257 * 2.6728849375170516e-39][0.9 * 0.11935 * 2.6728849375170516e-39] + = 1.286717679444067e-40

T: 66

i: 1
β(66-1,1) = Σ[[trans(35,1) * emi(1,100) * β(100,1)][trans(35,2) * emi(2,100) * β(100,2)][trans(35,3) * emi(3,100) * β(100,3)]
β(65,1)[0.97 * 0.06409 * 6.857603521132611e-41][0.0 * 0.06927 * 6.857603521132611e-41][0.03 * 0.04995 * 6.857603521132611e-41] + = 4.1896739111719064e-42
i: 2
β(66-1,2) = Σ[[trans(35,1) * emi(1,99) * β(99,1)][trans(35,2) * emi(2,99) * β(99,2)][trans(35,3) * emi(3,99) * β(99,3)]
β(65,2)[0.0 * 0.06409 * 1.3045390314861857e-40][0.6 * 0.06927 * 1.3045390314861857e-40][0.4 * 0.04995 * 1.3045390314861857e-40] + = 1.808538803471889e-41
i: 3
β(66-1,3) = Σ[[trans(35,1) * emi(1,98) * β(98,1)][trans(35,2) * emi(2,98) * β(98,2)][trans(35,3) * emi(3,98) * β(98,3)]
β(65,3)[0.07 * 0.06409 * 1.286717679444067e-40][0.03 * 0.06927 * 1.286717679444067e-40][0.9 * 0.04995 * 1.286717679444067e-40] + = 2.3152175335038903e-41

T: 65

i: 1
β(65-1,1) = Σ[[trans(36,1) * emi(1,100) * β(100,1)][trans(36,2) * emi(2,100) * β(100,2)][trans(36,3) * emi(3,100) * β(100,3)]
β(64,1)[0.97 * 0.05171 * 4.1896739111719064e-42][0.0 * 0.1033 * 4.1896739111719064e-42][0.03 * 0.19429 * 4.1896739111719064e-42] + = 9.127565743204347e-44

i: 2
β(65-1,2) = Σ[[trans(36,1) * emi(1,99) * β(99,1)][trans(36,2) * emi(2,99) * β(99,2)][trans(36,3) * emi(3,99) * β(99,3)]
β(64,2)[0.0 * 0.05171 * 1.808538803471889e-41][0.6 * 0.1033 * 1.808538803471889e-41][0.4 * 0.19429 * 1.808538803471889e-41] + = 2.962785435284031e-43
i: 3
β(65-1,3) = Σ[[trans(36,1) * emi(1,98) * β(98,1)][trans(36,2) * emi(2,98) * β(98,2)][trans(36,3) * emi(3,98) * β(98,3)]
β(64,3)[0.07 * 0.05171 * 2.3152175335038903e-41][0.03 * 0.1033 * 2.3152175335038903e-41][0.9 * 0.19429 * 2.3152175335038903e-41] + = 2.913007751504781e-43

T: 64

i: 1
β(64-1,1) = Σ[[trans(37,1) * emi(1,100) * β(100,1)][trans(37,2) * emi(2,100) * β(100,2)][trans(37,3) * emi(3,100) * β(100,3)]
β(63,1)[0.97 * 0.02019 * 9.127565743204347e-44][0.0 * 0.01597 * 9.127565743204347e-44][0.03 * 0.01328 * 9.127565743204347e-44] + = 5.96980151480564e-45
i: 2
β(64-1,2) = Σ[[trans(37,1) * emi(1,99) * β(99,1)][trans(37,2) * emi(2,99) * β(99,2)][trans(37,3) * emi(3,99) * β(99,3)]
β(63,2)[0.0 * 0.02019 * 2.962785435284031e-43][0.6 * 0.01597 * 2.962785435284031e-43][0.4 * 0.01328 * 2.962785435284031e-43] + = 1.254711782884985e-44
i: 3
β(64-1,3) = Σ[[trans(37,1) * emi(1,98) * β(98,1)][trans(37,2) * emi(2,98) * β(98,2)][trans(37,3) * emi(3,98) * β(98,3)]
β(63,3)[0.07 * 0.02019 * 2.913007751504781e-43][0.03 * 0.01597 * 2.913007751504781e-43][0.9 * 0.01328 * 2.913007751504781e-43] + = 6.801503445616711e-45

T: 63

i: 1
β(63-1,1) = Σ[[trans(38,1) * emi(1,100) * β(100,1)][trans(38,2) * emi(2,100) * β(100,2)][trans(38,3) * emi(3,100) * β(100,3)]
β(62,1)[0.97 * 0.06521 * 5.96980151480564e-45][0.0 * 0.05586 * 5.96980151480564e-45][0.03 * 0.02246 * 5.96980151480564e-45] + = 2.3208364852330647e-46
i: 2
β(63-1,2) = Σ[[trans(38,1) * emi(1,99) * β(99,1)][trans(38,2) * emi(2,99) * β(99,2)][trans(38,3) * emi(3,99) * β(99,3)]
β(62,2)[0.0 * 0.06521 * 1.254711782884985e-44][0.6 * 0.05586 * 1.254711782884985e-44][0.4 * 0.02246 * 1.254711782884985e-44] + = 4.841186879774464e-46
i: 3
β(63-1,3) = Σ[[trans(38,1) * emi(1,98) * β(98,1)][trans(38,2) * emi(2,98) * β(98,2)][trans(38,3) * emi(3,98) * β(98,3)]
β(62,3)[0.07 * 0.06521 * 6.801503445616711e-45][0.03 * 0.05586 * 6.801503445616711e-45][0.9 * 0.02246 * 6.801503445616711e-45] + = 1.3391645178095297e-46

T: 62

i: 1
β(62-1,1) = Σ[[trans(39,1) * emi(1,100) * β(100,1)][trans(39,2) * emi(2,100) * β(100,2)][trans(39,3) * emi(3,100) * β(100,3)]
β(61,1)[0.97 * 0.03953 * 2.3208364852330647e-46][0.0 * 0.05868 * 2.3208364852330647e-46][0.03 * 0.01557 * 2.3208364852330647e-46] + = 8.9615910019694e-48
i: 2
β(62-1,2) = Σ[[trans(39,1) * emi(1,99) * β(99,1)][trans(39,2) * emi(2,99) * β(99,2)][trans(39,3) * emi(3,99) * β(99,3)]
β(61,2)[0.0 * 0.03953 * 4.841186879774464e-46][0.6 * 0.05868 * 4.841186879774464e-46][0.4 * 0.01557 * 4.841186879774464e-46] + = 1.7878882428001713e-47
i: 3
β(62-1,3) = Σ[[trans(39,1) * emi(1,98) * β(98,1)][trans(39,2) * emi(2,98) * β(98,2)][trans(39,3) * emi(3,98) * β(98,3)]
β(61,3)[0.07 * 0.03953 * 1.3391645178095297e-46][0.03 * 0.05868 * 1.3391645178095297e-46][0.9 * 0.01557 * 1.3391645178095297e-46] + = 3.3710124409508326e-48

T: 61

i: 1
β(61-1,1) = Σ[[trans(40,1) * emi(1,100) * β(100,1)][trans(40,2) * emi(2,100) * β(100,2)][trans(40,3) * emi(3,100) * β(100,3)]
β(60,1)[0.97 * 0.03953 * 8.9615910019694e-48][0.0 * 0.05868 * 8.9615910019694e-48][0.03 * 0.01557 * 8.9615910019694e-48] + = 4.691503748050527e-49
i: 2
β(61-1,2) = Σ[[trans(40,1) * emi(1,99) * β(99,1)][trans(40,2) * emi(2,99) * β(99,2)][trans(40,3) * emi(3,99) * β(99,3)]
β(60,2)[0.0 * 0.03953 * 1.7878882428001713e-47][0.6 * 0.05868 * 1.7878882428001713e-47][0.4 * 0.01557 * 1.7878882428001713e-47] + = 1.370114735748481e-48
i: 3
β(61-1,3) = Σ[[trans(40,1) * emi(1,98) * β(98,1)][trans(40,2) * emi(2,98) * β(98,2)][trans(40,3) * emi(3,98) * β(98,3)]
β(60,3)[0.07 * 0.03953 * 3.3710124409508326e-48][0.03 * 0.05868 * 3.3710124409508326e-48][0.9 * 0.01557 * 3.3710124409508326e-48] + = 6.7730353403130944e-49

T: 60

i: 1
β(60-1,1) = Σ[[trans(41,1) * emi(1,100) * β(100,1)][trans(41,2) * emi(2,100) * β(100,2)][trans(41,3) * emi(3,100) * β(100,3)]
β(59,1)[0.97 * 0.05171 * 4.691503748050527e-49][0.0 * 0.1033 * 4.691503748050527e-49][0.03 * 0.19429 * 4.691503748050527e-49] + = 1.6328373187796943e-50
i: 2
β(60-1,2) = Σ[[trans(41,1) * emi(1,99) * β(99,1)][trans(41,2) * emi(2,99) * β(99,2)][trans(41,3) * emi(3,99) * β(99,3)]
β(59,2)[0.0 * 0.05171 * 1.370114735748481e-48][0.6 * 0.1033 * 1.370114735748481e-48][0.4 * 0.19429 * 1.370114735748481e-48] + = 3.917696642621639e-50
i: 3
β(60-1,3) = Σ[[trans(41,1) * emi(1,98) * β(98,1)][trans(41,2) * emi(2,98) * β(98,2)][trans(41,3) * emi(3,98) * β(98,3)]
β(59,3)[0.07 * 0.05171 * 6.7730353403130944e-49][0.03 * 0.1033 * 6.7730353403130944e-49][0.9 * 0.19429 * 6.7730353403130944e-49] + = 4.202740532773043e-50

T: 59

i: 1
β(59-1,1) = Σ[[trans(42,1) * emi(1,100) * β(100,1)][trans(42,2) * emi(2,100) * β(100,2)][trans(42,3) * emi(3,100) * β(100,3)]
β(58,1)[0.97 * 0.03296 * 1.6328373187796943e-50][0.0 * 0.0261 * 1.6328373187796943e-50][0.03 * 0.06541 * 1.6328373187796943e-50] + = 9.725032771670406e-52
i: 2
β(59-1,2) = Σ[[trans(42,1) * emi(1,99) * β(99,1)][trans(42,2) * emi(2,99) * β(99,2)][trans(42,3) * emi(3,99) * β(99,3)]
β(58,2)[0.0 * 0.03296 * 3.917696642621639e-50][0.6 * 0.0261 * 3.917696642621639e-50][0.4 * 0.06541 * 3.917696642621639e-50] + = 2.86123252755902e-51
i: 3
β(59-1,3) = Σ[[trans(42,1) * emi(1,98) * β(98,1)][trans(42,2) * emi(2,98) * β(98,2)][trans(42,3) * emi(3,98) * β(98,3)]
β(58,3)[0.07 * 0.03296 * 4.202740532773043e-50][0.03 * 0.0261 * 4.202740532773043e-50][0.9 * 0.06541 * 4.202740532773043e-50] + = 2.7581303496890555e-51

T: 58

i: 1
β(58-1,1) = Σ[[trans(43,1) * emi(1,100) * β(100,1)][trans(43,2) * emi(2,100) * β(100,2)][trans(43,3) * emi(3,100) * β(100,3)]

β(57,1)[0.97 * 0.05591 * 9.725032771670406e-52][0.0 * 0.07239 * 9.725032771670406e-52][0.03 * 0.06898 * 9.725032771670406e-52] + = 4.396933481569466e-53
i: 2
β(58-1,2) = Σ[[trans(43,1) * emi(1,99) * β(99,1)][trans(43,2) * emi(2,99) * β(99,2)][trans(43,3) * emi(3,99) * β(99,3)]
β(57,2)[0.0 * 0.05591 * 2.86123252755902e-51][0.6 * 0.07239 * 2.86123252755902e-51][0.4 * 0.06898 * 2.86123252755902e-51] + = 9.790030272863204e-53
i: 3
β(58-1,3) = Σ[[trans(43,1) * emi(1,98) * β(98,1)][trans(43,2) * emi(2,98) * β(98,2)][trans(43,3) * emi(3,98) * β(98,3)]
β(57,3)[0.07 * 0.05591 * 2.7581303496890555e-51][0.03 * 0.07239 * 2.7581303496890555e-51][0.9 * 0.06898 * 2.7581303496890555e-51] + = 1.9132868501393335e-53

T: 57

i: 1
β(57-1,1) = Σ[[trans(44,1) * emi(1,100) * β(100,1)][trans(44,2) * emi(2,100) * β(100,2)][trans(44,3) * emi(3,100) * β(100,3)]
β(56,1)[0.97 * 0.04621 * 4.396933481569466e-53][0.0 * 0.05409 * 4.396933481569466e-53][0.03 * 0.00457 * 4.396933481569466e-53] + = 1.3337218976427874e-54
i: 2
β(57-1,2) = Σ[[trans(44,1) * emi(1,99) * β(99,1)][trans(44,2) * emi(2,99) * β(99,2)][trans(44,3) * emi(3,99) * β(99,3)]
β(56,2)[0.0 * 0.04621 * 9.790030272863204e-53][0.6 * 0.05409 * 9.790030272863204e-53][0.4 * 0.00457 * 9.790030272863204e-53] + = 3.97604985410847e-54
i: 3
β(57-1,3) = Σ[[trans(44,1) * emi(1,98) * β(98,1)][trans(44,2) * emi(2,98) * β(98,2)][trans(44,3) * emi(3,98) * β(98,3)]
β(56,3)[0.07 * 0.04621 * 1.9132868501393335e-53][0.03 * 0.05409 * 1.9132868501393335e-53][0.9 * 0.00457 * 1.9132868501393335e-53] + = 1.831503043202433e-54

T: 56

i: 1
β(56-1,1) = Σ[[trans(45,1) * emi(1,100) * β(100,1)][trans(45,2) * emi(2,100) * β(100,2)][trans(45,3) * emi(3,100) * β(100,3)]
β(55,1)[0.97 * 0.03004 * 1.3337218976427874e-54][0.0 * 0.05577 * 1.3337218976427874e-54][0.03 * 0.09148 * 1.3337218976427874e-54] + = 8.56583966375673e-56
i: 2
β(56-1,2) = Σ[[trans(45,1) * emi(1,99) * β(99,1)][trans(45,2) * emi(2,99) * β(99,2)][trans(45,3) * emi(3,99) * β(99,3)]
β(55,2)[0.0 * 0.03004 * 3.97604985410847e-54][0.6 * 0.05577 * 3.97604985410847e-54][0.4 * 0.09148 * 3.97604985410847e-54] + = 2.0184601483964083e-55
i: 3
β(56-1,3) = Σ[[trans(45,1) * emi(1,98) * β(98,1)][trans(45,2) * emi(2,98) * β(98,2)][trans(45,3) * emi(3,98) * β(98,3)]
β(55,3)[0.07 * 0.03004 * 1.831503043202433e-54][0.03 * 0.05577 * 1.831503043202433e-54][0.9 * 0.09148 * 1.831503043202433e-54] + = 9.658132505838303e-56

T: 55

i: 1
β(55-1,1) = Σ[[trans(46,1) * emi(1,100) * β(100,1)][trans(46,2) * emi(2,100) * β(100,2)][trans(46,3) * emi(3,100) * β(100,3)]
β(54,1)[0.97 * 0.06409 * 8.56583966375673e-56][0.0 * 0.06927 * 8.56583966375673e-56][0.03 * 0.04995 * 8.56583966375673e-56] + = 4.8594573887925444e-57
i: 2
β(55-1,2) = Σ[[trans(46,1) * emi(1,99) * β(99,1)][trans(46,2) * emi(2,99) * β(99,2)][trans(46,3) * emi(3,99) * β(99,3)]
β(54,2)[0.0 * 0.06409 * 2.0184601483964083e-55][0.6 * 0.06927 * 2.0184601483964083e-55][0.4 * 0.04995 * 2.0184601483964083e-55] + = 2.0016330257998236e-56
i: 3
β(55-1,3) = Σ[[trans(46,1) * emi(1,98) * β(98,1)][trans(46,2) * emi(2,98) * β(98,2)][trans(46,3) * emi(3,98) * β(98,3)]
β(54,3)[0.07 * 0.06409 * 9.658132505838303e-56][0.03 * 0.06927 * 9.658132505838303e-56][0.9 * 0.04995 * 9.658132505838303e-56] + = 1.7823885579330963e-56

T: 54

i: 1
β(54-1,1) = Σ[[trans(47,1) * emi(1,100) * β(100,1)][trans(47,2) * emi(2,100) * β(100,2)][trans(47,3) * emi(3,100) * β(100,3)]
β(53,1)[0.97 * 0.05171 * 4.8594573887925444e-57][0.0 * 0.1033 * 4.8594573887925444e-57][0.03 * 0.19429 * 4.8594573887925444e-57] + = 2.8706992571822657e-58
i: 2
β(54-1,2) = Σ[[trans(47,1) * emi(1,99) * β(99,1)][trans(47,2) * emi(2,99) * β(99,2)][trans(47,3) * emi(3,99) * β(99,3)]
β(53,2)[0.0 * 0.05171 * 2.0016330257998236e-56][0.6 * 0.1033 * 2.0016330257998236e-56][0.4 * 0.19429 * 2.0016330257998236e-56] + = 6.469028032057142e-58
i: 3
β(54-1,3) = Σ[[trans(47,1) * emi(1,98) * β(98,1)][trans(47,2) * emi(2,98) * β(98,2)][trans(47,3) * emi(3,98) * β(98,3)]
β(53,3)[0.07 * 0.05171 * 1.7823885579330963e-56][0.03 * 0.1033 * 1.7823885579330963e-56][0.9 * 0.19429 * 1.7823885579330963e-56] + = 3.4705797474339275e-58

T: 53

i: 1
β(53-1,1) = Σ[[trans(48,1) * emi(1,100) * β(100,1)][trans(48,2) * emi(2,100) * β(100,2)][trans(48,3) * emi(3,100) * β(100,3)]
β(52,1)[0.97 * 0.05877 * 2.8706992571822657e-58][0.0 * 0.04271 * 2.8706992571822657e-58][0.03 * 0.01879 * 2.8706992571822657e-58] + = 1.8392082623785085e-59
i: 2
β(53-1,2) = Σ[[trans(48,1) * emi(1,99) * β(99,1)][trans(48,2) * emi(2,99) * β(99,2)][trans(48,3) * emi(3,99) * β(99,3)]
β(52,2)[0.0 * 0.05877 * 6.469028032057142e-58][0.6 * 0.04271 * 6.469028032057142e-58][0.4 * 0.01879 * 6.469028032057142e-58] + = 2.479956319733736e-59
i: 3
β(53-1,3) = Σ[[trans(48,1) * emi(1,98) * β(98,1)][trans(48,2) * emi(2,98) * β(98,2)][trans(48,3) * emi(3,98) * β(98,3)]
β(52,3)[0.07 * 0.05877 * 3.4705797474339275e-58][0.03 * 0.04271 * 3.4705797474339275e-58][0.9 * 0.01879 * 3.4705797474339275e-58] + = 9.409897709001067e-60

T: 52

i: 1
β(52-1,1) = Σ[[trans(49,1) * emi(1,100) * β(100,1)][trans(49,2) * emi(2,100) * β(100,2)][trans(49,3) * emi(3,100) * β(100,3)]
β(51,1)[0.97 * 0.06521 * 1.8392082623785085e-59][0.0 * 0.05586 * 1.8392082623785085e-59][0.03 * 0.02246 * 1.8392082623785085e-59] + = 2.0572532441637598e-60
i: 2
β(52-1,2) = Σ[[trans(49,1) * emi(1,99) * β(99,1)][trans(49,2) * emi(2,99) * β(99,2)][trans(49,3) * emi(3,99) * β(99,3)]
β(51,2)[0.0 * 0.06521 * 2.479956319733736e-59][0.6 * 0.05586 * 2.479956319733736e-59][0.4 * 0.02246 * 2.479956319733736e-59] + = 1.108171577651138e-60
i: 3
β(52-1,3) = Σ[[trans(49,1) * emi(1,98) * β(98,1)][trans(49,2) * emi(2,98) * β(98,2)][trans(49,3) * emi(3,98) * β(98,3)]
β(51,3)[0.07 * 0.06521 * 9.409897709001067e-60][0.03 * 0.05586 * 9.409897709001067e-60][0.9 * 0.02246 * 9.409897709001067e-60] + = 2.7424823330809296e-61

T: 51

i: 1

β(51-1,1) = Σ[[trans(50,1) * emi(1,100) * β(100,1)][trans(50,2) * emi(2,100) * β(100,2)][trans(50,3) * emi(3,100) * β(100,3)]
β(50,1)[0.97 * 0.11518 * 2.0572532441637598e-60][0.0 * 0.07232 * 2.0572532441637598e-60][0.03 * 0.00852 * 2.0572532441637598e-60] + = 1.174322236938348e-61
i: 2
β(51-1,2) = Σ[[trans(50,1) * emi(1,99) * β(99,1)][trans(50,2) * emi(2,99) * β(99,2)][trans(50,3) * emi(3,99) * β(99,3)]
β(50,2)[0.0 * 0.11518 * 1.108171577651138e-60][0.6 * 0.07232 * 1.108171577651138e-60][0.4 * 0.00852 * 1.108171577651138e-60] + = 3.045925457043169e-62
i: 3
β(51-1,3) = Σ[[trans(50,1) * emi(1,98) * β(98,1)][trans(50,2) * emi(2,98) * β(98,2)][trans(50,3) * emi(3,98) * β(98,3)]
β(50,3)[0.07 * 0.11518 * 2.7424823330809296e-61][0.03 * 0.07232 * 2.7424823330809296e-61][0.9 * 0.00852 * 2.7424823330809296e-61] + = 1.4521046237082855e-62

T: 50

i: 1
β(50-1,1) = Σ[[trans(51,1) * emi(1,100) * β(100,1)][trans(51,2) * emi(2,100) * β(100,2)][trans(51,3) * emi(3,100) * β(100,3)]
β(49,1)[0.97 * 0.05877 * 1.174322236938348e-61][0.0 * 0.04271 * 1.174322236938348e-61][0.03 * 0.01879 * 1.174322236938348e-61] + = 5.265737600624446e-63
i: 2
β(50-1,2) = Σ[[trans(51,1) * emi(1,99) * β(99,1)][trans(51,2) * emi(2,99) * β(99,2)][trans(51,3) * emi(3,99) * β(99,3)]
β(49,2)[0.0 * 0.05877 * 3.045925457043169e-62][0.6 * 0.04271 * 3.045925457043169e-62][0.4 * 0.01879 * 3.045925457043169e-62] + = 1.0150691203501774e-63
i: 3
β(50-1,3) = Σ[[trans(51,1) * emi(1,98) * β(98,1)][trans(51,2) * emi(2,98) * β(98,2)][trans(51,3) * emi(3,98) * β(98,3)]
β(49,3)[0.07 * 0.05877 * 1.4521046237082855e-62][0.03 * 0.04271 * 1.4521046237082855e-62][0.9 * 0.01879 * 1.4521046237082855e-62] + = 4.890093095470087e-64

T: 49

i: 1
β(49-1,1) = Σ[[trans(52,1) * emi(1,100) * β(100,1)][trans(52,2) * emi(2,100) * β(100,2)][trans(52,3) * emi(3,100) * β(100,3)]
β(48,1)[0.97 * 0.04621 * 5.265737600624446e-63][0.0 * 0.05409 * 5.265737600624446e-63][0.03 * 0.00457 * 5.265737600624446e-63] + = 2.86587123438562e-64
i: 2
β(49-1,2) = Σ[[trans(52,1) * emi(1,99) * β(99,1)][trans(52,2) * emi(2,99) * β(99,2)][trans(52,3) * emi(3,99) * β(99,3)]
β(48,2)[0.0 * 0.04621 * 1.0150691203501774e-63][0.6 * 0.05409 * 1.0150691203501774e-63][0.4 * 0.00457 * 1.0150691203501774e-63] + = 5.7581257042310665e-65
i: 3
β(49-1,3) = Σ[[trans(52,1) * emi(1,98) * β(98,1)][trans(52,2) * emi(2,98) * β(98,2)][trans(52,3) * emi(3,98) * β(98,3)]
β(48,3)[0.07 * 0.04621 * 4.890093095470087e-64][0.03 * 0.05409 * 4.890093095470087e-64][0.9 * 0.00457 * 4.890093095470087e-64] + = 5.3171618811525766e-65

T: 48

i: 1
β(48-1,1) = Σ[[trans(53,1) * emi(1,100) * β(100,1)][trans(53,2) * emi(2,100) * β(100,2)][trans(53,3) * emi(3,100) * β(100,3)]
β(47,1)[0.97 * 0.05591 * 2.86587123438562e-64][0.0 * 0.07239 * 2.86587123438562e-64][0.03 * 0.06898 * 2.86587123438562e-64] + = 1.1518158186390305e-65
i: 2
β(48-1,2) = Σ[[trans(53,1) * emi(1,99) * β(99,1)][trans(53,2) * emi(2,99) * β(99,2)][trans(53,3) * emi(3,99) * β(99,3)]
β(47,2)[0.0 * 0.05591 * 5.7581257042310665e-65][0.6 * 0.07239 * 5.7581257042310665e-65][0.4 * 0.06898 * 5.7581257042310665e-65] + = 1.39253260095916e-66
i: 3
β(48-1,3) = Σ[[trans(53,1) * emi(1,98) * β(98,1)][trans(53,2) * emi(2,98) * β(98,2)][trans(53,3) * emi(3,98) * β(98,3)]
β(47,3)[0.07 * 0.05591 * 5.3171618811525766e-65][0.03 * 0.07239 * 5.3171618811525766e-65][0.9 * 0.06898 * 5.3171618811525766e-65] + = 9.316400229378659e-67

T: 47

i: 1
β(47-1,1) = Σ[[trans(54,1) * emi(1,100) * β(100,1)][trans(54,2) * emi(2,100) * β(100,2)][trans(54,3) * emi(3,100) * β(100,3)]
β(46,1)[0.97 * 0.04143 * 1.1518158186390305e-65][0.0 * 0.0399 * 1.1518158186390305e-65][0.03 * 0.00066 * 1.1518158186390305e-65] + = 4.8308775825002065e-67
i: 2
β(47-1,2) = Σ[[trans(54,1) * emi(1,99) * β(99,1)][trans(54,2) * emi(2,99) * β(99,2)][trans(54,3) * emi(3,99) * β(99,3)]
β(46,2)[0.0 * 0.04143 * 1.39253260095916e-66][0.6 * 0.0399 * 1.39253260095916e-66][0.4 * 0.00066 * 1.39253260095916e-66] + = 8.00445623887526e-68
i: 3
β(47-1,3) = Σ[[trans(54,1) * emi(1,98) * β(98,1)][trans(54,2) * emi(2,98) * β(98,2)][trans(54,3) * emi(3,98) * β(98,3)]
β(46,3)[0.07 * 0.04143 * 9.316400229378659e-67][0.03 * 0.0399 * 9.316400229378659e-67][0.9 * 0.00066 * 9.316400229378659e-67] + = 1.364717963252078e-67

T: 46

i: 1
β(46-1,1) = Σ[[trans(55,1) * emi(1,100) * β(100,1)][trans(55,2) * emi(2,100) * β(100,2)][trans(55,3) * emi(3,100) * β(100,3)]
β(45,1)[0.97 * 0.04294 * 4.8308775825002065e-67][0.0 * 0.04257 * 4.8308775825002065e-67][0.03 * 0.11935 * 4.8308775825002065e-67] + = 3.0649042830383254e-68
i: 2
β(46-1,2) = Σ[[trans(55,1) * emi(1,99) * β(99,1)][trans(55,2) * emi(2,99) * β(99,2)][trans(55,3) * emi(3,99) * β(99,3)]
β(45,2)[0.0 * 0.04294 * 8.00445623887526e-68][0.6 * 0.04257 * 8.00445623887526e-68][0.4 * 0.11935 * 8.00445623887526e-68] + = 3.9088361712070983e-69
i: 3
β(46-1,3) = Σ[[trans(55,1) * emi(1,98) * β(98,1)][trans(55,2) * emi(2,98) * β(98,2)][trans(55,3) * emi(3,98) * β(98,3)]
β(45,3)[0.07 * 0.04294 * 1.364717963252078e-67][0.03 * 0.04257 * 1.364717963252078e-67][0.9 * 0.11935 * 1.364717963252078e-67] + = 5.097930258652691e-69

T: 45

i: 1
β(45-1,1) = Σ[[trans(56,1) * emi(1,100) * β(100,1)][trans(56,2) * emi(2,100) * β(100,2)][trans(56,3) * emi(3,100) * β(100,3)]
β(44,1)[0.97 * 0.06521 * 3.0649042830383254e-68][0.0 * 0.05586 * 3.0649042830383254e-68][0.03 * 0.02246 * 3.0649042830383254e-68] + = 4.4738881092472716e-70
i: 2
β(45-1,2) = Σ[[trans(56,1) * emi(1,99) * β(99,1)][trans(56,2) * emi(2,99) * β(99,2)][trans(56,3) * emi(3,99) * β(99,3)]
β(44,2)[0.0 * 0.06521 * 3.9088361712070983e-69][0.6 * 0.05586 * 3.9088361712070983e-69][0.4 * 0.02246 * 3.9088361712070983e-69] + = 1.2436969978001232e-70
i: 3
β(45-1,3) = Σ[[trans(56,1) * emi(1,98) * β(98,1)][trans(56,2) * emi(2,98) * β(98,2)][trans(56,3) * emi(3,98) * β(98,3)]
β(44,3)[0.07 * 0.06521 * 5.097930258652691e-69][0.03 * 0.05586 * 5.097930258652691e-69][0.9 * 0.02246 * 5.097930258652691e-69] + = 2.3737958023242274e-70

T: 44

i: 1
β(44-1,1) = Σ[[trans(57,1) * emi(1,100) * β(100,1)][trans(57,2) * emi(2,100) * β(100,2)][trans(57,3) * emi(3,100) * β(100,3)]
β(43,1)[0.97 * 0.01482 * 4.4738881092472716e-70][0.0 * 0.01439 * 4.4738881092472716e-70][0.03 * 0.04444 * 4.4738881092472716e-70] + = 2.563806007488185e-71
i: 2
β(44-1,2) = Σ[[trans(57,1) * emi(1,99) * β(99,1)][trans(57,2) * emi(2,99) * β(99,2)][trans(57,3) * emi(3,99) * β(99,3)]
β(43,2)[0.0 * 0.01482 * 1.2436969978001232e-70][0.6 * 0.01439 * 1.2436969978001232e-70][0.4 * 0.04444 * 1.2436969978001232e-70] + = 4.971242851589485e-72
i: 3
β(44-1,3) = Σ[[trans(57,1) * emi(1,98) * β(98,1)][trans(57,2) * emi(2,98) * β(98,2)][trans(57,3) * emi(3,98) * β(98,3)]
β(43,3)[0.07 * 0.01482 * 2.3737958023242274e-70][0.03 * 0.01439 * 2.3737958023242274e-70][0.9 * 0.04444 * 2.3737958023242274e-70] + = 6.014193806901866e-72

T: 43

i: 1
β(43-1,1) = Σ[[trans(58,1) * emi(1,100) * β(100,1)][trans(58,2) * emi(2,100) * β(100,2)][trans(58,3) * emi(3,100) * β(100,3)]
β(42,1)[0.97 * 0.05877 * 2.563806007488185e-71][0.0 * 0.04271 * 2.563806007488185e-71][0.03 * 0.01879 * 2.563806007488185e-71] + = 1.6028612415128445e-72
i: 2
β(43-1,2) = Σ[[trans(58,1) * emi(1,99) * β(99,1)][trans(58,2) * emi(2,99) * β(99,2)][trans(58,3) * emi(3,99) * β(99,3)]
β(42,2)[0.0 * 0.05877 * 4.971242851589485e-72][0.6 * 0.04271 * 4.971242851589485e-72][0.4 * 0.01879 * 4.971242851589485e-72] + = 3.2677838765966145e-73
i: 3
β(43-1,3) = Σ[[trans(58,1) * emi(1,98) * β(98,1)][trans(58,2) * emi(2,98) * β(98,2)][trans(58,3) * emi(3,98) * β(98,3)]
β(42,3)[0.07 * 0.05877 * 6.014193806901866e-72][0.03 * 0.04271 * 6.014193806901866e-72][0.9 * 0.01879 * 6.014193806901866e-72] + = 3.957188512731039e-73

T: 42

i: 1
β(42-1,1) = Σ[[trans(59,1) * emi(1,100) * β(100,1)][trans(59,2) * emi(2,100) * β(100,2)][trans(59,3) * emi(3,100) * β(100,3)]
β(41,1)[0.97 * 0.06409 * 1.6028612415128445e-72][0.0 * 0.06927 * 1.6028612415128445e-72][0.03 * 0.04995 * 1.6028612415128445e-72] + = 1.0842712313821946e-73
i: 2
β(42-1,2) = Σ[[trans(59,1) * emi(1,99) * β(99,1)][trans(59,2) * emi(2,99) * β(99,2)][trans(59,3) * emi(3,99) * β(99,3)]
β(41,2)[0.0 * 0.06409 * 3.2677838765966145e-73][0.6 * 0.06927 * 3.2677838765966145e-73][0.4 * 0.04995 * 3.2677838765966145e-73] + = 2.8920947001616876e-74
i: 3
β(42-1,3) = Σ[[trans(59,1) * emi(1,98) * β(98,1)][trans(59,2) * emi(2,98) * β(98,2)][trans(59,3) * emi(3,98) * β(98,3)]
β(41,3)[0.07 * 0.06409 * 3.957188512731039e-73][0.03 * 0.06927 * 3.957188512731039e-73][0.9 * 0.04995 * 3.957188512731039e-73] + = 4.239644884797175e-74

T: 41

i: 1
β(41-1,1) = Σ[[trans(60,1) * emi(1,100) * β(100,1)][trans(60,2) * emi(2,100) * β(100,2)][trans(60,3) * emi(3,100) * β(100,3)]
β(40,1)[0.97 * 0.06901 * 1.0842712313821946e-73][0.0 * 0.07052 * 1.0842712313821946e-73][0.03 * 0.09536 * 1.0842712313821946e-73] + = 4.3582110899551295e-75
i: 2
β(41-1,2) = Σ[[trans(60,1) * emi(1,99) * β(99,1)][trans(60,2) * emi(2,99) * β(99,2)][trans(60,3) * emi(3,99) * β(99,3)]
β(40,2)[0.0 * 0.06901 * 2.8920947001616876e-74][0.6 * 0.07052 * 2.8920947001616876e-74][0.4 * 0.09536 * 2.8920947001616876e-74] + = 7.035601337145725e-76
i: 3
β(41-1,3) = Σ[[trans(60,1) * emi(1,98) * β(98,1)][trans(60,2) * emi(2,98) * β(98,2)][trans(60,3) * emi(3,98) * β(98,3)]
β(40,3)[0.07 * 0.06901 * 4.239644884797175e-74][0.03 * 0.07052 * 4.239644884797175e-74][0.9 * 0.09536 * 4.239644884797175e-74] + = 3.74251363989781e-76

T: 40

i: 1
β(40-1,1) = Σ[[trans(61,1) * emi(1,100) * β(100,1)][trans(61,2) * emi(2,100) * β(100,2)][trans(61,3) * emi(3,100) * β(100,3)]
β(39,1)[0.97 * 0.04143 * 4.3582110899551295e-75][0.0 * 0.0399 * 4.3582110899551295e-75][0.03 * 0.00066 * 4.3582110899551295e-75] + = 2.7149903196150617e-76
i: 2
β(40-1,2) = Σ[[trans(61,1) * emi(1,99) * β(99,1)][trans(61,2) * emi(2,99) * β(99,2)][trans(61,3) * emi(3,99) * β(99,3)]
β(39,2)[0.0 * 0.04143 * 7.035601337145725e-76][0.6 * 0.0399 * 7.035601337145725e-76][0.4 * 0.00066 * 7.035601337145725e-76] + = 3.6718908529960886e-77
i: 3
β(40-1,3) = Σ[[trans(61,1) * emi(1,98) * β(98,1)][trans(61,2) * emi(2,98) * β(98,2)][trans(61,3) * emi(3,98) * β(98,3)]
β(39,3)[0.07 * 0.04143 * 3.74251363989781e-76][0.03 * 0.0399 * 3.74251363989781e-76][0.9 * 0.00066 * 3.74251363989781e-76] + = 3.7838780794898556e-77

T: 39

i: 1
β(39-1,1) = Σ[[trans(62,1) * emi(1,100) * β(100,1)][trans(62,2) * emi(2,100) * β(100,2)][trans(62,3) * emi(3,100) * β(100,3)]
β(38,1)[0.97 * 0.06409 * 2.7149903196150617e-76][0.0 * 0.06927 * 2.7149903196150617e-76][0.03 * 0.04995 * 2.7149903196150617e-76] + = 1.6935063182681696e-77
i: 2
β(39-1,2) = Σ[[trans(62,1) * emi(1,99) * β(99,1)][trans(62,2) * emi(2,99) * β(99,2)][trans(62,3) * emi(3,99) * β(99,3)]
β(38,2)[0.0 * 0.06409 * 3.6718908529960886e-77][0.6 * 0.06927 * 3.6718908529960886e-77][0.4 * 0.04995 * 3.6718908529960886e-77] + = 2.2821301166043073e-78
i: 3
β(39-1,3) = Σ[[trans(62,1) * emi(1,98) * β(98,1)][trans(62,2) * emi(2,98) * β(98,2)][trans(62,3) * emi(3,98) * β(98,3)]
β(38,3)[0.07 * 0.06409 * 3.7838780794898556e-77][0.03 * 0.06927 * 3.7838780794898556e-77][0.9 * 0.04995 * 3.7838780794898556e-77] + = 2.9953740615396817e-78

T: 38

i: 1
β(38-1,1) = Σ[[trans(63,1) * emi(1,100) * β(100,1)][trans(63,2) * emi(2,100) * β(100,2)][trans(63,3) * emi(3,100) * β(100,3)]
β(37,1)[0.97 * 0.06409 * 1.6935063182681696e-77][0.0 * 0.06927 * 1.6935063182681696e-77][0.03 * 0.04995 * 1.6935063182681696e-77] + = 9.671039457073071e-79
i: 2
β(38-1,2) = Σ[[trans(63,1) * emi(1,99) * β(99,1)][trans(63,2) * emi(2,99) * β(99,2)][trans(63,3) * emi(3,99) * β(99,3)]
β(37,2)[0.0 * 0.06409 * 2.2821301166043073e-78][0.6 * 0.06927 * 2.2821301166043073e-78][0.4 * 0.04995 * 2.2821301166043073e-78] + = 8.099509781463422e-80
i: 3
β(38-1,3) = Σ[[trans(63,1) * emi(1,98) * β(98,1)][trans(63,2) * emi(2,98) * β(98,2)][trans(63,3) * emi(3,98) * β(98,3)]
β(37,3)[0.07 * 0.06409 * 2.9953740615396817e-78][0.03 * 0.06927 * 2.9953740615396817e-78][0.9 * 0.04995 * 2.9953740615396817e-78] + = 1.232480205003369e-79

T: 37

i: 1
β(37-1,1) = Σ[[trans(64,1) * emi(1,100) * β(100,1)][trans(64,2) * emi(2,100) * β(100,2)][trans(64,3) * emi(3,100) * β(100,3)]
β(36,1)[0.97 * 0.05877 * 9.671039457073071e-79][0.0 * 0.04271 * 9.671039457073071e-79][0.03 * 0.01879 * 9.671039457073071e-79] + = 6.509023593151079e-80
i: 2
β(37-1,2) = Σ[[trans(64,1) * emi(1,99) * β(99,1)][trans(64,2) * emi(2,99) * β(99,2)][trans(64,3) * emi(3,99) * β(99,3)]
β(36,2)[0.0 * 0.05877 * 8.099509781463422e-80][0.6 * 0.04271 * 8.099509781463422e-80][0.4 * 0.01879 * 8.099509781463422e-80] + = 8.1282370726976545e-81
i: 3
β(37-1,3) = Σ[[trans(64,1) * emi(1,98) * β(98,1)][trans(64,2) * emi(2,98) * β(98,2)][trans(64,3) * emi(3,98) * β(98,3)]
β(36,3)[0.07 * 0.05877 * 1.232480205003369e-79][0.03 * 0.04271 * 1.232480205003369e-79][0.9 * 0.01879 * 1.232480205003369e-79] + = 1.5420780370885845e-80

T: 36

i: 1
β(36-1,1) = Σ[[trans(65,1) * emi(1,100) * β(100,1)][trans(65,2) * emi(2,100) * β(100,2)][trans(65,3) * emi(3,100) * β(100,3)]
β(35,1)[0.97 * 0.06901 * 6.509023593151079e-80][0.0 * 0.07052 * 6.509023593151079e-80][0.03 * 0.09536 * 6.509023593151079e-80] + = 2.616093151854561e-81
i: 2
β(36-1,2) = Σ[[trans(65,1) * emi(1,99) * β(99,1)][trans(65,2) * emi(2,99) * β(99,2)][trans(65,3) * emi(3,99) * β(99,3)]
β(35,2)[0.0 * 0.06901 * 8.1282370726976545e-81][0.6 * 0.07052 * 8.1282370726976545e-81][0.4 * 0.09536 * 8.1282370726976545e-81] + = 1.986610815382957e-82
i: 3
β(36-1,3) = Σ[[trans(65,1) * emi(1,98) * β(98,1)][trans(65,2) * emi(2,98) * β(98,2)][trans(65,3) * emi(3,98) * β(98,3)]
β(35,3)[0.07 * 0.06901 * 1.5420780370885845e-80][0.03 * 0.07052 * 1.5420780370885845e-80][0.9 * 0.09536 * 1.5420780370885845e-80] + = 2.0765763654129977e-82

T: 35

i: 1
β(35-1,1) = Σ[[trans(66,1) * emi(1,100) * β(100,1)][trans(66,2) * emi(2,100) * β(100,2)][trans(66,3) * emi(3,100) * β(100,3)]
β(34,1)[0.97 * 0.04143 * 2.616093151854561e-81][0.0 * 0.0399 * 2.616093151854561e-81][0.03 * 0.00066 * 2.616093151854561e-81] + = 2.923350382455901e-82
i: 2
β(35-1,2) = Σ[[trans(66,1) * emi(1,99) * β(99,1)][trans(66,2) * emi(2,99) * β(99,2)][trans(66,3) * emi(3,99) * β(99,3)]
β(34,2)[0.0 * 0.04143 * 1.986610815382957e-82][0.6 * 0.0399 * 1.986610815382957e-82][0.4 * 0.00066 * 1.986610815382957e-82] + = 9.327998875442474e-84
i: 3
β(35-1,3) = Σ[[trans(66,1) * emi(1,98) * β(98,1)][trans(66,2) * emi(2,98) * β(98,2)][trans(66,3) * emi(3,98) * β(98,3)]
β(34,3)[0.07 * 0.04143 * 2.0765763654129977e-82][0.03 * 0.0399 * 2.0765763654129977e-82][0.9 * 0.00066 * 2.0765763654129977e-82] + = 2.311584648564676e-83

T: 34

i: 1
β(34-1,1) = Σ[[trans(67,1) * emi(1,100) * β(100,1)][trans(67,2) * emi(2,100) * β(100,2)][trans(67,3) * emi(3,100) * β(100,3)]
β(33,1)[0.97 * 0.11518 * 2.923350382455901e-82][0.0 * 0.07232 * 2.923350382455901e-82][0.03 * 0.00852 * 2.923350382455901e-82] + = 1.820831911908381e-83
i: 2
β(34-1,2) = Σ[[trans(67,1) * emi(1,99) * β(99,1)][trans(67,2) * emi(2,99) * β(99,2)][trans(67,3) * emi(3,99) * β(99,3)]
β(33,2)[0.0 * 0.11518 * 9.327998875442474e-84][0.6 * 0.07232 * 9.327998875442474e-84][0.4 * 0.00852 * 9.327998875442474e-84] + = 8.495449020443624e-85
i: 3
β(34-1,3) = Σ[[trans(67,1) * emi(1,98) * β(98,1)][trans(67,2) * emi(2,98) * β(98,2)][trans(67,3) * emi(3,98) * β(98,3)]
β(33,3)[0.07 * 0.11518 * 2.311584648564676e-83][0.03 * 0.07232 * 2.311584648564676e-83][0.9 * 0.00852 * 2.311584648564676e-83] + = 2.370060075306498e-84

T: 33

i: 1
β(33-1,1) = Σ[[trans(68,1) * emi(1,100) * β(100,1)][trans(68,2) * emi(2,100) * β(100,2)][trans(68,3) * emi(3,100) * β(100,3)]
β(32,1)[0.97 * 0.06409 * 1.820831911908381e-83][0.0 * 0.06927 * 1.820831911908381e-83][0.03 * 0.04995 * 1.820831911908381e-83] + = 1.0393358300541491e-84
i: 2
β(33-1,2) = Σ[[trans(68,1) * emi(1,99) * β(99,1)][trans(68,2) * emi(2,99) * β(99,2)][trans(68,3) * emi(3,99) * β(99,3)]
β(32,2)[0.0 * 0.06409 * 8.495449020443624e-85][0.6 * 0.06927 * 8.495449020443624e-85][0.4 * 0.04995 * 8.495449020443624e-85] + = 3.958380918579247e-86
i: 3
β(33-1,3) = Σ[[trans(68,1) * emi(1,98) * β(98,1)][trans(68,2) * emi(2,98) * β(98,2)][trans(68,3) * emi(3,98) * β(98,3)]
β(32,3)[0.07 * 0.06409 * 2.370060075306498e-84][0.03 * 0.06927 * 2.370060075306498e-84][0.9 * 0.04995 * 2.370060075306498e-84] + = 1.1607581184049653e-85

T: 32

i: 1
β(32-1,1) = Σ[[trans(69,1) * emi(1,100) * β(100,1)][trans(69,2) * emi(2,100) * β(100,2)][trans(69,3) * emi(3,100) * β(100,3)]
β(31,1)[0.97 * 0.05877 * 1.0393358300541491e-84][0.0 * 0.04271 * 1.0393358300541491e-84][0.03 * 0.01879 * 1.0393358300541491e-84] + = 3.06035573427969e-86
i: 2
β(32-1,2) = Σ[[trans(69,1) * emi(1,99) * β(99,1)][trans(69,2) * emi(2,99) * β(99,2)][trans(69,3) * emi(3,99) * β(99,3)]
β(31,2)[0.0 * 0.05877 * 3.958380918579247e-86][0.6 * 0.04271 * 3.958380918579247e-86][0.4 * 0.01879 * 3.958380918579247e-86] + = 5.571999529842437e-87
i: 3
β(32-1,3) = Σ[[trans(69,1) * emi(1,98) * β(98,1)][trans(69,2) * emi(2,98) * β(98,2)][trans(69,3) * emi(3,98) * β(98,3)]
β(31,3)[0.07 * 0.05877 * 1.1607581184049653e-85][0.03 * 0.04271 * 1.1607581184049653e-85][0.9 * 0.01879 * 1.1607581184049653e-85] + = 1.1808496795038377e-86

T: 31

i: 1
β(31-1,1) = Σ[[trans(70,1) * emi(1,100) * β(100,1)][trans(70,2) * emi(2,100) * β(100,2)][trans(70,3) * emi(3,100) * β(100,3)]
β(30,1)[0.97 * 0.03004 * 3.06035573427969e-86][0.0 * 0.05577 * 3.06035573427969e-86][0.03 * 0.09148 * 3.06035573427969e-86] + = 1.2301020275272552e-87
i: 2
β(31-1,2) = Σ[[trans(70,1) * emi(1,99) * β(99,1)][trans(70,2) * emi(2,99) * β(99,2)][trans(70,3) * emi(3,99) * β(99,3)]
β(30,2)[0.0 * 0.03004 * 5.571999529842437e-87][0.6 * 0.05577 * 5.571999529842437e-87][0.4 * 0.09148 * 5.571999529842437e-87] + = 1.3651111189831808e-88
i: 3
β(31-1,3) = Σ[[trans(70,1) * emi(1,98) * β(98,1)][trans(70,2) * emi(2,98) * β(98,2)][trans(70,3) * emi(3,98) * β(98,3)]
β(30,3)[0.07 * 0.03004 * 1.1808496795038377e-86][0.03 * 0.05577 * 1.1808496795038377e-86][0.9 * 0.09148 * 1.1808496795038377e-86] + = 1.0243730718331949e-88

T: 30

i: 1
β(30-1,1) = Σ[[trans(71,1) * emi(1,100) * β(100,1)][trans(71,2) * emi(2,100) * β(100,2)][trans(71,3) * emi(3,100) * β(100,3)]
β(29,1)[0.97 * 0.04143 * 1.2301020275272552e-87][0.0 * 0.0399 * 1.2301020275272552e-87][0.03 * 0.00066 * 1.2301020275272552e-87] + = 7.018204718310273e-89
i: 2
β(30-1,2) = Σ[[trans(71,1) * emi(1,99) * β(99,1)][trans(71,2) * emi(2,99) * β(99,2)][trans(71,3) * emi(3,99) * β(99,3)]
β(29,2)[0.0 * 0.04143 * 1.3651111189831808e-88][0.6 * 0.0399 * 1.3651111189831808e-88][0.4 * 0.00066 * 1.3651111189831808e-88] + = 4.268152554296129e-90
i: 3
β(30-1,3) = Σ[[trans(71,1) * emi(1,98) * β(98,1)][trans(71,2) * emi(2,98) * β(98,2)][trans(71,3) * emi(3,98) * β(98,3)]
β(29,3)[0.07 * 0.04143 * 1.0243730718331949e-88][0.03 * 0.0399 * 1.0243730718331949e-88][0.9 * 0.00066 * 1.0243730718331949e-88] + = 6.967745720496806e-90

T: 29

i: 1
β(29-1,1) = Σ[[trans(72,1) * emi(1,100) * β(100,1)][trans(72,2) * emi(2,100) * β(100,2)][trans(72,3) * emi(3,100) * β(100,3)]
β(28,1)[0.97 * 0.05877 * 7.018204718310273e-89][0.0 * 0.04271 * 7.018204718310273e-89][0.03 * 0.01879 * 7.018204718310273e-89] + = 7.8428421045194385e-90
i: 2
β(29-1,2) = Σ[[trans(72,1) * emi(1,99) * β(99,1)][trans(72,2) * emi(2,99) * β(99,2)][trans(72,3) * emi(3,99) * β(99,3)]
β(28,2)[0.0 * 0.05877 * 4.268152554296129e-90][0.6 * 0.04271 * 4.268152554296129e-90][0.4 * 0.01879 * 4.268152554296129e-90] + = 2.0894975305147072e-91
i: 3
β(29-1,3) = Σ[[trans(72,1) * emi(1,98) * β(98,1)][trans(72,2) * emi(2,98) * β(98,2)][trans(72,3) * emi(3,98) * β(98,3)]
β(28,3)[0.07 * 0.05877 * 6.967745720496806e-90][0.03 * 0.04271 * 6.967745720496806e-90][0.9 * 0.01879 * 6.967745720496806e-90] + = 6.285386315850545e-91

T: 28

i: 1
β(28-1,1) = Σ[[trans(73,1) * emi(1,100) * β(100,1)][trans(73,2) * emi(2,100) * β(100,2)][trans(73,3) * emi(3,100) * β(100,3)]
β(27,1)[0.97 * 0.11518 * 7.8428421045194385e-90][0.0 * 0.07232 * 7.8428421045194385e-90][0.03 * 0.00852 * 7.8428421045194385e-90] + = 2.5197845484877127e-91
i: 2
β(28-1,2) = Σ[[trans(73,1) * emi(1,99) * β(99,1)][trans(73,2) * emi(2,99) * β(99,2)][trans(73,3) * emi(3,99) * β(99,3)]
β(27,2)[0.0 * 0.11518 * 2.0894975305147072e-91][0.6 * 0.07232 * 2.0894975305147072e-91][0.4 * 0.00852 * 2.0894975305147072e-91] + = 1.97172378895774e-92
i: 3
β(28-1,3) = Σ[[trans(73,1) * emi(1,98) * β(98,1)][trans(73,2) * emi(2,98) * β(98,2)][trans(73,3) * emi(3,98) * β(98,3)]
β(27,3)[0.07 * 0.11518 * 6.285386315850545e-91][0.03 * 0.07232 * 6.285386315850545e-91][0.9 * 0.00852 * 6.285386315850545e-91] + = 5.526005366296713e-92

T: 27

i: 1
β(27-1,1) = Σ[[trans(74,1) * emi(1,100) * β(100,1)][trans(74,2) * emi(2,100) * β(100,2)][trans(74,3) * emi(3,100) * β(100,3)]
β(26,1)[0.97 * 0.03296 * 2.5197845484877127e-91][0.0 * 0.0261 * 2.5197845484877127e-91][0.03 * 0.06541 * 2.5197845484877127e-91] + = 1.29610059980084e-92
i: 2
β(27-1,2) = Σ[[trans(74,1) * emi(1,99) * β(99,1)][trans(74,2) * emi(2,99) * β(99,2)][trans(74,3) * emi(3,99) * β(99,3)]
β(26,2)[0.0 * 0.03296 * 1.97172378895774e-92][0.6 * 0.0261 * 1.97172378895774e-92][0.4 * 0.06541 * 1.97172378895774e-92] + = 5.516664734867162e-93
i: 3
β(27-1,3) = Σ[[trans(74,1) * emi(1,98) * β(98,1)][trans(74,2) * emi(2,98) * β(98,2)][trans(74,3) * emi(3,98) * β(98,3)]
β(26,3)[0.07 * 0.03296 * 5.526005366296713e-92][0.03 * 0.0261 * 5.526005366296713e-92][0.9 * 0.06541 * 5.526005366296713e-92] + = 1.0636018376795994e-92

T: 26

i: 1
β(26-1,1) = Σ[[trans(75,1) * emi(1,100) * β(100,1)][trans(75,2) * emi(2,100) * β(100,2)][trans(75,3) * emi(3,100) * β(100,3)]
β(25,1)[0.97 * 0.05171 * 1.29610059980084e-92][0.0 * 0.1033 * 1.29610059980084e-92][0.03 * 0.19429 * 1.29610059980084e-92] + = 7.249205264171318e-94
i: 2
β(26-1,2) = Σ[[trans(75,1) * emi(1,99) * β(99,1)][trans(75,2) * emi(2,99) * β(99,2)][trans(75,3) * emi(3,99) * β(99,3)]
β(25,2)[0.0 * 0.05171 * 5.516664734867162e-93][0.6 * 0.1033 * 5.516664734867162e-93][0.4 * 0.19429 * 5.516664734867162e-93] + = 5.330798351467753e-94
i: 3
β(26-1,3) = Σ[[trans(75,1) * emi(1,98) * β(98,1)][trans(75,2) * emi(2,98) * β(98,2)][trans(75,3) * emi(3,98) * β(98,3)]
β(25,3)[0.07 * 0.05171 * 1.0636018376795994e-92][0.03 * 0.1033 * 1.0636018376795994e-92][0.9 * 0.19429 * 1.0636018376795994e-92] + = 7.230113228473654e-94

T: 25

i: 1
β(25-1,1) = Σ[[trans(76,1) * emi(1,100) * β(100,1)][trans(76,2) * emi(2,100) * β(100,2)][trans(76,3) * emi(3,100) * β(100,3)]
β(24,1)[0.97 * 0.05591 * 7.249205264171318e-94][0.0 * 0.07239 * 7.249205264171318e-94][0.03 * 0.06898 * 7.249205264171318e-94] + = 4.6149784309218535e-95
i: 2
β(25-1,2) = Σ[[trans(76,1) * emi(1,99) * β(99,1)][trans(76,2) * emi(2,99) * β(99,2)][trans(76,3) * emi(3,99) * β(99,3)]
β(24,2)[0.0 * 0.05591 * 5.330798351467753e-94][0.6 * 0.07239 * 5.330798351467753e-94][0.4 * 0.06898 * 5.330798351467753e-94] + = 3.660163033886063e-95
i: 3
β(25-1,3) = Σ[[trans(76,1) * emi(1,98) * β(98,1)][trans(76,2) * emi(2,98) * β(98,2)][trans(76,3) * emi(3,98) * β(98,3)]
β(24,3)[0.07 * 0.05591 * 7.230113228473654e-94][0.03 * 0.07239 * 7.230113228473654e-94][0.9 * 0.06898 * 7.230113228473654e-94] + = 3.6862978181687e-95

T: 24

i: 1
β(24-1,1) = Σ[[trans(77,1) * emi(1,100) * β(100,1)][trans(77,2) * emi(2,100) * β(100,2)][trans(77,3) * emi(3,100) * β(100,3)]
β(23,1)[0.97 * 0.06409 * 4.6149784309218535e-95][0.0 * 0.06927 * 4.6149784309218535e-95][0.03 * 0.04995 * 4.6149784309218535e-95] + = 1.8553558839809938e-96
i: 2
β(24-1,2) = Σ[[trans(77,1) * emi(1,99) * β(99,1)][trans(77,2) * emi(2,99) * β(99,2)][trans(77,3) * emi(3,99) * β(99,3)]
β(23,2)[0.0 * 0.06409 * 3.660163033886063e-95][0.6 * 0.06927 * 3.660163033886063e-95][0.4 * 0.04995 * 3.660163033886063e-95] + = 8.859748565522889e-97
i: 3
β(24-1,3) = Σ[[trans(77,1) * emi(1,98) * β(98,1)][trans(77,2) * emi(2,98) * β(98,2)][trans(77,3) * emi(3,98) * β(98,3)]
β(23,3)[0.07 * 0.06409 * 3.6862978181687e-95][0.03 * 0.06927 * 3.6862978181687e-95][0.9 * 0.04995 * 3.6862978181687e-95] + = 1.9954775003070296e-97

T: 23

i: 1
β(23-1,1) = Σ[[trans(78,1) * emi(1,100) * β(100,1)][trans(78,2) * emi(2,100) * β(100,2)][trans(78,3) * emi(3,100) * β(100,3)]
β(22,1)[0.97 * 0.04143 * 1.8553558839809938e-96][0.0 * 0.0399 * 1.8553558839809938e-96][0.03 * 0.00066 * 1.8553558839809938e-96] + = 1.0103390316308958e-97
i: 2
β(23-1,2) = Σ[[trans(78,1) * emi(1,99) * β(99,1)][trans(78,2) * emi(2,99) * β(99,2)][trans(78,3) * emi(3,99) * β(99,3)]
β(22,2)[0.0 * 0.04143 * 8.859748565522889e-97][0.6 * 0.0399 * 8.859748565522889e-97][0.4 * 0.00066 * 8.859748565522889e-97] + = 4.3987353438339266e-98
i: 3
β(23-1,3) = Σ[[trans(78,1) * emi(1,98) * β(98,1)][trans(78,2) * emi(2,98) * β(98,2)][trans(78,3) * emi(3,98) * β(98,3)]
β(22,3)[0.07 * 0.04143 * 1.9954775003070296e-97][0.03 * 0.0399 * 1.9954775003070296e-97][0.9 * 0.00066 * 1.9954775003070296e-97] + = 2.1573701336517124e-98

T: 22

i: 1
β(22-1,1) = Σ[[trans(79,1) * emi(1,100) * β(100,1)][trans(79,2) * emi(2,100) * β(100,2)][trans(79,3) * emi(3,100) * β(100,3)]
β(21,1)[0.97 * 0.05591 * 1.0103390316308958e-97][0.0 * 0.07239 * 1.0103390316308958e-97][0.03 * 0.06898 * 1.0103390316308958e-97] + = 4.060686729091861e-99
i: 2
β(22-1,2) = Σ[[trans(79,1) * emi(1,99) * β(99,1)][trans(79,2) * emi(2,99) * β(99,2)][trans(79,3) * emi(3,99) * β(99,3)]
β(21,2)[0.0 * 0.05591 * 4.3987353438339266e-98][0.6 * 0.07239 * 4.3987353438339266e-98][0.4 * 0.06898 * 4.3987353438339266e-98] + = 1.0587526984666825e-99
i: 3
β(22-1,3) = Σ[[trans(79,1) * emi(1,98) * β(98,1)][trans(79,2) * emi(2,98) * β(98,2)][trans(79,3) * emi(3,98) * β(98,3)]
β(21,3)[0.07 * 0.05591 * 2.1573701336517124e-98][0.03 * 0.07239 * 2.1573701336517124e-98][0.9 * 0.06898 * 2.1573701336517124e-98] + = 3.584760632228594e-100

T: 21

i: 1
β(21-1,1) = Σ[[trans(80,1) * emi(1,100) * β(100,1)][trans(80,2) * emi(2,100) * β(100,2)][trans(80,3) * emi(3,100) * β(100,3)]
β(20,1)[0.97 * 0.04143 * 4.060686729091861e-99][0.0 * 0.0399 * 4.060686729091861e-99][0.03 * 0.00066 * 4.060686729091861e-99] + = 1.8252037251706452e-100
i: 2
β(21-1,2) = Σ[[trans(80,1) * emi(1,99) * β(99,1)][trans(80,2) * emi(2,99) * β(99,2)][trans(80,3) * emi(3,99) * β(99,3)]
β(20,2)[0.0 * 0.04143 * 1.0587526984666825e-99][0.6 * 0.0399 * 1.0587526984666825e-99][0.4 * 0.00066 * 1.0587526984666825e-99] + = 5.089141651665328e-101
i: 3
β(21-1,3) = Σ[[trans(80,1) * emi(1,98) * β(98,1)][trans(80,2) * emi(2,98) * β(98,2)][trans(80,3) * emi(3,98) * β(98,3)]
β(20,3)[0.07 * 0.04143 * 3.584760632228594e-100][0.03 * 0.0399 * 3.584760632228594e-100][0.9 * 0.00066 * 3.584760632228594e-100] + = 6.04703395161251e-101

T: 20

i: 1
β(20-1,1) = Σ[[trans(81,1) * emi(1,100) * β(100,1)][trans(81,2) * emi(2,100) * β(100,2)][trans(81,3) * emi(3,100) * β(100,3)]
β(19,1)[0.97 * 0.04595 * 1.8252037251706452e-100][0.0 * 0.04801 * 1.8252037251706452e-100][0.03 * 0.14222 * 1.8252037251706452e-100] + = 9.02758218448613e-102
i: 2
β(20-1,2) = Σ[[trans(81,1) * emi(1,99) * β(99,1)][trans(81,2) * emi(2,99) * β(99,2)][trans(81,3) * emi(3,99) * β(99,3)]
β(19,2)[0.0 * 0.04595 * 5.089141651665328e-101][0.6 * 0.04801 * 5.089141651665328e-101][0.4 * 0.14222 * 5.089141651665328e-101] + = 1.3759632464241938e-102
i: 3
β(20-1,3) = Σ[[trans(81,1) * emi(1,98) * β(98,1)][trans(81,2) * emi(2,98) * β(98,2)][trans(81,3) * emi(3,98) * β(98,3)]
β(19,3)[0.07 * 0.04595 * 6.04703395161251e-101][0.03 * 0.04801 * 6.04703395161251e-101][0.9 * 0.14222 * 6.04703395161251e-101] + = 1.0849532626997206e-102

T: 19

i: 1
β(19-1,1) = Σ[[trans(82,1) * emi(1,100) * β(100,1)][trans(82,2) * emi(2,100) * β(100,2)][trans(82,3) * emi(3,100) * β(100,3)]
β(18,1)[0.97 * 0.05092 * 9.02758218448613e-102][0.0 * 0.03962 * 9.02758218448613e-102][0.03 * 0.00687 * 9.02758218448613e-102] + = 3.6949522166144393e-103
i: 2
β(19-1,2) = Σ[[trans(82,1) * emi(1,99) * β(99,1)][trans(82,2) * emi(2,99) * β(99,2)][trans(82,3) * emi(3,99) * β(99,3)]
β(18,2)[0.0 * 0.05092 * 1.3759632464241938e-102][0.6 * 0.03962 * 1.3759632464241938e-102][0.4 * 0.00687 * 1.3759632464241938e-102] + = 4.48282201869158e-104
i: 3
β(19-1,3) = Σ[[trans(82,1) * emi(1,98) * β(98,1)][trans(82,2) * emi(2,98) * β(98,2)][trans(82,3) * emi(3,98) * β(98,3)]
β(18,3)[0.07 * 0.05092 * 1.0849532626997206e-102][0.03 * 0.03962 * 1.0849532626997206e-102][0.9 * 0.00687 * 1.0849532626997206e-102] + = 5.3363903518819776e-104

T: 18

i: 1
β(18-1,1) = Σ[[trans(83,1) * emi(1,100) * β(100,1)][trans(83,2) * emi(2,100) * β(100,2)][trans(83,3) * emi(3,100) * β(100,3)]
β(17,1)[0.97 * 0.0421 * 3.6949522166144393e-103][0.0 * 0.0408 * 3.6949522166144393e-103][0.03 * 0.02568 * 3.6949522166144393e-103] + = 1.8844442159190012e-104
i: 2
β(18-1,2) = Σ[[trans(83,1) * emi(1,99) * β(99,1)][trans(83,2) * emi(2,99) * β(99,2)][trans(83,3) * emi(3,99) * β(99,3)]
β(17,2)[0.0 * 0.0421 * 4.48282201869158e-104][0.6 * 0.0408 * 4.48282201869158e-104][0.4 * 0.02568 * 4.48282201869158e-104] + = 6.9256822130536395e-105
i: 3
β(18-1,3) = Σ[[trans(83,1) * emi(1,98) * β(98,1)][trans(83,2) * emi(2,98) * β(98,2)][trans(83,3) * emi(3,98) * β(98,3)]
β(17,3)[0.07 * 0.0421 * 5.3363903518819776e-104][0.03 * 0.0408 * 5.3363903518819776e-104][0.9 * 0.02568 * 5.3363903518819776e-104] + = 1.0807650041411525e-104

T: 17

i: 1
β(17-1,1) = Σ[[trans(84,1) * emi(1,100) * β(100,1)][trans(84,2) * emi(2,100) * β(100,2)][trans(84,3) * emi(3,100) * β(100,3)]
β(16,1)[0.97 * 0.05171 * 1.8844442159190012e-104][0.0 * 0.1033 * 1.8844442159190012e-104][0.03 * 0.19429 * 1.8844442159190012e-104] + = 1.1877033526300683e-105
i: 2
β(17-1,2) = Σ[[trans(84,1) * emi(1,99) * β(99,1)][trans(84,2) * emi(2,99) * β(99,2)][trans(84,3) * emi(3,99) * β(99,3)]
β(16,2)[0.0 * 0.05171 * 6.9256822130536395e-105][0.6 * 0.1033 * 6.9256822130536395e-105][0.4 * 0.19429 * 6.9256822130536395e-105] + = 5.037820519663376e-106
i: 3
β(17-1,3) = Σ[[trans(84,1) * emi(1,98) * β(98,1)][trans(84,2) * emi(2,98) * β(98,2)][trans(84,3) * emi(3,98) * β(98,3)]

β(16,3)[0.07 * 0.05171 * 1.0807650041411525e-104][0.03 * 0.1033 * 1.0807650041411525e-104][0.9 * 0.19429 * 1.0807650041411525e-104] + = 5.847919886773761e-106

T: 16

i: 1
β(16-1,1) = Σ[[trans(85,1) * emi(1,100) * β(100,1)][trans(85,2) * emi(2,100) * β(100,2)][trans(85,3) * emi(3,100) * β(100,3)]
β(15,1)[0.97 * 0.06409 * 1.1877033526300683e-105][0.0 * 0.06927 * 1.1877033526300683e-105][0.03 * 0.04995 * 1.1877033526300683e-105] + = 6.803693349706449e-107
i: 2
β(16-1,2) = Σ[[trans(85,1) * emi(1,99) * β(99,1)][trans(85,2) * emi(2,99) * β(99,2)][trans(85,3) * emi(3,99) * β(99,3)]
β(15,2)[0.0 * 0.06409 * 5.037820519663376e-106][0.6 * 0.06927 * 5.037820519663376e-106][0.4 * 0.04995 * 5.037820519663376e-106] + = 1.7305215450588525e-107
i: 3
β(16-1,3) = Σ[[trans(85,1) * emi(1,98) * β(98,1)][trans(85,2) * emi(2,98) * β(98,2)][trans(85,3) * emi(3,98) * β(98,3)]
β(15,3)[0.07 * 0.06409 * 5.847919886773761e-106][0.03 * 0.06927 * 5.847919886773761e-106][0.9 * 0.04995 * 5.847919886773761e-106] + = 1.5421006086092416e-107

T: 15

i: 1
β(15-1,1) = Σ[[trans(86,1) * emi(1,100) * β(100,1)][trans(86,2) * emi(2,100) * β(100,2)][trans(86,3) * emi(3,100) * β(100,3)]
β(14,1)[0.97 * 0.05877 * 6.803693349706449e-107][0.0 * 0.04271 * 6.803693349706449e-107][0.03 * 0.01879 * 6.803693349706449e-107] + = 2.6160181336476038e-108
i: 2
β(15-1,2) = Σ[[trans(86,1) * emi(1,99) * β(99,1)][trans(86,2) * emi(2,99) * β(99,2)][trans(86,3) * emi(3,99) * β(99,3)]
β(14,2)[0.0 * 0.05877 * 1.7305215450588525e-107][0.6 * 0.04271 * 1.7305215450588525e-107][0.4 * 0.01879 * 1.7305215450588525e-107] + = 7.053240514885045e-109
i: 3
β(15-1,3) = Σ[[trans(86,1) * emi(1,98) * β(98,1)][trans(86,2) * emi(2,98) * β(98,2)][trans(86,3) * emi(3,98) * β(98,3)]
β(14,3)[0.07 * 0.05877 * 1.5421006086092416e-107][0.03 * 0.04271 * 1.5421006086092416e-107][0.9 * 0.01879 * 1.5421006086092416e-107] + = 4.348236582433563e-109

T: 14

i: 1
β(14-1,1) = Σ[[trans(87,1) * emi(1,100) * β(100,1)][trans(87,2) * emi(2,100) * β(100,2)][trans(87,3) * emi(3,100) * β(100,3)]
β(13,1)[0.97 * 0.03953 * 2.6160181336476038e-108][0.0 * 0.05868 * 2.6160181336476038e-108][0.03 * 0.01557 * 2.6160181336476038e-108] + = 1.105187502074036e-109
i: 2
β(14-1,2) = Σ[[trans(87,1) * emi(1,99) * β(99,1)][trans(87,2) * emi(2,99) * β(99,2)][trans(87,3) * emi(3,99) * β(99,3)]
β(13,2)[0.0 * 0.03953 * 7.053240514885045e-109][0.6 * 0.05868 * 7.053240514885045e-109][0.4 * 0.01557 * 7.053240514885045e-109] + = 3.877386836765721e-110
i: 3
β(14-1,3) = Σ[[trans(87,1) * emi(1,98) * β(98,1)][trans(87,2) * emi(2,98) * β(98,2)][trans(87,3) * emi(3,98) * β(98,3)]
β(13,3)[0.07 * 0.03953 * 4.348236582433563e-109][0.03 * 0.05868 * 4.348236582433563e-109][0.9 * 0.01557 * 4.348236582433563e-109] + = 5.547057990248406e-110

T: 13

i: 1
β(13-1,1) = Σ[[trans(88,1) * emi(1,100) * β(100,1)][trans(88,2) * emi(2,100) * β(100,2)][trans(88,3) * emi(3,100) * β(100,3)]
β(12,1)[0.97 * 0.04294 * 1.105187502074036e-109][0.0 * 0.04257 * 1.105187502074036e-109][0.03 * 0.11935 * 1.105187502074036e-109] + = 6.953774963752594e-111
i: 2
β(13-1,2) = Σ[[trans(88,1) * emi(1,99) * β(99,1)][trans(88,2) * emi(2,99) * β(99,2)][trans(88,3) * emi(3,99) * β(99,3)]
β(12,2)[0.0 * 0.04294 * 3.877386836765721e-110][0.6 * 0.04257 * 3.877386836765721e-110][0.4 * 0.11935 * 3.877386836765721e-110] + = 2.7198217035482002e-111
i: 3
β(13-1,3) = Σ[[trans(88,1) * emi(1,98) * β(98,1)][trans(88,2) * emi(2,98) * β(98,2)][trans(88,3) * emi(3,98) * β(98,3)]
β(12,3)[0.07 * 0.04294 * 5.547057990248406e-110][0.03 * 0.04257 * 5.547057990248406e-110][0.9 * 0.11935 * 5.547057990248406e-110] + = 3.0700761644264742e-111

T: 12

i: 1
β(12-1,1) = Σ[[trans(89,1) * emi(1,100) * β(100,1)][trans(89,2) * emi(2,100) * β(100,2)][trans(89,3) * emi(3,100) * β(100,3)]
β(11,1)[0.97 * 0.06409 * 6.953774963752594e-111][0.0 * 0.06927 * 6.953774963752594e-111][0.03 * 0.04995 * 6.953774963752594e-111] + = 4.368979234364897e-112
i: 2
β(12-1,2) = Σ[[trans(89,1) * emi(1,99) * β(99,1)][trans(89,2) * emi(2,99) * β(99,2)][trans(89,3) * emi(3,99) * β(99,3)]
β(11,2)[0.0 * 0.06409 * 2.7198217035482002e-111][0.6 * 0.06927 * 2.7198217035482002e-111][0.4 * 0.04995 * 2.7198217035482002e-111] + = 1.7438135140811123e-112
i: 3
β(12-1,3) = Σ[[trans(89,1) * emi(1,98) * β(98,1)][trans(89,2) * emi(2,98) * β(98,2)][trans(89,3) * emi(3,98) * β(98,3)]
β(11,3)[0.07 * 0.06409 * 3.0700761644264742e-111][0.03 * 0.06927 * 3.0700761644264742e-111][0.9 * 0.04995 * 3.0700761644264742e-111] + = 1.7486405607381894e-112

T: 11

i: 1
β(11-1,1) = Σ[[trans(90,1) * emi(1,100) * β(100,1)][trans(90,2) * emi(2,100) * β(100,2)][trans(90,3) * emi(3,100) * β(100,3)]
β(10,1)[0.97 * 0.06409 * 4.368979234364897e-112][0.0 * 0.06927 * 4.368979234364897e-112][0.03 * 0.04995 * 4.368979234364897e-112] + = 2.2933461995911321e-113
i: 2
β(11-1,2) = Σ[[trans(90,1) * emi(1,99) * β(99,1)][trans(90,2) * emi(2,99) * β(99,2)][trans(90,3) * emi(3,99) * β(99,3)]
β(10,2)[0.0 * 0.06409 * 1.7438135140811123e-112][0.6 * 0.06927 * 1.7438135140811123e-112][0.4 * 0.04995 * 1.7438135140811123e-112] + = 2.4397891142107646e-113
i: 3
β(11-1,3) = Σ[[trans(90,1) * emi(1,98) * β(98,1)][trans(90,2) * emi(2,98) * β(98,2)][trans(90,3) * emi(3,98) * β(98,3)]
β(10,3)[0.07 * 0.06409 * 1.7486405607381894e-112][0.03 * 0.06927 * 1.7486405607381894e-112][0.9 * 0.04995 * 1.7486405607381894e-112] + = 3.2698750930600847e-113

T: 10

i: 1
β(10-1,1) = Σ[[trans(91,1) * emi(1,100) * β(100,1)][trans(91,2) * emi(2,100) * β(100,2)][trans(91,3) * emi(3,100) * β(100,3)]
β(9,1)[0.97 * 0.05171 * 2.2933461995911321e-113][0.0 * 0.1033 * 2.2933461995911321e-113][0.03 * 0.19429 * 2.2933461995911321e-113] + = 1.4747104902079233e-114
i: 2
β(10-1,2) = Σ[[trans(91,1) * emi(1,99) * β(99,1)][trans(91,2) * emi(2,99) * β(99,2)][trans(91,3) * emi(3,99) * β(99,3)]
β(9,2)[0.0 * 0.05171 * 2.4397891142107646e-113][0.6 * 0.1033 * 2.4397891142107646e-113][0.4 * 0.19429 * 2.4397891142107646e-113] + = 1.6673461952416828e-114
i: 3

β(10-1,3) = Σ[[trans(91,1) * emi(1,98) * β(98,1)][trans(91,2) * emi(2,98) * β(98,2)][trans(91,3) * emi(3,98) * β(98,3)]
β(9,3)[0.07 * 0.05171 * 3.2698750930600847e-113][0.03 * 0.1033 * 3.2698750930600847e-113][0.9 * 0.19429 * 3.2698750930600847e-113] + = 1.6235599962198318e-114

T: 9

i: 1
β(9-1,1) = Σ[[trans(92,1) * emi(1,100) * β(100,1)][trans(92,2) * emi(2,100) * β(100,2)][trans(92,3) * emi(3,100) * β(100,3)]
β(8,1)[0.97 * 0.06409 * 1.4747104902079233e-114][0.0 * 0.06927 * 1.4747104902079233e-114][0.03 * 0.04995 * 1.4747104902079233e-114] + = 9.437484965991783e-116
i: 2
β(9-1,2) = Σ[[trans(92,1) * emi(1,99) * β(99,1)][trans(92,2) * emi(2,99) * β(99,2)][trans(92,3) * emi(3,99) * β(99,3)]
β(8,2)[0.0 * 0.06409 * 1.6673461952416828e-114][0.6 * 0.06927 * 1.6673461952416828e-114][0.4 * 0.04995 * 1.6673461952416828e-114] + = 7.04688380857592e-116
i: 3
β(9-1,3) = Σ[[trans(92,1) * emi(1,98) * β(98,1)][trans(92,2) * emi(2,98) * β(98,2)][trans(92,3) * emi(3,98) * β(98,3)]
β(8,3)[0.07 * 0.06409 * 1.6235599962198318e-114][0.03 * 0.06927 * 1.6235599962198318e-114][0.9 * 0.04995 * 1.6235599962198318e-114] + = 4.23443914922258e-116

T: 8

i: 1
β(8-1,1) = Σ[[trans(93,1) * emi(1,100) * β(100,1)][trans(93,2) * emi(2,100) * β(100,2)][trans(93,3) * emi(3,100) * β(100,3)]
β(7,1)[0.97 * 0.06521 * 9.437484965991783e-116][0.0 * 0.05586 * 9.437484965991783e-116][0.03 * 0.02246 * 9.437484965991783e-116] + = 5.4038871505621374e-117
i: 2
β(8-1,2) = Σ[[trans(93,1) * emi(1,99) * β(99,1)][trans(93,2) * emi(2,99) * β(99,2)][trans(93,3) * emi(3,99) * β(99,3)]
β(7,2)[0.0 * 0.06521 * 7.04688380857592e-116][0.6 * 0.05586 * 7.04688380857592e-116][0.4 * 0.02246 * 7.04688380857592e-116] + = 2.1240948912412344e-117
i: 3
β(8-1,3) = Σ[[trans(93,1) * emi(1,98) * β(98,1)][trans(93,2) * emi(2,98) * β(98,2)][trans(93,3) * emi(3,98) * β(98,3)]
β(7,3)[0.07 * 0.06521 * 4.23443914922258e-116][0.03 * 0.05586 * 4.23443914922258e-116][0.9 * 0.02246 * 4.23443914922258e-116] + = 1.1946264207802498e-117

T: 7

i: 1
β(7-1,1) = Σ[[trans(94,1) * emi(1,100) * β(100,1)][trans(94,2) * emi(2,100) * β(100,2)][trans(94,3) * emi(3,100) * β(100,3)]
β(6,1)[0.97 * 0.05877 * 5.4038871505621374e-117][0.0 * 0.04271 * 5.4038871505621374e-117][0.03 * 0.01879 * 5.4038871505621374e-117] + = 2.1719020691148715e-118
i: 2
β(7-1,2) = Σ[[trans(94,1) * emi(1,99) * β(99,1)][trans(94,2) * emi(2,99) * β(99,2)][trans(94,3) * emi(3,99) * β(99,3)]
β(6,2)[0.0 * 0.05877 * 2.1240948912412344e-117][0.6 * 0.04271 * 2.1240948912412344e-117][0.4 * 0.01879 * 2.1240948912412344e-117] + = 5.116621307140115e-119
i: 3
β(7-1,3) = Σ[[trans(94,1) * emi(1,98) * β(98,1)][trans(94,2) * emi(2,98) * β(98,2)][trans(94,3) * emi(3,98) * β(98,3)]
β(6,3)[0.07 * 0.05877 * 1.1946264207802498e-117][0.03 * 0.04271 * 1.1946264207802498e-117][0.9 * 0.01879 * 1.1946264207802498e-117] + = 1.8923962804104485e-119

T: 6

i: 1
β(6-1,1) = Σ[[trans(95,1) * emi(1,100) * β(100,1)][trans(95,2) * emi(2,100) * β(100,2)][trans(95,3) * emi(3,100) * β(100,3)]
β(5,1)[0.97 * 0.04143 * 2.1719020691148715e-118][0.0 * 0.0399 * 2.1719020691148715e-118][0.03 * 0.00066 * 2.1719020691148715e-118] + = 9.737863152838869e-120
i: 2
β(6-1,2) = Σ[[trans(95,1) * emi(1,99) * β(99,1)][trans(95,2) * emi(2,99) * β(99,2)][trans(95,3) * emi(3,99) * β(99,3)]
β(5,2)[0.0 * 0.04143 * 5.116621307140115e-119][0.6 * 0.0399 * 5.116621307140115e-119][0.4 * 0.00066 * 5.116621307140115e-119] + = 1.6951412830251554e-120
i: 3
β(6-1,3) = Σ[[trans(95,1) * emi(1,98) * β(98,1)][trans(95,2) * emi(2,98) * β(98,2)][trans(95,3) * emi(3,98) * β(98,3)]
β(5,3)[0.07 * 0.04143 * 1.8923962804104485e-119][0.03 * 0.0399 * 1.8923962804104485e-119][0.9 * 0.00066 * 1.8923962804104485e-119] + = 8.634068352608319e-121

T: 5

i: 1
β(5-1,1) = Σ[[trans(96,1) * emi(1,100) * β(100,1)][trans(96,2) * emi(2,100) * β(100,2)][trans(96,3) * emi(3,100) * β(100,3)]
β(4,1)[0.97 * 0.04621 * 9.737863152838869e-120][0.0 * 0.05409 * 9.737863152838869e-120][0.03 * 0.00457 * 9.737863152838869e-120] + = 1.9105321462731017e-121
i: 2
β(5-1,2) = Σ[[trans(96,1) * emi(1,99) * β(99,1)][trans(96,2) * emi(2,99) * β(99,2)][trans(96,3) * emi(3,99) * β(99,3)]
β(4,2)[0.0 * 0.04621 * 1.6951412830251554e-120][0.6 * 0.05409 * 1.6951412830251554e-120][0.4 * 0.00457 * 1.6951412830251554e-120] + = 2.082926088285258e-122
i: 3
β(5-1,3) = Σ[[trans(96,1) * emi(1,98) * β(98,1)][trans(96,2) * emi(2,98) * β(98,2)][trans(96,3) * emi(3,98) * β(98,3)]
β(4,3)[0.07 * 0.04621 * 8.634068352608319e-121][0.03 * 0.05409 * 8.634068352608319e-121][0.9 * 0.00457 * 8.634068352608319e-121] + = 2.489410267764199e-122

T: 4

i: 1
β(4-1,1) = Σ[[trans(97,1) * emi(1,100) * β(100,1)][trans(97,2) * emi(2,100) * β(100,2)][trans(97,3) * emi(3,100) * β(100,3)]
β(3,1)[0.97 * 0.02019 * 1.9105321462731017e-121][0.0 * 0.01597 * 1.9105321462731017e-121][0.03 * 0.01328 * 1.9105321462731017e-121] + = 9.728081132804034e-123
i: 2
β(4-1,2) = Σ[[trans(97,1) * emi(1,99) * β(99,1)][trans(97,2) * emi(2,99) * β(99,2)][trans(97,3) * emi(3,99) * β(99,3)]
β(3,2)[0.0 * 0.02019 * 2.082926088285258e-122][0.6 * 0.01597 * 2.082926088285258e-122][0.4 * 0.01328 * 2.082926088285258e-122] + = 3.225667673214828e-123
i: 3
β(4-1,3) = Σ[[trans(97,1) * emi(1,98) * β(98,1)][trans(97,2) * emi(2,98) * β(98,2)][trans(97,3) * emi(3,98) * β(98,3)]
β(3,3)[0.07 * 0.02019 * 2.489410267764199e-122][0.03 * 0.01597 * 2.489410267764199e-122][0.9 * 0.01328 * 2.489410267764199e-122] + = 5.1091128887775904e-123

T: 3

i: 1
β(3-1,1) = Σ[[trans(98,1) * emi(1,100) * β(100,1)][trans(98,2) * emi(2,100) * β(100,2)][trans(98,3) * emi(3,100) * β(100,3)]
β(2,1)[0.97 * 0.05171 * 9.728081132804034e-123][0.0 * 0.1033 * 9.728081132804034e-123][0.03 * 0.19429 * 9.728081132804034e-123] + = 4.234852684255951e-124
i: 2
β(3-1,2) = Σ[[trans(98,1) * emi(1,99) * β(99,1)][trans(98,2) * emi(2,99) * β(99,2)][trans(98,3) * emi(3,99) * β(99,3)]
β(2,2)[0.0 * 0.05171 * 3.225667673214828e-123][0.6 * 0.1033 * 3.225667673214828e-123][0.4 * 0.19429 * 3.225667673214828e-123] + = 3.262990530194953e-124

i: 3
β(3-1,3) = Σ[[trans(98,1) * emi(1,98) * β(98,1)][trans(98,2) * emi(2,98) * β(98,2)][trans(98,3) * emi(3,98) * β(98,3)]
β(2,3)[0.07 * 0.05171 * 5.1091128887775904e-123][0.03 * 0.1033 * 5.1091128887775904e-123][0.9 * 0.19429 * 5.1091128887775904e-123] + = 5.8215552740248984e-124

T: 2

i: 1
β(2-1,1) = Σ[[trans(99,1) * emi(1,100) * β(100,1)][trans(99,2) * emi(2,100) * β(100,2)][trans(99,3) * emi(3,100) * β(100,3)]
β(1,1)[0.97 * 0.04294 * 4.234852684255951e-124][0.0 * 0.04257 * 4.234852684255951e-124][0.03 * 0.11935 * 4.234852684255951e-124] + = 1.906199014913523e-125
i: 2
β(2-1,2) = Σ[[trans(99,1) * emi(1,99) * β(99,1)][trans(99,2) * emi(2,99) * β(99,2)][trans(99,3) * emi(3,99) * β(99,3)]
β(1,2)[0.0 * 0.04294 * 3.262990530194953e-124][0.6 * 0.04257 * 3.262990530194953e-124][0.4 * 0.11935 * 3.262990530194953e-124] + = 1.1653889770786451e-125
i: 3
β(2-1,3) = Σ[[trans(99,1) * emi(1,98) * β(98,1)][trans(99,2) * emi(2,98) * β(98,2)][trans(99,3) * emi(3,98) * β(98,3)]
β(1,3)[0.07 * 0.04294 * 5.8215552740248984e-124][0.03 * 0.04257 * 5.8215552740248984e-124][0.9 * 0.11935 * 5.8215552740248984e-124] + = 4.293738955317449e-126

Termination

$$P\left(q_{t^*} = S_k \mid O\right) = \frac{\alpha\left(t^*, k\right) \cdot \beta\left(t^*, k\right)}{P(O)}$$

P(q_2 = M|MEILCEDNTSLSSIPNSLMQVDGDSGLYRNDFNSRDANSSDASNWTIDGENRTNLSFEGYLPPTCLSILHLQEKNWSALLTAVVIILTIAGNILVIMAVS) = α(2,3) * β(2,3) / P(O)
P(q_2 = M|MEILCEDNTSLSSIPNSLMQVDGDSGLYRNDFNSRDANSSDASNWTIDGENRTNLSFEGYLPPTCLSILHLQEKNWSALLTAVVIILTIAGNILVIMAVS) = (1.2652959000000002e-05 * 5.8215552740248984e-124)/7.698066770155403e-127 = 0.009568623187842759

Plot

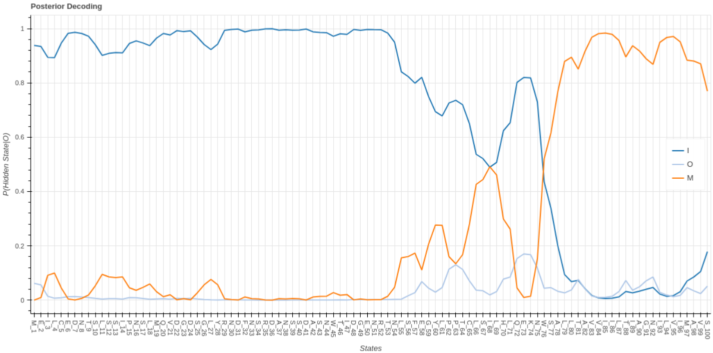

Matrix of β values

	M_1	E_2	I_3	L_4	C_5	E_6	D_7	N_8	T_9	S_{10}	L_{11}	S_{12}	S_{13}	I_{14}	P_{15}	N_{16}	S_{17}
**I**	0.000000	0.000000	0.000000	0.000000	0.000000	0.000000	0.000000	0.000000	0.000000	0.000000	0.000000	0.000000	0.000000	0.000000	0.000000	0.000000	0.00[illegible]
**O**	0.000000	0.000000	0.000000	0.000000	0.000000	0.000000	0.000000	0.000000	0.000000	0.000000	0.000000	0.000000	0.000000	0.000000	0.000000	0.000000	0.00[illegible]
**M**	0.000000	0.000000	0.000000	0.000000	0.000000	0.000000	0.000000	0.000000	0.000000	0.000000	0.000000	0.000000	0.000000	0.000000	0.000000	0.000000	0.00[illegible]

HMMTeacher Supplementary File 1 - Hidden Markov Modeling with HMMTeacher
